# 2-Trifluoromethyl-6-mercurianiline Nucleotide, a Sensitive ^19^F NMR Probe for Hg(II)-mediated Base Pairing

**DOI:** 10.1021/acs.joc.1c02056

**Published:** 2021-12-14

**Authors:** Asmo Aro-Heinilä, Assi Lepistö, Antti Äärelä, Tuomas Antti Lönnberg, Pasi Virta

**Affiliations:** Department of Chemistry, University of Turku, Henrikinkatu 2, 20500 Turku, Finland

## Abstract

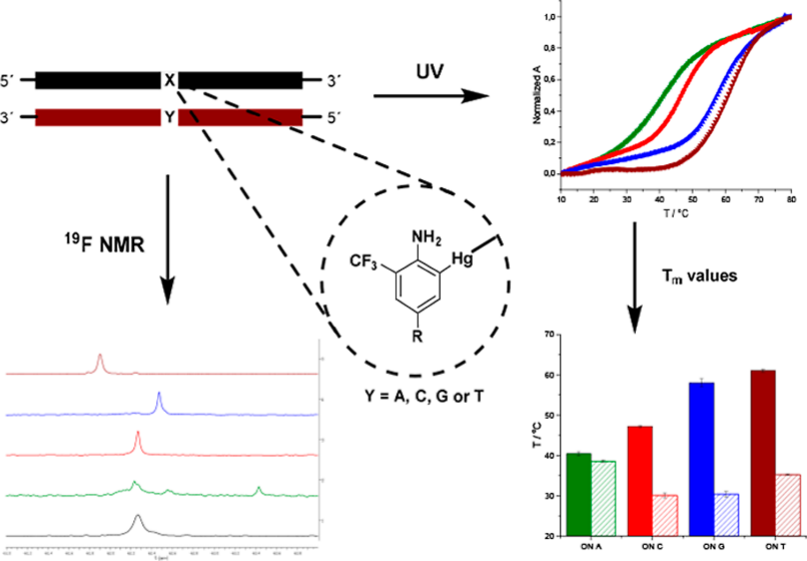

A 2-trifluoromethylaniline C-nucleoside was synthesized, incorporated in the middle of an oligonucleotide, and mercurated. The affinity of the mercurated oligonucleotide toward complementary strands placing each of the canonical nucleobases opposite to the organomercury nucleobase analogue was examined by ultraviolet (UV), circular dichroism (CD), and ^19^F NMR spectroscopy analyses. According to the UV melting profile analysis, the organomercury nucleobase analogue showed increased affinities in the order T > G > C > A. The CD profiles indicated the typical B-type helix in each case. The ^19^F resonance signal proved sensitive for the local environmental changes, showing clearly distinct signals for the duplexes with different opposing nucleobases. Furthermore, valuable information on the mercurated oligonucleotide and its binding to complementary strands at varying temperature could be obtained by ^19^F NMR spectroscopy.

## Introduction

Metal-mediated base pairing has been the subject of numerous studies during the last two decades as metal ions are able to bind site-specifically between the nucleobases in double helical nucleic acids.^1−6^ Most studies consider metal-mediated base pairing between natural nucleobases such as T–Hg–T and C–Ag–C,^7−16^ but many artificial nucleobases capable of metal-mediated base pairing have also been described.^17−23^ Possible applications of metal-mediated base pairing are molecular wires, sensors for metal ions, expansion of the genetic code,^3,24−27^ and improved targeting of relevant nucleic acid structures for diagnostic and therapeutic purposes.^28,29^

In order to target biological structures, metal-mediated base pairing between two natural nucleobases or between one natural and one artificial nucleobase is required. However, the applicability of most coordination complexes in biological systems is limited as the dissociation of the complexes in a metal-deficient environment is more likely than the desired metal-mediated base pairing. The premature dissociation may be prevented by stable organometallic complexes. They do not require similar external metal ion content and may thus be more applicable in biological systems.^30−40^

In our previous work, a 3-fluoro-2-mercuri-6-methylaniline nucleoside was introduced.^41^ The nucleoside was used for the assembly of high-affinity DNA probes, which exhibited different affinities for thymine, guanine cytosine, and adenine, applicable for the detection of single-nucleoside polymorphisms. The 3-fluoro group of this nucleoside served as a potential hydrogen bond acceptor and created an appropriate shape complementarity, which had a beneficial effect for the binding and its selectivity. Furthermore, it proved a valuable spin label, which we utilized for the ^19^F NMR characterization of the metal-mediated base pairs. The sensitivity of the ^19^F NMR resonances was enough to perform the NMR analyses at tens of micromolar concentrations.

**Figure 1:**
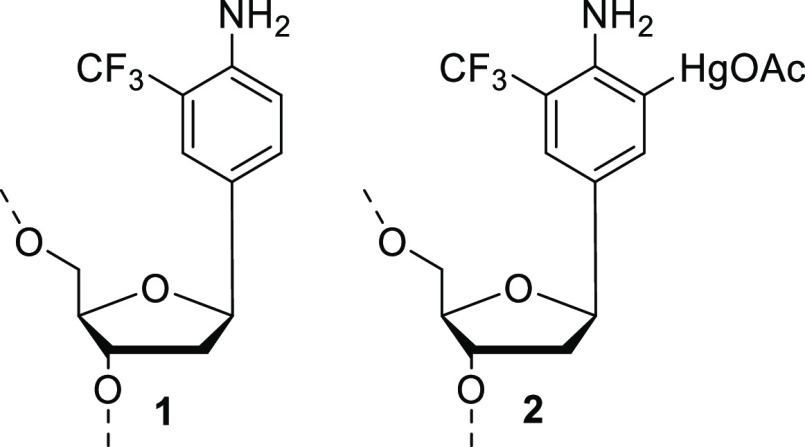
Structures of **1** and its mercurated analogue **2**.

In the present work, we incorporated a trifluoromethyl group to the mercurianiline nucleoside (Figure 1). The trifluoromethyl group with three chemically equivalent ^19^F nuclei at C2 acts as a sensitive ^19^F spin label,^42−44^ but it points out toward the major groove^45^ and, hence, should not contribute much on the stability, base pairing, or conformation of the double helices formed.^46−48^ This nucleotide analogue was expected to provide valuable ^19^F NMR-based information on Hg(II)-mediated base pairs even at micromolar concentrations. To evaluate these hypotheses, phosphoramidite building block **3** was synthesized and incorporated into an oligonucleotide by automated solid phase synthesis. The oligonucleotide was mercurated (**1** → **2**) and exposed to complementary oligonucleotides. The stability and helicity of the duplexes were examined by ultraviolet (UV) and circular dichroism (CD) spectroscopy. Then, the applicability of the probe as a ^19^F spin label to obtain more detailed information on the Hg(II)-mediated base pairs and their dissociation with increasing temperature was studied.

## Results and Discussion

**Scheme 1:**
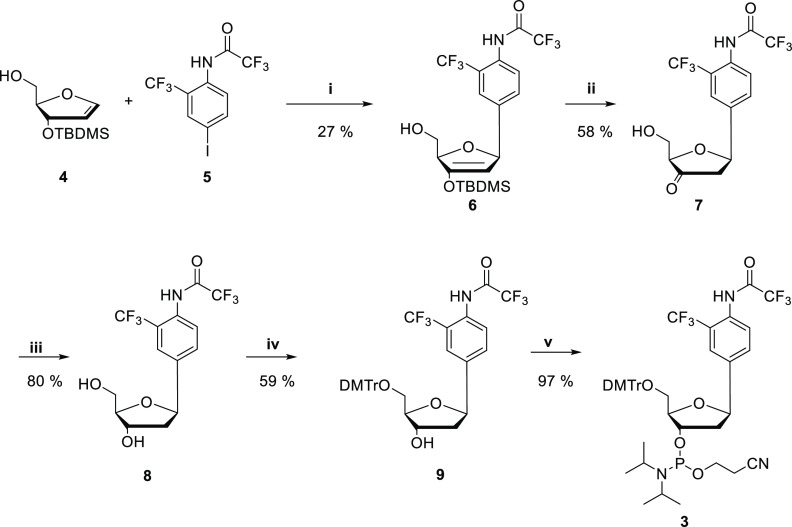
Synthesis of Phosphoramidite Block **1** Conditions: (i) Pd[(*t*-Bu)_3_P]_2_, *N*,*N*-dicyclohexylmethylamine, dioxane, 70 °C; (ii) Et_3_N·3HF, THF, 0 °C; (iii) NaBH(OAc)_3_, MeCN, AcOH; (iv) DMTrCl, pyridine; (v) 2-cyanoethyl *N*,*N*-diisopropylchlorophosphoramidite, Et_3_N, CH_2_Cl_2_.

The nucleoside analogue and its phosphoramidite building block **3** were synthesized as described previously for related compounds (Scheme 1).^41,49^ First, Heck coupling between {(2*R*,3*S*)-3-[(*tert*-butyldimethylsilyl)oxy]-2,3-dihydrofuran-2-yl}-methanol^50^ (**4**) and 2-trifluoromethyl-4-iodo-aniline (**5**) was carried out, yielding 27% of the desired product **6** as a pure β-anomer.^51^ The *tert*-butyldimethylsilyl protection of **6** was removed, and the resulting ketone (**7**) was selectively reduced to a single diastereoisomer of **8**.^52^ Finally, **3** was obtained by the dimethoxytritylation of the 5′-OH group of **8** and phosphitylation of the 3′-OH group of **9** by conventional methods. Detailed synthesis protocols are presented in the Supporting Information. An 11mer oligonucleotide [**ON(1)**], containing 2-trifluoromethylaniline nucleotide (**1**) in the middle of the sequence, was synthesized by a DNA/RNA synthesizer (Table 1). Standard phosphoramidite protocols were followed, except that the coupling time for **3** was prolonged to 300 s and phenoxyacetic anhydride was used for the capping step to prevent the acetylation of the amino group of **3**.^49,53^ 5-Methylcytosines were used on the sequence of **ON(1)** to direct the mercuration only to the C2 of **1**. After the synthesis, oligonucleotides were cleaved from the support by standard ammonolysis and purified by reverse-phase high-performance liquid chromatography (RP-HPLC) (Figure S1). **ON(1)** was identified by elestrospray ionization (ESI)–time-of-flight mass spectrometry (TOF MS) (Figure S2).

**Table 1:** Oligonucleotide Sequences Used in This Study^a^

	sequence
ON(1)	5′-C^m^GAGC^m^XC^m^TGGC^m^-3′
ON(2)	5′-C^m^GAGC^m^X^Hg^C^m^TGGC^m^-3′
ON(A)	5′-GCCAGAGCTCG-3′
ON(C)	5′-GCCAGCGCTCG-3′
ON(G)	5′-GCCAGGGCTCG-3′
ON(T)	5′-GCCAGTGCTCG-3′

a X represents 2-trifluoromethylaniline C-nucleoside and X^Hg^ its mercurated analogue.

**ON(1)** (100 μM solution) was mercurated using 30 equiv of Hg(OAc)_2_ in 5 mM aqueous NaOAc. The mixture was incubated overnight at 55 °C, treated with a 0.1 M aqueous solution of ethylenediaminetetraacetic acid (EDTA), and then subjected to RP-HPLC purification. The authenticity of the mercurated product [**ON(2)**] was verified by MS (ESI–TOF) (Figure S5), and the site of the mercuration was verified by the enzymatic degradation of the sequence, followed by the mass spectrometric analysis of the fragments (Figure S7). The mercurated residue **2** could be identified as a dinucleotide fragment. Due to the deactivation of the CF_3_-group, the mercuration of **1** was slow compared to that of anilines in general.^41,45^ It may be worth mentioning that the amount of degraded oligonucleotides increased over prolonged exposure to the reaction conditions. On reaching a satisfactory conversion (after overnight treatment), EDTA solution was added to the reaction mixture in order to chelate the excess mercury ions. The EDTA treatment also facilitated the RP-HPLC purification of product **ON(2)**, presumably by removing unspecifically bound mercury ions from the oligonucleotide (Figure S3). After this protocol, a sufficient amount (∼33% per batch) of **ON(2)** was obtained for hybridization studies.

**Figure 2:**
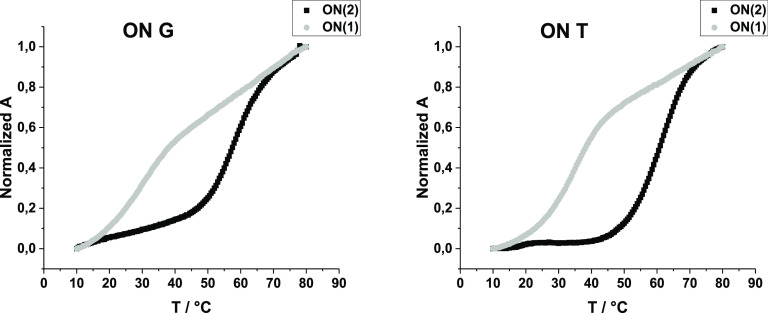
UV melting profiles of **ON(2)·ON(G/T)**. Sample composition: [oligonucleotides] = 2.0 μM; pH = 7.0 (10 mM cacodylate buffer); and *I*(NaCl) = 0.1 M.

**Figure 3:**
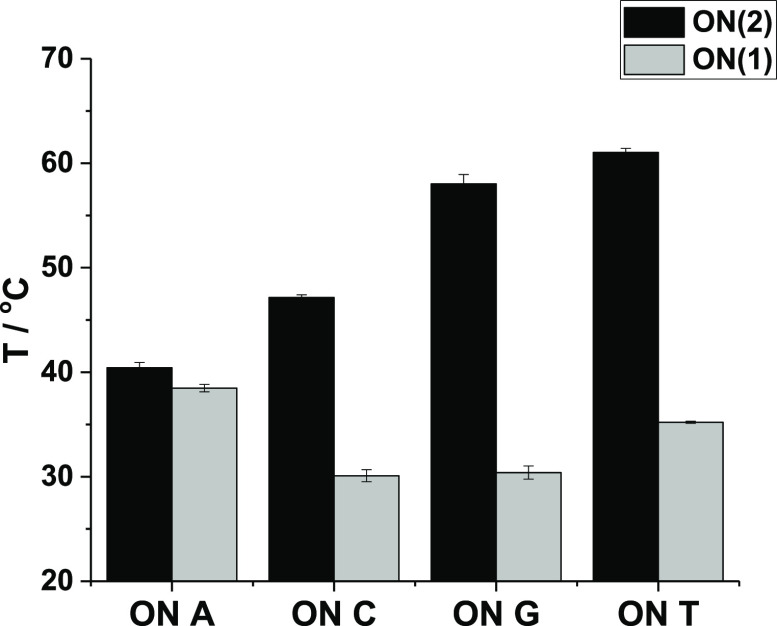
Melting temperatures of all **ON(2)·ON(A/C/G/T)** samples. Black bars represent mercurated duplexes, and gray bars represent unmercurated duplexes. Sample composition: [oligonucleotides] = 2.0 μM; pH = 7.0 (10 mM cacodylate buffer); and *I*(NaCl) = 0.1 M.

The ability of **ON(1)** and **ON(2)** to form double helices was first examined by UV melting temperature analysis. Identical samples of **ON(2)** and **ON(1)** were prepared containing 2.0 μmol L^–1^ oligonucleotide and 1 equiv of the complementary strand (where either the A, C, G, or T nucleobase was placed opposite to the **2** or **1** residue, Table 1) in a 10 mmol L^–1^ cacodylate buffer (pH 7.0, *I* = 0.10 M adjusted with NaCl). Melting curves were obtained by measuring the absorbance at 260 nm over a temperature range of 10–80 °C. All melting profiles were monophasic and sigmoidal (Figures 2 & S8). Melting temperatures of **ON(2)·ON(A)** (*T*_m_ = 40.4 ± 0.5 °C) and **ON(2)·ON(C)** (*T*_m_ = 47.2 ± 0.3 °C) were equal or slightly higher than those of the corresponding unmercurated duplexes. On the other hand, markedly increased melting temperatures were observed with **ON(2)·ON(G)** (*T*_m_ = 58.0 ± 0.9 °C) and **ON(2)·ON(T)** (*T*_m_ = 61.0 ± 0.4 °C), exceeding those of fully matched natural duplexes of the same sequences (57–59 °C) (Figure 2).^31^ Different nucleobases opposite to **2** could be well-discriminated by varying *T*_m_ values (Figure 3), although the difference observed between **ON(2)·ON(G)** and **ON(2)·ON(T)** was small (3 °C). The results are similar to those previously published for the 3-fluoro-2-mercuri-6-methylaniline nucleoside in the middle of the same sequence,^41^ albeit with a lower affinity to the complementary **ON(T)** strand.

From the UV melting data, van’t Hoff plots were constructed, and enthalpies and entropies of hybridization were extracted for all duplexes studied (Tables 2 and 3).^54^ As previously reported, Hg(II)-mediated base pairing should lead to less negative enthalpies and entropies as fewer bonds are formed than in Watson–Crick base pairing, and the dehydration of the Hg(II) ion releases water molecules into the bulk solvent.^13,31,55,56^ Furthermore, the bulky CF_3_ group could be expected to cause additional hydrophobic interactions.^46,48,57^ In the present case, however, more negative enthalpies and entropies were observed with **ON(2)** than those with **ON(1)**, especially when the modified base was paired with a pyrimidine base. The greater length of the mercury-mediated base pairs (4 Å for T–Hg^II^–T)^58^ than that of the Watson–Crick base pairs (<3 Å) might explain the more negative enthalpy and entropy with pyrimidine bases. When pairing with pyrimidines, **2** would get buried deeper within the base stack, leading to the restricted rotation of the trifluoromethane substituent and thus a higher entropic penalty. In contrast, pairing with the longer purine bases could place the trifluoromethyl substituent in the major groove, allowing free rotation. Restricted rotation might promote the formation of a F–H–N hydrogen bond between the amine and CF_3_ groups, previously observed with benzanilides,^59,60^ which, in turn, would contribute toward more negative enthalpy.

**Table 2:** Enthalpies of Hybridization for Oligonucleotide Duplexes

	Δ*H*°/kJ mol^–1^			
	ON(A)	ON(C)	ON(G)	ON(T)
ON(2)	–260 ± 2	–373 ± 6	–260 ± 6	–310 ± 2
ON(1)	–250 ± 1	–276 ± 3	–251 ± 5	–252 ± 2

**Table 3:** Entropies of Hybridization for Oligonucleotide Duplexes

	Δ*S*°/J mol^–1^			
	ON(A)	ON(C)	ON(G)	ON(T)
ON(2)	–716 ± 7	–1050 ± 18	–662 ± 19	–813 ± 8
ON(1)	–694 ± 4	–798 ± 9	–717 ± 15	–70 ± 6

**Figure 4:**
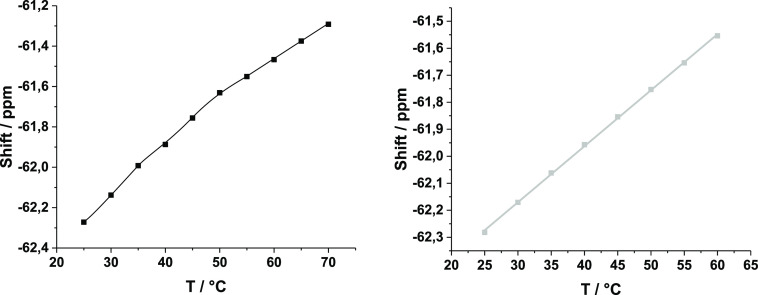
^19^F NMR shifts of **ON(2)** (left) and **ON(1)** (right) as a function of temperature. Sample composition: [oligonucleotides] = 10 μM; pH = 7.0 (10 mM cacodylate buffer, D_2_O–H_2_O, 1:9, v/v); and *I*(NaCl) = 0.1 M.

^19^F NMR properties of **1** and **2** were studied using samples of 10 μM **ON(2)** and 5 μM **ON(1)** in a 10 mM cacodylate buffer (pH = 7.0, D_2_O–H_2_O, 1:9, v/v, *I* = 0.1 M adjusted with NaCl) at 25 °C. The mercuration itself (**1** → **2**) did not cause changes in the ^19^F NMR resonance. **ON(2)** was observed as a broad signal with the same shift as that of **ON(1)** at −62.28 ppm (Figure S6). Minor signals upfield of the major ones was also detected in **ON(1)** and **ON(2)** samples, which may be attributed to syn and anti conformers of **1** and **2** residues. We also measured the ^19^F NMR spectra of **ON(1)** and **ON(2)** with increasing temperature. A linear temperature-dependent passive shift was observed in the case of **ON1**, but a slightly sigmoidal one was observed in the case of **ON(2)** (Figure 4). This indicates an intramolecular mercury-mediated binding of **2**, likely occurring to the thymine and guanine bases on **ON(2)**.

**Figure 5:**
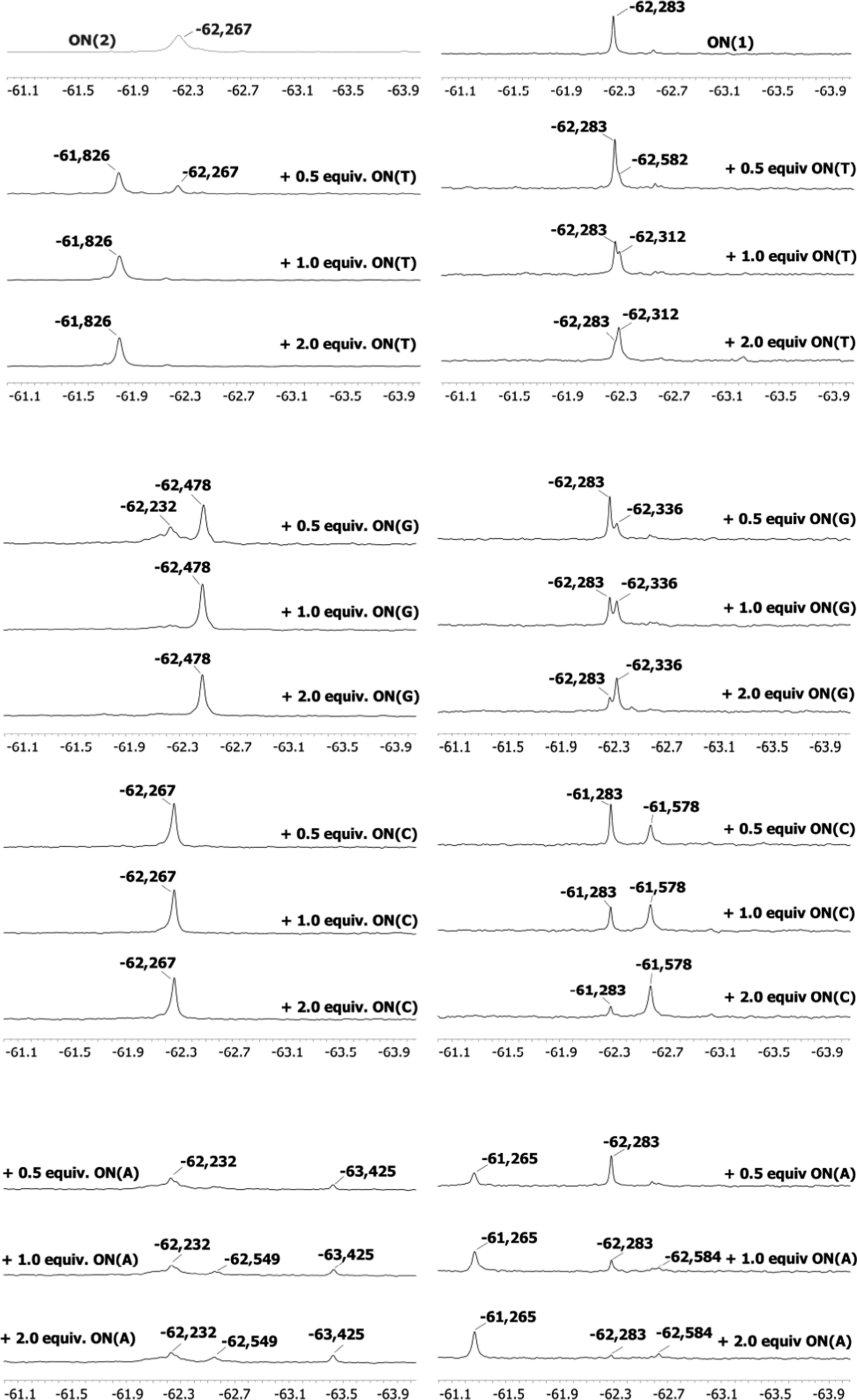
^19^F NMR titration of **ON(2)** (left) and **ON(1)** (right) with 0.5, 1.0, and 2.0 equiv of **ON(Y)** at 25 °C. Sample composition: [oligonucleotides] = 10 μM; pH = 7.0 (10 mM cacodylate buffer, D_2_O–H_2_O, 1:9, v/v); and *I*(NaCl) = 0.1 M.

**ON(1)** was then titrated with complementary strands. Signals upfield in the case of **ON(T)** (partly overlapping), **ON(G)**, and **ON(C)** and a signal downfield in the case of **ON(A)** (Figure 5) were observed. In each case, the initial signal of **ON(1)** did not disappear entirely upon the addition of an equimolar amount of the complementary strand due to the low duplex stability.^61^ These ^19^F NMR data acted as a useful control that would expose plausible non-mercury-related signals in the experiments with **ON(2)** below.

**ON(2)** was next titrated using 0.5, 1.0, and 2.0 equiv of the complementary strands. Titration with **ON(G)** and **ON(T)** resulted clear distinct signals downfield and upfield, at −62.48 and −62.83 ppm, respectively. The initial signal of **ON(2)** disappeared completely after the addition of 1 equiv of **ON(G)** or **ON(T)**. Titration with **ON(A)** gave an indication of weak and obscure binding. Two of the signals may be attributed to different binding modes between **2** and N1/N7 of adenine (Figure 5) but, in general, obscurity dominated the binding. Resonances around the initial **ON(2)** signal was observed even in the presence of 2 equiv of **ON(A)**. No apparent changes in the ^19^F NMR resonance were observed with **ON(C)**. However, two distinct signals could be observed at an increased temperature, which confirmed that the signals of **ON(2)** and **ON(2)·ON(C)** overlapped at 25 °C (Figures 5 and S18).

To clarify the interpretation of ^19^F NMR spectra and to gain more detailed information on the local environment, measurements at elevated temperatures (from 25 °C up to 80 °C for the mercurated duplexes and from 25 °C up to 60 °C for the unmercurated duplexes) were carried out. All signals of **ON(1)·ON(T/A/G/C)** duplexes shifted downfield upon increasing the temperature (a temperature-dependent passive shift of 0.02 ppm K^–1^, Figures S21–S24). Rough estimations of the melting temperatures based on the peak areas of the ^19^F NMR resonances were in line with the global melting temperatures measured by UV spectroscopy.

**Figure 6:**
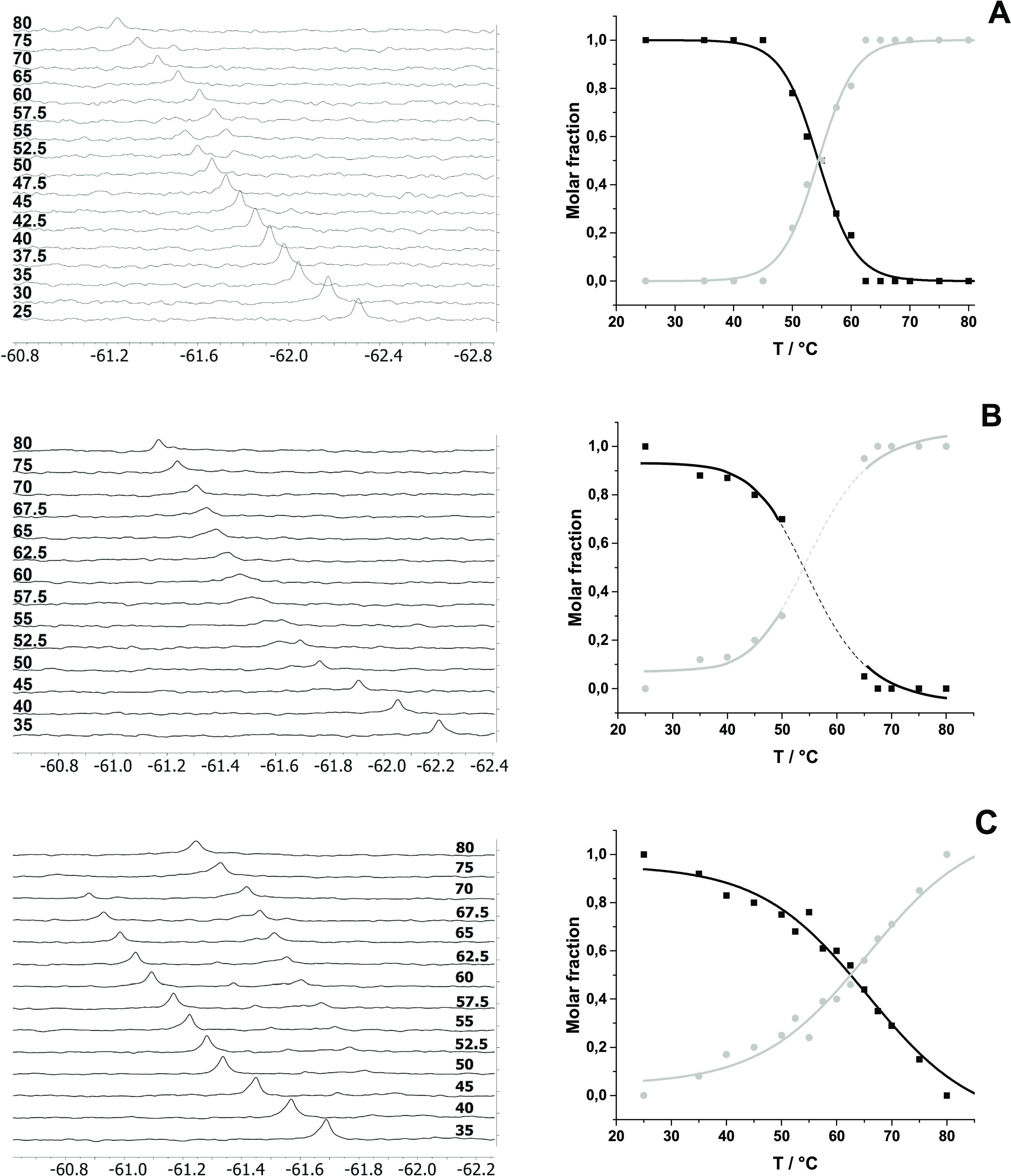
Temperature ramps and molar fractions of duplexes (A) **ON(2)·ON(C)**, (B) **ON(2)·ON(G)**, and (C) **ON(2)·ON(T)** as a function of temperature. Black lines represent the duplex, and gray lines represent **ON(2)**. Sample composition: 10 μM **ON(2)·ON(C/G/T)** in 10 mM sodium cacodylate, pH = 7.0, D_2_O–H_2_O, 1:9 (v/v), and *I*(NaCl) = 0.1 M.

Mercurated duplexes **ON(2)·ON(T)**, **ON(2)·ON(G)**, and **ON(2)·ON(C)** behaved similarly when the temperature was increased (Figure 6). The initial signals of the duplexes gradually disappeared, and a new broad signal appeared corresponding to single-stranded **ON(2)**. *T*_m_ values of **ON(2)·ON(T)** (65.5 °C) and **ON(2)·ON(C)** (54.4 °C) were extracted from the relative areas of the ^19^F NMR signals. The ^19^F NMR-based melting temperatures were close to those obtained from the UV measurements. This suggests that the dissociation of the metal-mediated base pair is coincidental with the global dissociation of the Watson–Crick base pairs. Due to the multiple signals of **ON(2)·ON(A)**, the spectra were not clear enough to allow the determination of the melting temperature (Figure S17). The ^19^F NMR-based melting profile of **ON(2)·ON(G)**, in turn, was interfered by the overlapping signals of **ON(2)** (note the downfield sigmoidal shift vs temperature curve) and **ON(2)·ON(G)** near the melting temperature, and we could not extract a clear ^19^F NMR-based *T*_m_ value for **ON(2)·ON(G)**.

Secondary structures of the mercurated duplexes were studied by CD spectropolarimetry. The same samples were used for the UV spectroscopy and ^19^F NMR measurements. Spectra were recorded between 210 and 320 nm at 10 °C intervals over a temperature range of 10–90 °C. For the NMR samples, spectra were recorded at 2 °C intervals over the same temperature range. For all duplexes, spectra consistent with typical B-type helices were observed, with the minima at λ = 250 nm and the maxima at λ = 280 nm. Increased temperature broadened these signals, which represents the unwinding of the double helices. Mercuration did not have a major effect on the average structure of the double helices, which can be seen from the CD spectra (Figures S29–S36).

## Conclusions

In this paper, we have reported a new trifluoromethyl-containing organomercury nucleobase analogue (**2**) which is able to form stable metal-mediated base pairs with guanine and thymine. Based on melting temperature analysis, the probe is capable of discriminating purine and pyrimidine bases in the middle of the sequence. The affinity followed the order of T > G > C > A. **2** proved to be a sensitive ^19^F NMR label for the detection of local environmental changes of the Hg(II)-mediated base pairs in micromolar concentrations. Valuable information of the Hg(II)-mediated base pairs could be obtained. The ^19^F NMR shift of the mercurated single strand [**ON(2)**] responded slightly to changes in temperature, which may indicate an intramolecular Hg(II)-mediated binding within the sequence. Distinct and well-resolved ^19^F NMR resonance signals were detected in double helices for **2**-T, **2**-C, and **2**-G base pairs, but binding of **2** to adenine proved obscure. The melting temperatures of **2**-T and **2-**C base pairs could be extracted from the relative peak areas of ^19^F NMR signals. These local melting temperatures proved to be in conjunction with the global melting temperatures extracted from the UV melting profiles.

## Experimental Section

### General Methods

NMR spectra were recorded on Bruker Avance 500 and 600 MHz instruments. Mass spectra were recorded on a Bruker microQTOF ESI mass spectrometer, CD spectra were recorded on an Applied Photophysics Chirascan spectropolarimeter, and UV spectra were recorded on a PerkinElmer Lambda 35 UV/vis spectrometer. Oligonucleotides were synthesized by an Applied Biosystems 3400 DNA/RNA synthesizer. Freshly distilled triethylamine was used to prepare the HPLC buffers. Solvents were dried using 3 Å or 4 Å molecular sieves. Triethylamine was dried over CaH_2_. The other reagents were commercial products and used as such.

### Oligonucleotide Synthesis

ON(A,C,G,T) were commercial products, and other ONs were synthesized using standard protocols except for a prolonged coupling time for **1** and the use of phenoxyacetic anhydride for capping to prevent the acetylation of the amino group of **1**. Oligonucleotides were released from the solid support by standard ammonolysis. Crude oligonucleotides were purified by RP-HPLC [HPLC conditions: Clarity Oligo-RP C18 column (250 × 10 mm, 10 μm); flow rate, 3 mL/min; L = 260 nm; buffer A, 0.1 M triethylammonium acetate in water; buffer B, 0.1 M triethylammonium acetate in acetonitrile; and gradient: 0.0 min 95% A, 5% B and 25.0 min 65% A, 35% B]. For the mercuration of ONs, 30 equiv of Hg(OAc)_2_ was added to a 100 μM ON solution in an aqueous 5 mM NaOAc solution (500 μL). The mixture was incubated at 55 °C for 24 h. A quenching solution containing 0.1 M EDTA in an aqueous 0.01 M Tris–HCl solution (500 μL) was added, and the mixture was purified by RP-HPLC [HPLC conditions: Hypersil ODS C18 column (250 × 4.6 mm, 5 μm); flow rate = 1 mL/min; L = 260 nm; buffer A, 0.1 M triethylammonium acetate in water; buffer B, 0.1 M triethylammonium acetate in acetonitrile; and gradient: 0.0 min 95% A, 5% B and 25.0 min 65% A, 35% B].

Mercurated oligonucleotides were designed to mercurate only in the sixth position of the nucleobase analogue by treatment with mercury(II) acetate. The selectivity of mercuration was confirmed by enzymatic digestion with P1 nuclease and mass spectrometric analysis of the fragments. 1 nmol **ON(2)** was dissolved in 25 mM TEAA buffer (100 μL, pH = 7.0). 5 μL of a solution of the P1 nuclease enzyme (0.2 μg) was added, and the mixture was incubated at 37 °C for 6 h. From the mass spectrum, all nucleoside monophosphates or nucleosides were identified, as well as a dimer of phosphorylated 5-methylcytosine and the mercurated fluoroprobe.

### ^19^F NMR Measurements

Samples of 10 μM **ON(2)** or 5 μM **ON(1)** in 10 mM sodium cacodylate (pH = 7.0, *I* = 0.1 M (NaCl), 1:9 D_2_O/H_2_O, v/v) were prepared and titrated using 0.5, 1, and 2 equiv of complementary strands [**ON(Y)**]. All samples were heated to 90 °C in a water bath and allowed to cool to room temperature (r.t) before measurements. Typical experiment parameters are as follows: the number of scans of 2048, an excitation time of 11 μs, an acquisition time of 0.97 s, a prescan delay of 18 μs, and a relaxation delay of 1.0 s. Elevated-temperature measurements were carried out with 5 or 10 μM samples over a temperature range of 25–80 °C at 2.5 or 5 °C intervals.

### Melting Temperature Measurements

Measurements were carried out using samples of 2 μM **ON(2)** or **ON(1)** and 1 equiv of **ON(Y)** in 10 mM sodium cacodylate [pH = 7.0, *I* = 0.1 M (NaCl)]. UV melting profiles were measured by monitoring the absorbance at 260 nm over a temperature range of 10–80 °C at a rate of 0.5 °C/min. *T*_m_ values were determined as the maxima of the first derivates of the melting curves.

### CD Spectropolarimetric Measurements

NMR and UV samples were used for CD measurements. Spectra were recorded between 210 and 320 nm over a temperature range 10–90 °C at 2 °C intervals with mercurated samples and at 5 °C intervals with reference samples. Samples were allowed to equilibrate for 4 min at each temperature before measuring.

#### 4-Iodo-2-trifluoromethyl-*N*-trifluoroacetylaniline (**5**)

4-Iodo-2-trifluoromethylaniline (1.28 g, 4.46 mmol) was dissolved in dry dichloromethane. Trifluoromethanesulfonic anhydride (1.30 mL, 9.20 mmol) was added, and the mixture was stirred overnight at r.t. The mixture was diluted with dichloromethane, washed with saturated Na_2_CO_3_, dried with Na_2_SO_4_, and evaporated to dryness under vacuum. The product was obtained as a white crystalline solid (yield: 1.60 g, 94%). ^1^H NMR (500 MHz, CDCl_3_): δ = 8.17 (s, 1H, NH), 8.01 (s, 1H, H3), 7.96 (d, *J* = 8.8 Hz, 1H, H5), 7.92 (d, *J* = 8.7 Hz, 1H, H6). ^13^C{^1^H} NMR (125 MHz, CDCl_3_): δ = 155.1 (q, *J* = 38.0 Hz), 142.3, 135.4 (q, *J* = 5.5 Hz), 132.1, 126.1, 123.0 (q, *J* = 30.8 Hz), 122.4 (q, *J* = 273 Hz), 115.5 (q, *J* = 290 Hz), 90.3. ^19^F NMR (470 MHz, CDCl_3_): δ = −60.7, −76.1. HRMS (ESI) *m*/*z*: calcd for C_9_H_3_F_6_INO^–^, 381.9169; observed, [M – H]^−^, 381.9155.

#### 4-[3-*O*-(*tert*-Butyldimethylsilyl)-2-deoxy-2,3-didehydro-β-d-*erythro*-pentofuranosyl]-2-trifluoromethyl-*N*-trifluoroacetylaniline (**6**)

{(2*R*,3*S*)-3-[(*tert*-Butyldimethylsilyl)oxy]-2,3-dihydrofuran-2-yl}methanol (**4**) was prepared as previously published.^50^**4** (0.819 g, 3.56 mmol) and 4-iodo-2-trifluoromethyl-*N*-trifluoroacetylaniline (**5**) (1.36 g, 3.56 mmol) were dissolved in dry 1,4-dioxane (12 mL) in a three-neck flask. Pd((*t*-Bu)_2_P)_2_ (0.24 g, 0.47 mmol) and Cy_2_NMe (0.84 mL, 4.06 mmol, 1,1 equiv) were added, and the resulting mixture was stirred at 70 °C in an oil bath for 44 h under an argon atmosphere. The reaction mixture was cooled down and evaporated to dryness. The crude product was purified with silica gel column chromatography eluting with 15–30% ethyl acetate in hexane. The product was obtained as a yellowish oil (yield: 0.464 g, 27%). TLC (15% EA/Hex) ^1^H NMR (500 MHz, CDCl_3_): δ = 8.21 (s, 1H), 8.10 (d, 1H, *J* = 8.5 Hz), 7.78 (d, 1H, *J* = 1.6 Hz), 7.68 (dd, 1H, *J* = 8.5 Hz & 1.6 Hz), 5.77 (dd, 1H, *J* = 3.9 & 1.4), 4.83 (s, 1H), 4.65 (m, 1H) 3.77 (m, 2H), 1.66 (s, 1H), 0.96 (s, 9H), 0.25 (s, 6H). ^13^C{^1^H} NMR (125 MHz, CDCl_3_): δ = 155.2 (q, *J* = 37.9 Hz), 152.3, 141.8, 131.9, 131.7, 125.6 (q, *J* = 5.0 Hz), 124.9, 123.6 (q, *J* = 272.1 Hz), 121.9 (q, *J* = 30.6) 115.6 (q, *J* = 288.2 Hz), 100.7, 83.8, 83.7, 63.1, 25.6, 18.2, −4.8. ^19^F NMR (470 MHz, CDCl_3_): δ = −60.6, −76.1. HRMS (ESI) *m*/*z*: calcd for C_20_H_27_F_4_NO_4_SiNa^+^, 508.1349; observed, 508.1338 [M + Na]^+^.

#### 4-[(2*R*,5*R*)-5-(Hydroxymethyl)-4-oxotetrahydrafuran-2-yl]-2-trifluoromethyl-*N*-trifluoroacetylaniline (**7**)

Compound **6** (0.464 g, 0.956 mmol) was dissolved in dry tetrahydrofuran (THF) at 0 °C under an argon atmosphere. Et_3_N·3HF (0.8 mL, 4.79 mmol) was added, and the reaction mixture was stirred for 20 min at r.t. The product mixture was filtered through a short silica plug (90% EA/Hex) and afterward purified with silica gel column chromatography eluting with 70% ethyl acetate in hexane. The product was obtained as a yellowish oil (yield: 0.205 g, 58%). ^1^H NMR (500 MHz CDCl_3_): δ = 8.24 (s, 1H), 8.21 (d, 1H, *J* = 8.5 Hz), 7.82 (s, 1H), 7.34 (d, 1H, *J* = 8.6 Hz), 5.30 (dd, 1H, *J* = 11.0 Hz & 6.0 Hz), 4.11 (t, 1H, *J* = 3.4 Hz), 4.00 (m, 2H), 2.97 (dd, 1H, *J* = 18.0 & 6.0 Hz), 2.50 (dd, 1H, *J* = 18.0 & 10.9 Hz), 2.00 (s, 1H). ^13^C{^1^H} NMR (125 MHz, CDCl_3_): δ = 212.4, 155.3 (q, *J* = 38.2 Hz), 139.1, 132.2, 130.9, 125.1, 124.4 (q, *J* = 5.4 Hz), 123.3 (q, *J* = 273.3 Hz), 122.0 (q, *J* = 30.2 Hz), 115.5 (q, *J* = 289.0 Hz), 82.4, 76.3, 61.5, 45.2. HRMS (ESI) *m*/*z*: calcd for C_14_H_11_F_6_NO_4_Na^+^, 394.0484; observed, 394.0471 [M + Na]^+^.

#### 4-(2-Deoxy-β-d-*erythro*-pentofuranosyl)-2-trifluoromethyl-*N*-trifluoroacetylaniline (**8**)

Compound **7** (0.205 g, 0.552 mmol) and NaBH(OAc)_3_ (0.372 g, 1.75 mmol) were dissolved in a mixture of acetonitrile and acetic acid (4 mL 1:1, v/v). The reaction mixture was stirred for 10 min, after which a mixture of water and ethanol (2 mL 1:1, v/v) was added to quench the reaction. The product mixture was evaporated to dryness in vacuum and purified by silica gel column chromatography eluting with 10% MeOH/CH_2_Cl_2_. The product was obtained as a yellowish oil (yield: 0.165 g, 80%). ^1^H NMR (500 MHz CD_3_CN): δ = 7.85 (s, 1H), 7.74 (d, 1H, *J* = 8.3 Hz), 7.53 (d, 1H, *J* = 8.2 Hz), 5.21 (dd, 1H, *J* = 10.4 Hz & 5.5 Hz), 4.32 (m, 1H), 3.95 (m, 1H), 3.65 (d, *J* = 5.0 Hz, 2H), 2.28 (ddd, 1H, *J* = 13.0, 5.6 & 1.6 Hz), 1.89 (ddd, 1H, *J* = 13.0, 10.4 & 5.8 Hz). ^13^C{^1^H} NMR (125 MHz, CD_3_CN): δ = 156.7 (q, *J* = 37.6 Hz), 144.1, 130.8, 130.7, 129.9, 126.3 (q, *J* = 30.2 Hz), 124.5, 123.4 (q, *J* = 273.2 Hz), 116.0(q, *J* = 287.3 Hz), 88.1, 78.8, 73.1, 62.8, 43.7. ^19^F NMR (470 MHz, CDCl_3_): δ = −61.37, −76.41. HRMS (ESI) *m*/*z*: calcd for C_14_H_13_F_6_NO_4_Na^+^, 396.0641; observed, 396.0631 [M + H]^+^.

#### 4-[5-*O*-(4,4′-Dimethoxytrityl)-2-deoxy-β-d-*erythro*-pentofuranosyl]-2-trifluoromethyl-*N*-trifluoroacetylaniline (**9**)

Compound **8** (0.165 g, 0.442 mmol) was coevaporated twice with dry pyridine and afterward dissolved in dry pyridine (10 mL). DMTrCl (0.1638 g, 0.486 mmol) was added, and the reaction mixture was stirred for 6 h at room temperature until the completion of the reaction [monitored by thin-layer chromatography (TLC) (10% MeOH/EA)]. The mixture was concentrated, diluted with dichloromethane, and washed with sat. aq. NaHCO_3_. The organic phase was dried with Na_2_SO_4_ and evaporated to dryness in vacuum. The crude product was purified on a silica gel column eluting with 10% MeOH/EA and 1% Et_3_N. The product was obtained as a yellowish oil (yield: 0.177 g, 59%). ^1^H NMR (500 MHz CDCl_3_): δ = 8.48 (s, 1H), 7.90 (d, 1H, *J* = 8.4 Hz), 7.79 (s, 1H), 7.62 (d, 1H, *J* = 8.3 Hz), 7.48 (m, 2H), 7.37 (m, 4H), 7.39 (m, 2H), 7.22 (m, 1H), 6.84 (m, 4H), 5.23 (dd, 1H, *J* = 10.2 & 5.1 Hz), 4.44 (m, 1H), 4.15 (m, 1H) 3.78 (s, 6H), 3.34 (m, 2H), 2.30 (m, 1H), 2.01 (m, 1H). ^13^C{^1^H} NMR (125 MHz, CDCl_3_): δ = 158.5, 155.5 (q, *J* = 38 Hz) 144.8, 142.0, 136.0, 131.0, 130.6, 130.1, 128.2, 127.8, 126.8, 124.5(q, *J* = 5.2 Hz), 123.5 (q, *J* = 272 Hz), 123.0 (q, *J* = 30.0 Hz), 113.2, 86.3, 86.2, 78.9, 74.2, 64.3, 55.2, 43.7. ^19^F NMR (470 MHz, DMSO-*d*_6_): δ = −59.78, −74.21. HRMS (ESI) *m*/*z*: calcd for C_35_H_32_F_4_NO_6_Na^+^, 698.1948; observed, 698.1977 [M + Na]^+^.

#### 4-{3-*O*-[(2-Cyanoethoxy)(*N*,*N*-diisopropylamino)phosphinyl]5-*O*-(4,4′-dimethoxytrityl)-2-deoxy-β-d-*erythro*-pentofuranosyl}-2-trifluoromethyl-*N*-trifluoroacetylaniline (**3**)

Compound **9** (0.177 g, 0.262 mmol) was dissolved in dry dichloromethane (5 mL) under a nitrogen atmosphere. 2-Cyanoethyl *N*,*N*-diisopropylchlorophosphoramidite (184 μL, 0.288 mmol) and dry triethylamine (5 eq, 146 μL) were added, and the reaction mixture was stirred for 1 h at r.t. After the completion of the reaction, the product mixture was diluted with dichloromethane (50 mL) and washed with sat. aq. NaHCO_3_ (50 mL). The product was obtained as a colorless oil (yield: 0.229 g, 97%). Diastereomer mixture ^1^H NMR (500 MHz CDCl_3_): δ = 8.28 (s, 1H), 8.06 (d, 1H, *J* = 8.5 Hz), 7.83 (s, 1H), 7.68 (d, *J* = 8.4 Hz) 7.48 (m, 2H), 7.37 (m, 4H), 7.30 (m, 2H) 7.23 (m, 1H), 6.85 (m, 4H) 5.25 (m, 1H), 4.58 (m, 1H), 4.30 (m, 1H), 3.87 &3.72 (m, 2H), 3.81 (s, 6H), 3.63 (m, 2H), 3.42–3.29 (m, 2H), 2.70–2.38 (m, 3H), 2.05 (m, 1H), 1.21 (m, 12H). ^13^C{^1^H} NMR (125 MHz, CDCl_3_): δ = 158.4, 155.3 (q, *J* = 38 Hz), 144.7, 141.4, 135.9, 131.2, 130.7, 130.1, 128.2, 127.8, 126.9, 125.2, 124.1, 123.5 (q, *J* = 275.3), 122.1, 117.5, 115.7 (q, *J* = 288.6 Hz), 113.2, 86.4 & 86.1, 86.3, 79.1, 76.1, 63.9, 58.3, 55.2, 43.4, 43.2, 24.6, 20.3. ^19^F NMR (470 MHz, CDCl_3_): δ = −60.40, −76.01. ^31^P NMR (202 MHz, CDCl_3_): δ = 148.20, 148.12. HRMS (ESI) *m*/*z*: calcd for C_44_H_49_F_4_N_3_O_7_PNa^+^, 898.3026; observed, [M + Na]^+^ = 898.3054.

## Supporting Information Available

NMR, MS, and CD spectra and HPLC chromatograms (PDF)
