# Supplementary material for: 2-Trifluoromethyl-6-mercurianiline Nucleotide, a Sensitive 19F NMR Probe for Hg(II)-mediated Base Pairing
Source: J Org Chem. 2021 Dec 14;87(1):137–46. doi: 10.1021/acs.joc.1c02056 (PMC8749955; doi:10.1021/acs.joc.1c02056)
Supplement: Supplementary file 1 — jo1c02056_si_001.pdf [file jo1c02056_si_001.pdf]

Supporting Information

2-trifluoromethyl-6-mercurianiline nucleotide; a sensitive ^{19}F NMR probe for Hg(II)-mediated base pairing

MSc Asmo Aro-Heinilä[a], Ms Assi Lepistö[a], MSc Antti Äärelä[a], Dr. Tuomas Antti Lönnberg[a], Dr. Pasi Virta[a],*

[a]Department of Chemistry, University of Turku, Henrikinkatu 2, 20500 Turku (Finland)

Contents

^{1}H NMR spectrum of compound 5 (500 MHz, $CDCl_3$) S1
^{13}C{^{1}H} NMR spectrum of compound 5 (125 MHz, $CDCl_3$) S3
^{19}F NMR spectrum of compound 5 (470 MHz, $CDCl_3$) S5
^{1}H NMR spectrum of compound 6 (500 MHz, $CDCl_3$) S6
^{13}C{^{1}H} NMR spectrum of compound 6 (125 MHz, $CDCl_3$) S8
^{19}F NMR spectrum of compound 6 (470 MHz, $CDCl_3$) S10
^{1}H NMR spectrum of compound 7 (500 MHz, $CDCl_3$) S11
^{13}C{^{1}H} NMR spectrum of compound 7 (125 MHz, $CDCl_3$) S13
^{1}H NMR spectrum of compound 8 (500 MHz, CD_3CN) S15
^{13}C{^{1}H} NMR spectrum of compound 8 (125 MHz, CD_3CN) S17
^{19}F NMR spectrum of compound 8 (470 MHz, $CDCl_3$) S19
^{1}H NMR spectrum of compound 9 (500 MHz, $CDCl_3$) S20
^{13}C{^{1}H} NMR spectrum of compound 9 (125 MHz, $CDCl_3$) S22
^{19}F NMR spectrum of compound 9 (470 MHz, d_6-DMSO) S25
^{1}H NMR spectrum of compound 3 (500 MHz, $CDCl_3$) S26
^{13}C{^{1}H} NMR spectrum of compound 3 (125 MHz, $CDCl_3$) S28
^{19}F NMR spectrum of compound 3 (470 MHz, $CDCl_3$) S32
^{31}P NMR spectrum of compound 3 (202 MHz, $CDCl_3$) S33
Figure S1. RP-HPLC chromatograms of A) ON(1) crude product and B) purified ON(1). S34
Figure S2. MS spectrum of ON(1) S35
Figure S3. RP-HPLC chromatograms of ON(2) crude product with EDTA (left) and without EDTA (right). S36
Figure S4. RP-HPLC chromatograms of purified ON(2). S36
Figure S5. MS spectrum of ON(2) S37

Figure S6. ^{19}F NMR spectra of ON(1), mercuration reaction mixture and ON(2)..S38
Figure S7. MS spectrum after digestion of P1 nuclease...S39
Figure S8. UV melting profiles of ON(2)•ON(Y) (black) and ON(1)•ON(Y) (grey).......................................S41
Figure S9. ^{19}F NMR spectra of ON(1)•ON(A)...S42
Figure S10. ^{19}F NMR spectra of ON(1)•ON(C)...S43
Figure S11. ^{19}F NMR spectra of ON(1)•ON(G)...S44
Figure S12. ^{19}F NMR spectra of ON(1)•ON(T)...S45
Figure S13. ^{19}F NMR spectra of ON(2)•ON(A)...S46
Figure S14. ^{19}F NMR spectra of ON(2)•ON(C)...S47
Figure S15. ^{19}F NMR spectra of ON(2)•ON(G)...S48
Figure S16. ^{19}F NMR spectra of ON(2)•ON(T)...S49
Figure S17. ^{19}F NMR temperature ramp of ON(2)•ON(A)...S50
Figure S18. ^{19}F NMR temperature ramp of ON(2)•ON(C)...S51
Figure S19. ^{19}F NMR temperature ramp of ON(2)•ON(G)...S52
Figure S20. ^{19}F NMR temperature ramp of ON(2)•ON(T)...S53
Figure S21. ^{19}F NMR temperature ramp of ON(1)•ON(A)...S54
Figure S22. ^{19}F NMR temperature ramp of ON(1)•ON(C)...S55
Figure S23. ^{19}F NMR temperature ramp of ON(1)•ON(G)...S56
Figure S24. ^{19}F NMR temperature ramp of ON(1)•ON(T)...S57
Figure S25. ^{19}F NMR melting curves of ON(2)•ON(Y)...S58
Figure S26. Temperature dependance of ON(2) and ON(1) oligonucleotide. S58
Figure S27. ^{19}F NMR spectra comparison of ON(2) and mixed ON(2)&ON(1). S59
Figure S28. ^{19}F NMR temperature ramp of ssON(2)...S60
Figure S29. CD spectra: ON(2)•ON(A) (left) and ON(1)•ON(A) (right)..S61
Figure S30. CD spectra: ON(2)•ON(C) (left) and ON(1)•ON(C) (right). S61
Figure S31. CD spectra: ON(2)•ON(G) (left) and ON(1)•ON(G) (right). S61
Figure S32. CD spectra: ON(2)•ON(T) (left) and ON(1)•ON(T) (right)..S62
Figure S33. CD spectra of ON(2)•ON(A) (left) and ON(1)•ON(A) (right) (NMR samples) S62
Figure S34. CD spectra of ON(2)•ON(C) (left) and ON(1)•ON(C) (right) (NMR samples)...............................S62
Figure S35. CD spectra of ON(2)•ON(G) (left) and ON(1)•ON(G) (right) (NMR samples). S63
Figure S36. CD spectra of ON(2)•ON(T) (left) and ON(1)•ON(T) (right) (NMR samples). S63

^{1}H NMR spectrum of compound 5 (500 MHz,

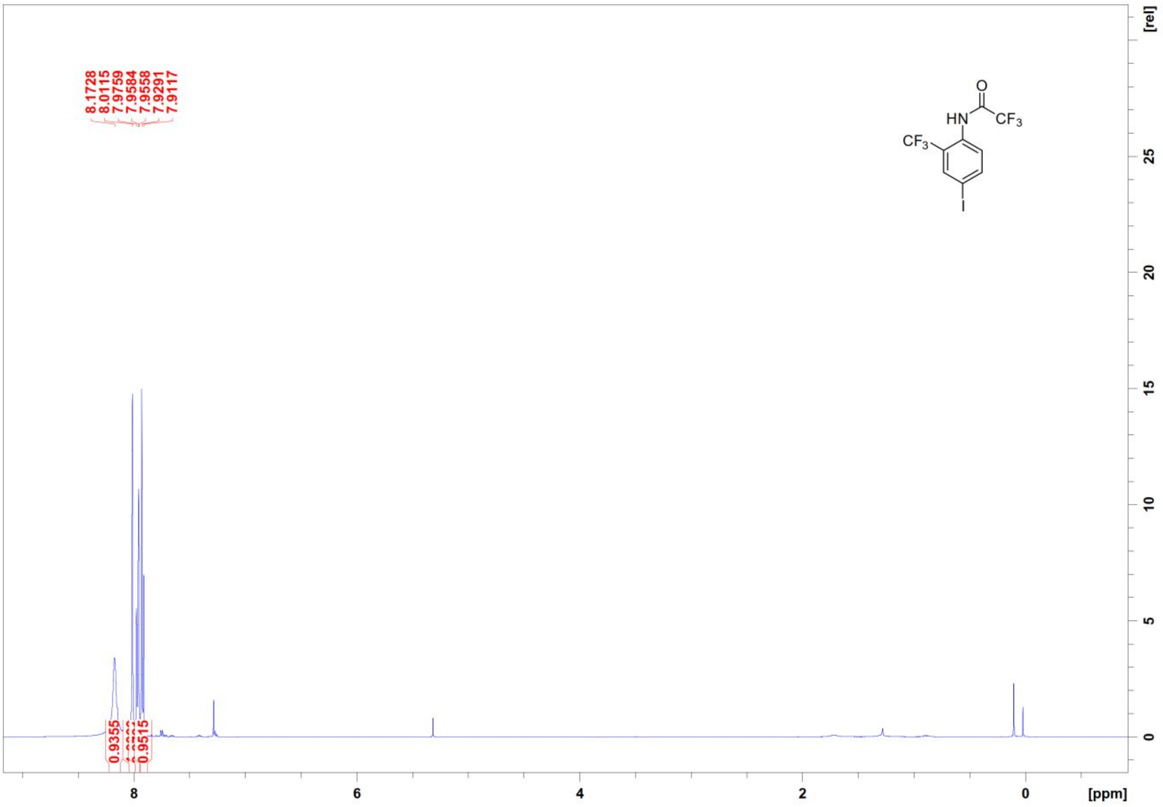

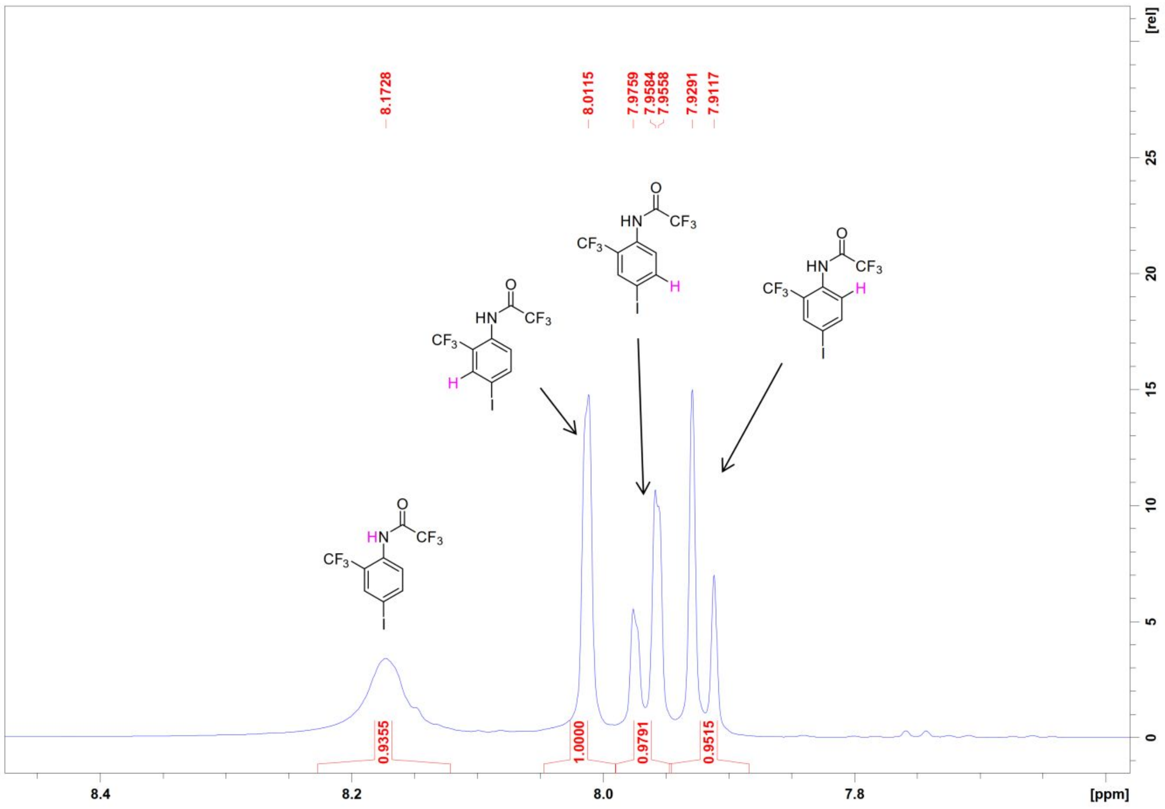

8.1728
8.0115
7.9759
7.9584
7.9558
7.9291
7.9117
0.9355
1.0000
0.9791
0.9515
[rel]
[ppm]

$^{13}C\{^{1}H\}$ NMR spectrum of compound 5 (125 MHz, $CDCl_3$)

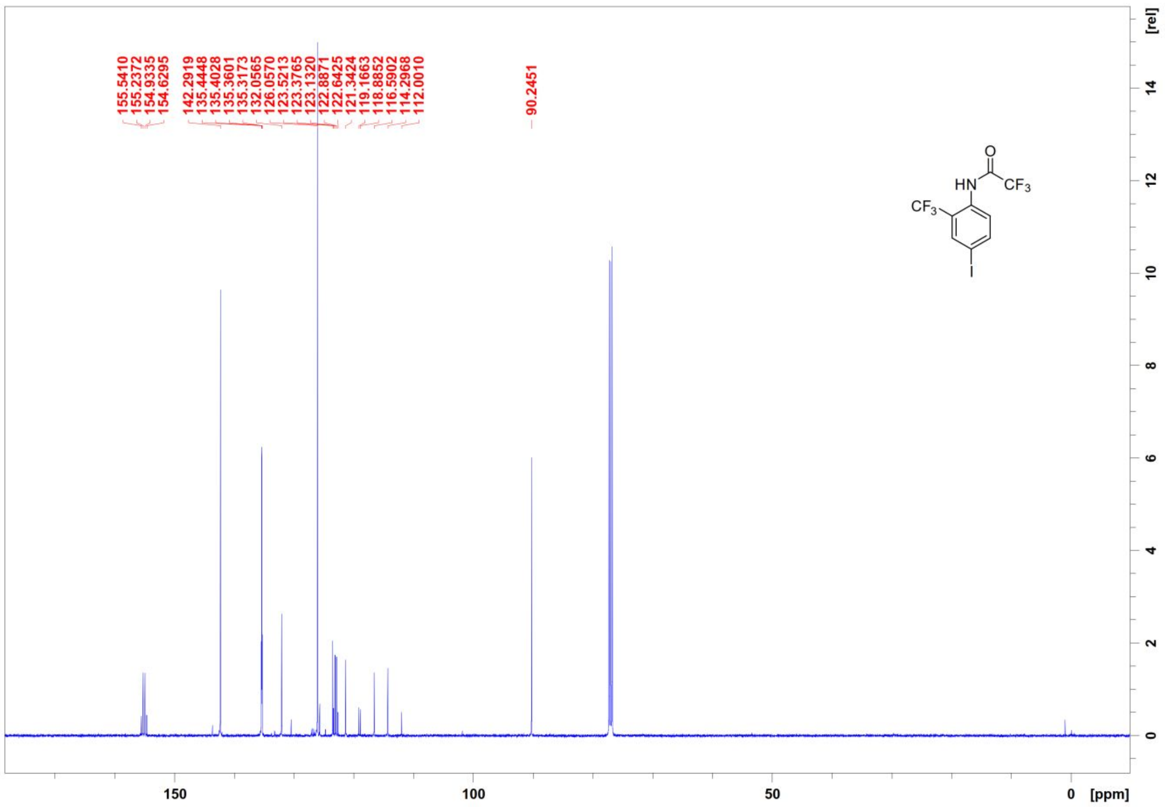

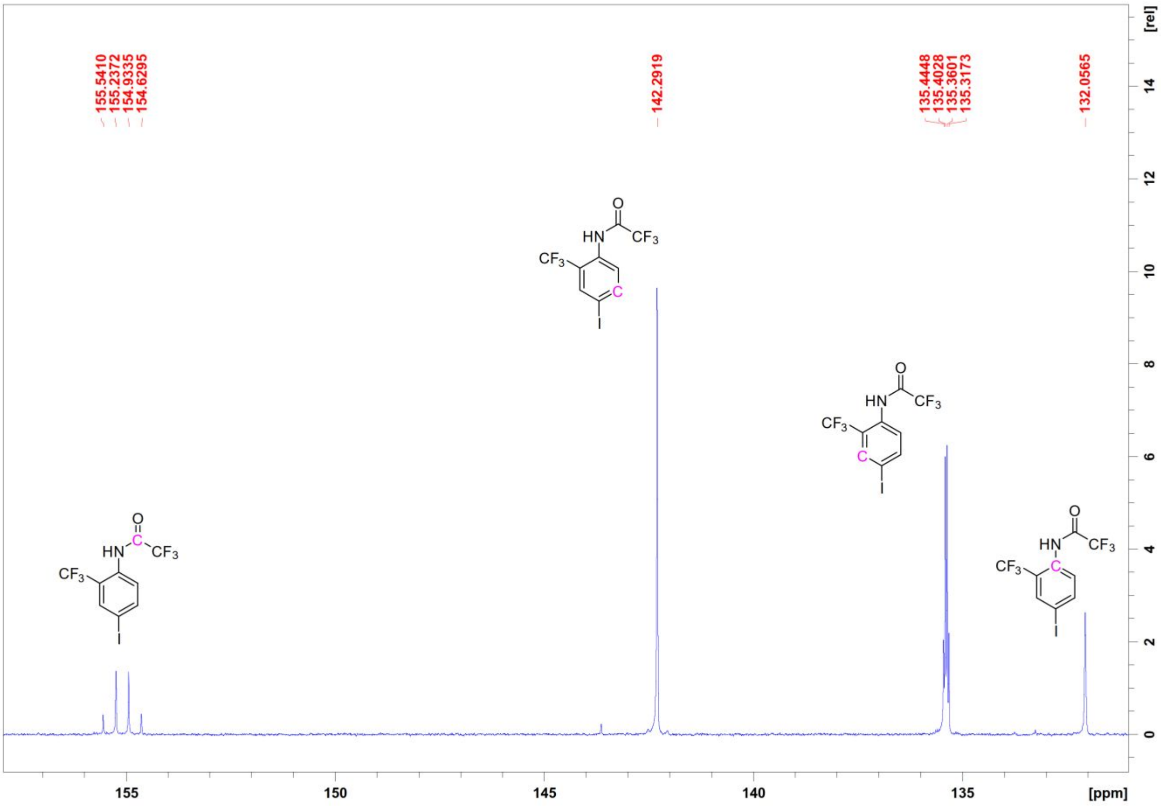
155.5410
155.2372
154.9335
154.6295
142.2919
135.4448
135.4028
135.3601
135.3173
132.0565
[rel]
[ppm]
HN
O
CF3
CF3
I
155
150
145
140
135

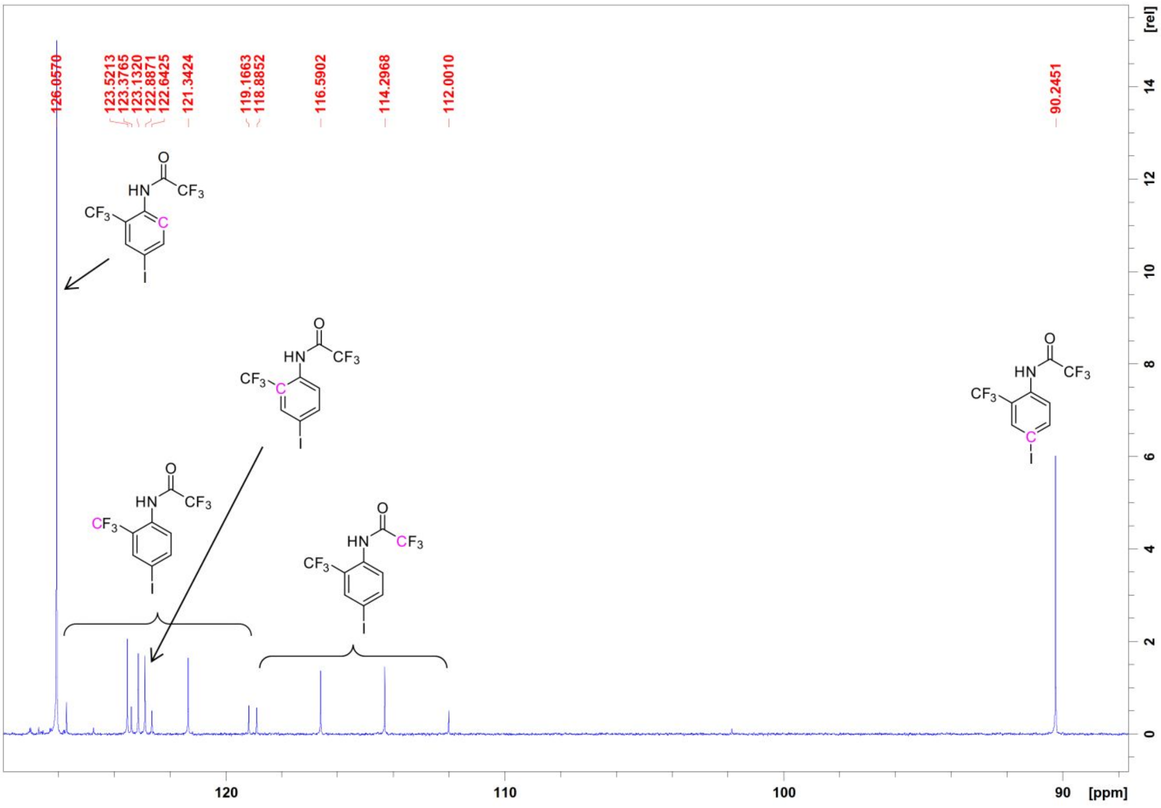
126.0570
123.5213
123.3765
123.1320
122.8871
122.6425
121.3424
119.1663
118.8852
116.5902
114.2968
112.0010
90.2451
[rel]
[ppm]
HN
O
CF3
CF3
I
120
110
100
90

^{19}F NMR spectrum of compound 5 (470 MHz, $CDCl_3$)

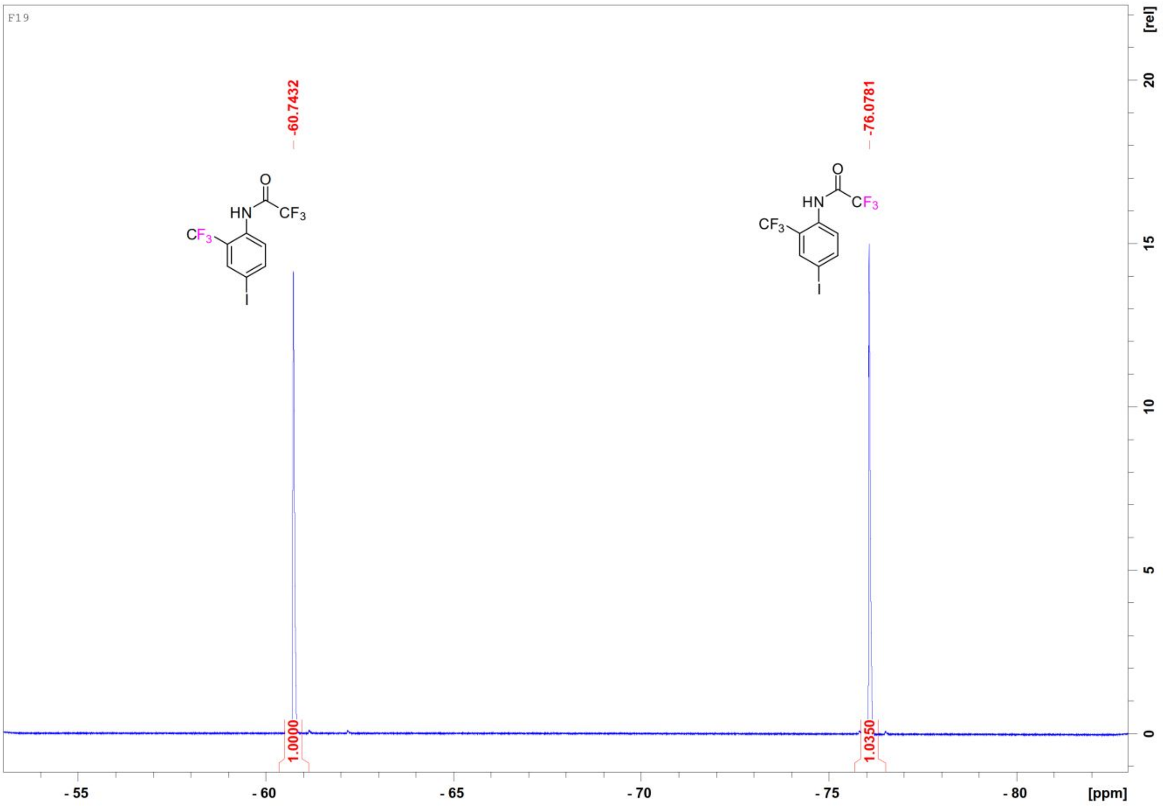

^{1}H NMR spectrum of compound 6 (500 MHz, $CDCl_3$)

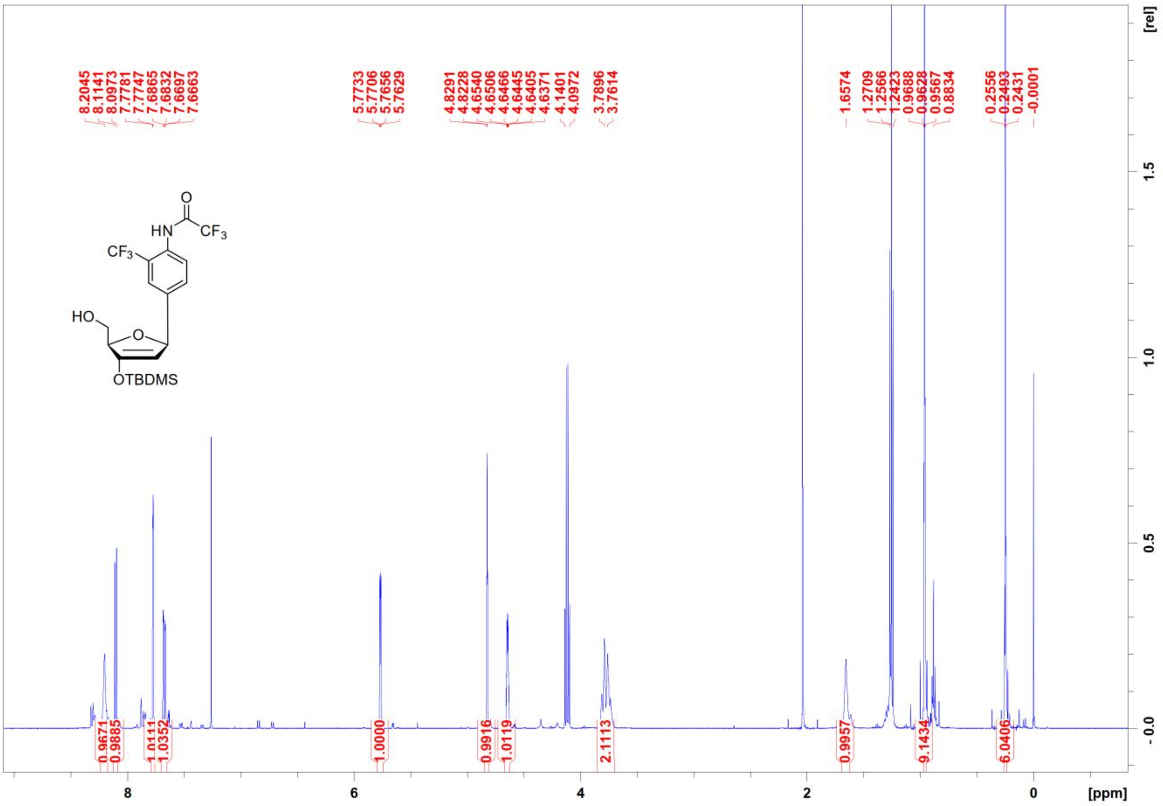

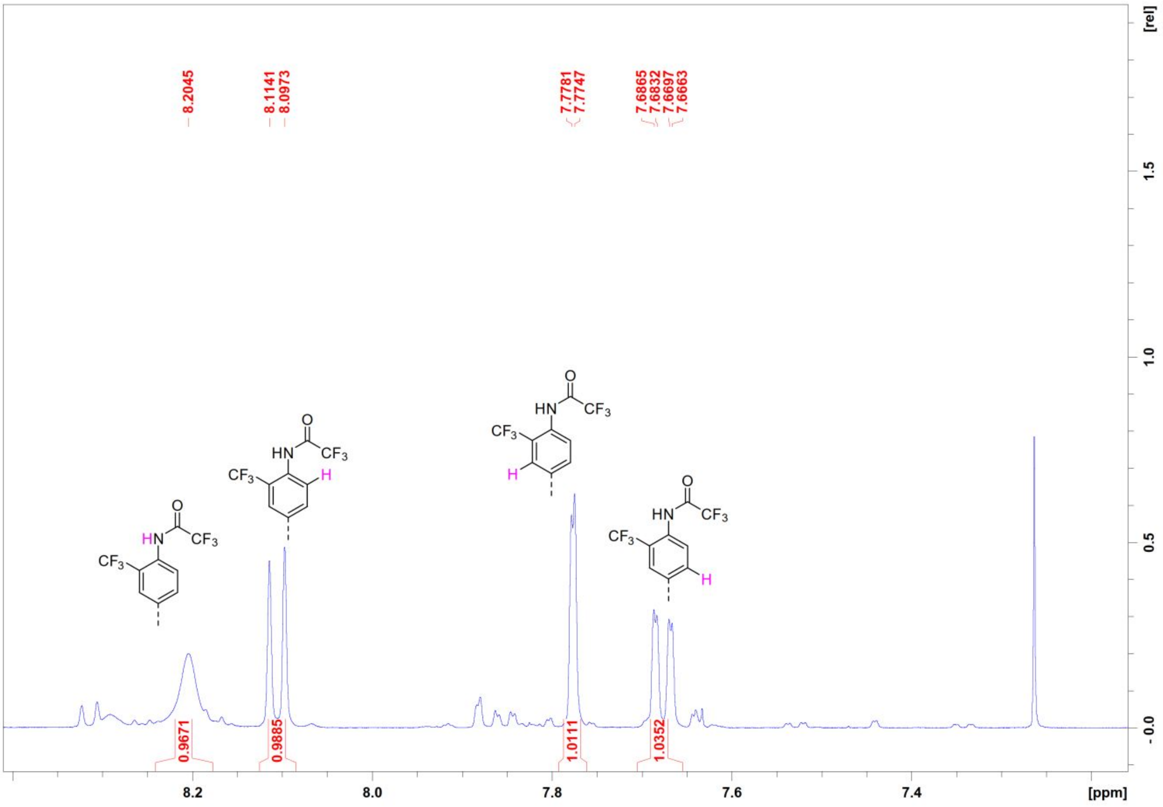

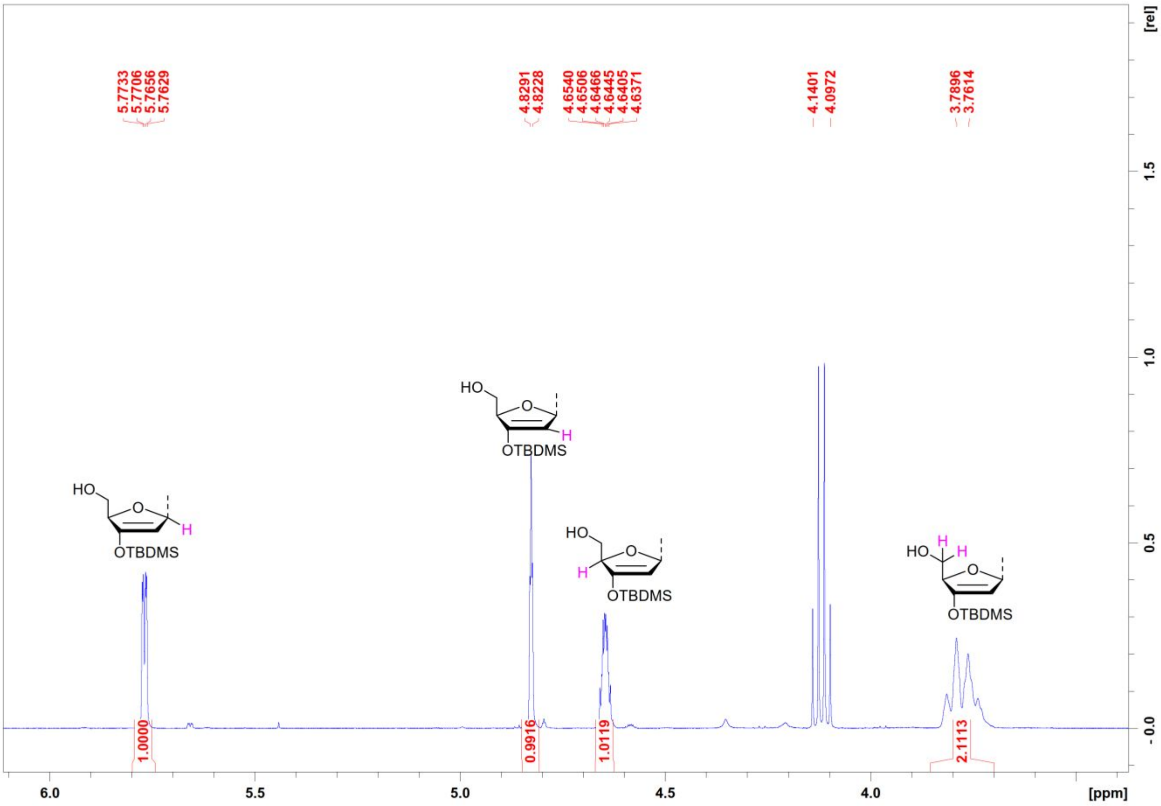
5.7733
5.7706
5.7656
5.7629
4.8291
4.8228
4.6540
4.6506
4.6466
4.6445
4.6405
4.6371
4.1401
4.0972
3.7896
3.7614
HO
O
H
OTBDMS
1.0000
0.9916
1.0119
2.1113
[rel]
[ppm]

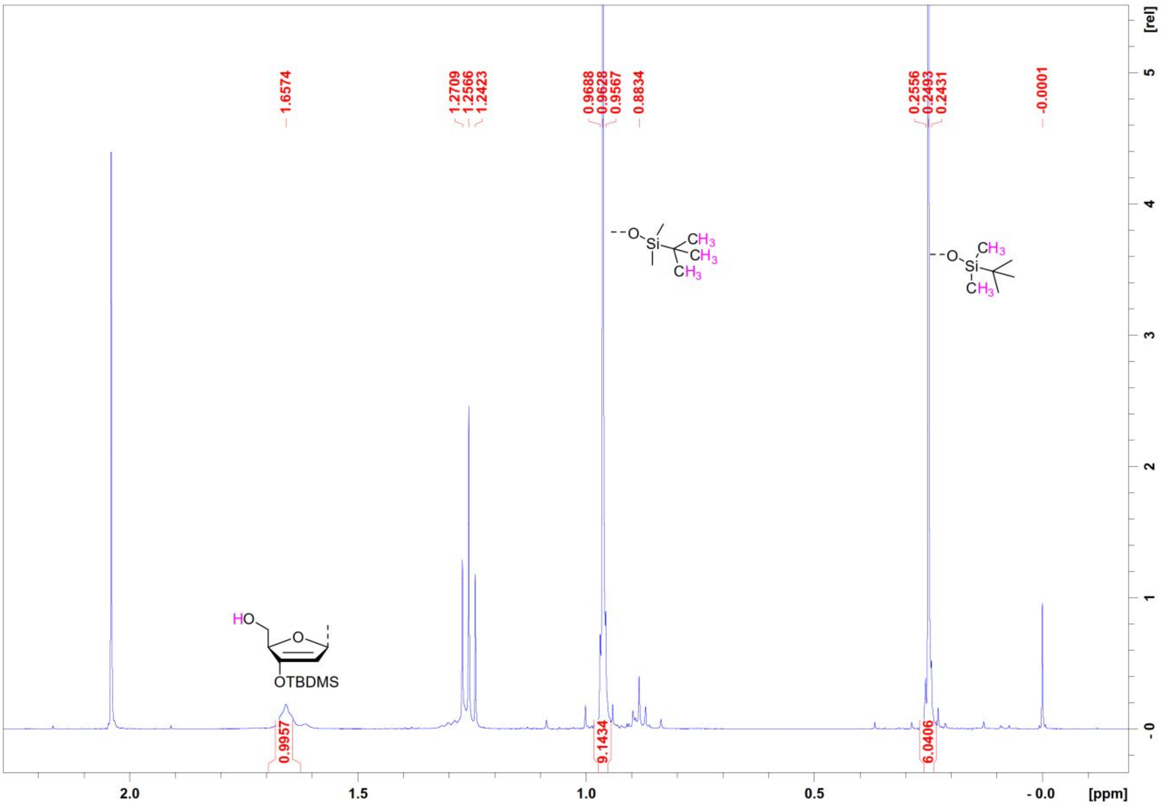
1.6574
1.2709
1.2566
1.2423
0.9688
0.9628
0.9567
0.8834
0.2556
0.2493
0.2431
-0.0001
O
Si
CH3
HO
OTBDMS
0.9957
9.1434
6.0406
[rel]
[ppm]

$^{13}C\{^1H\}$ NMR spectrum of compound 6 (125 MHz, $CDCl_3$)

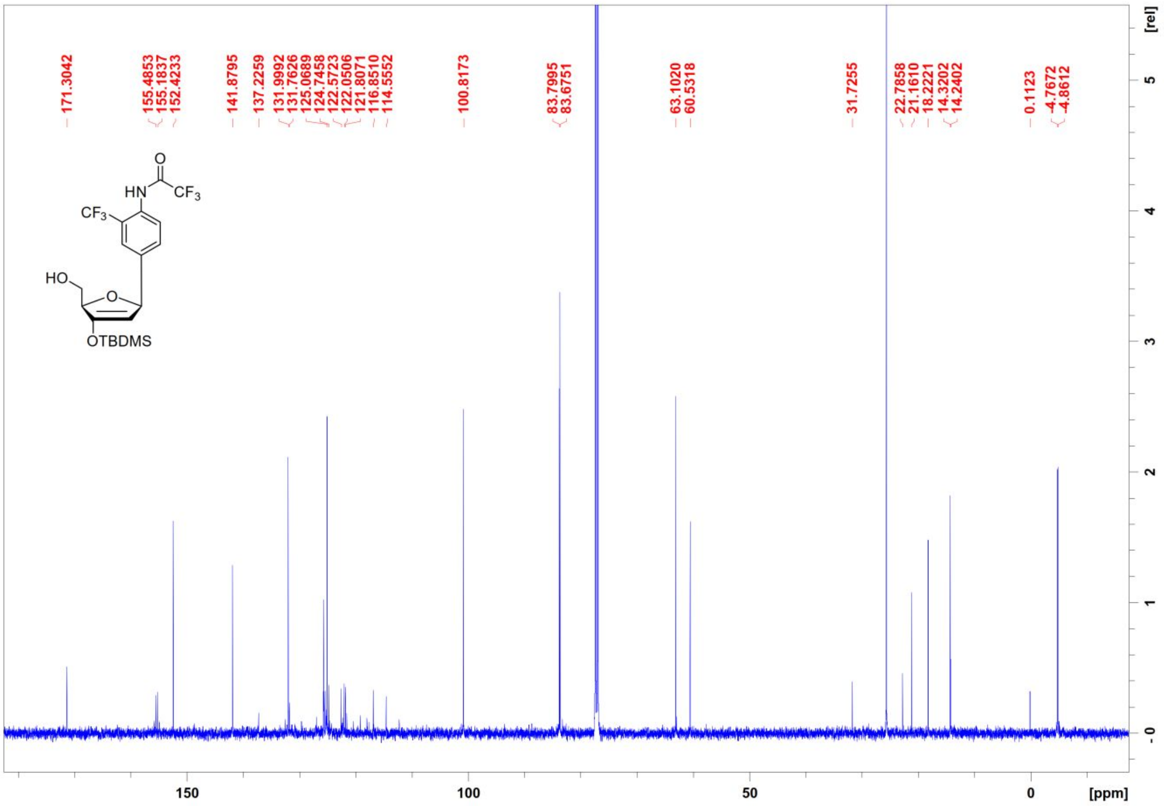

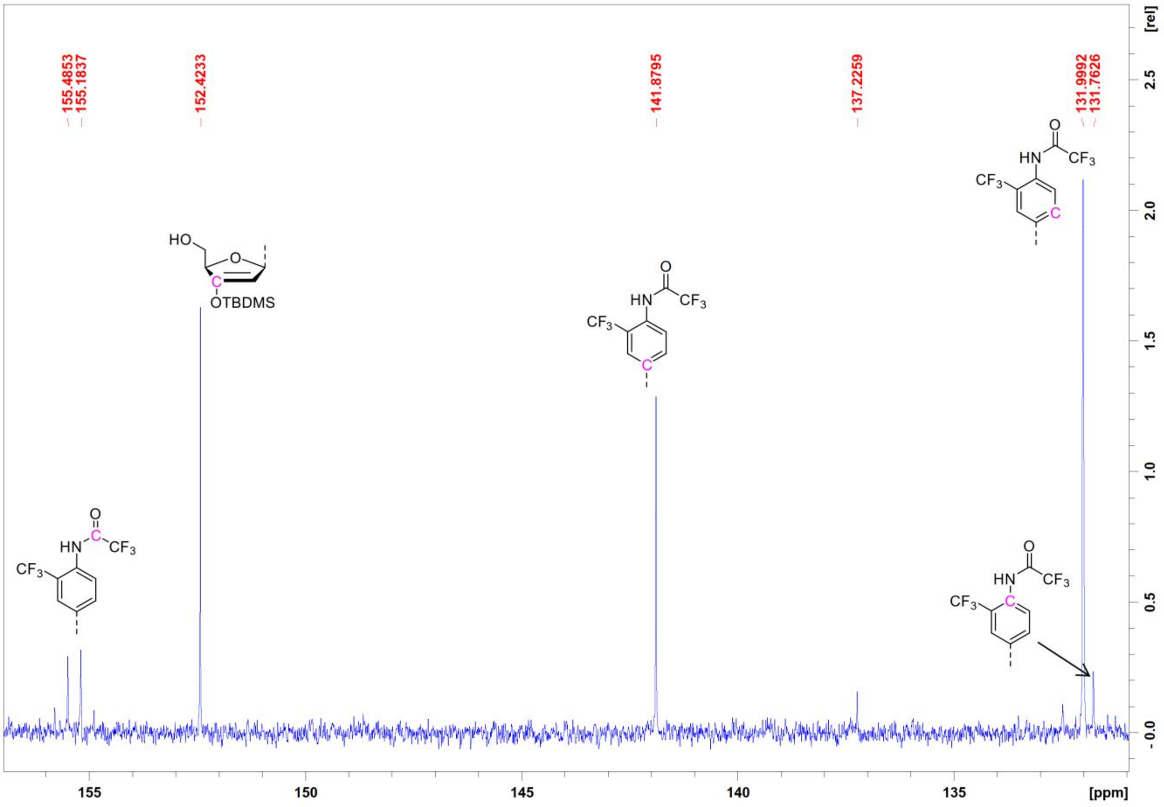

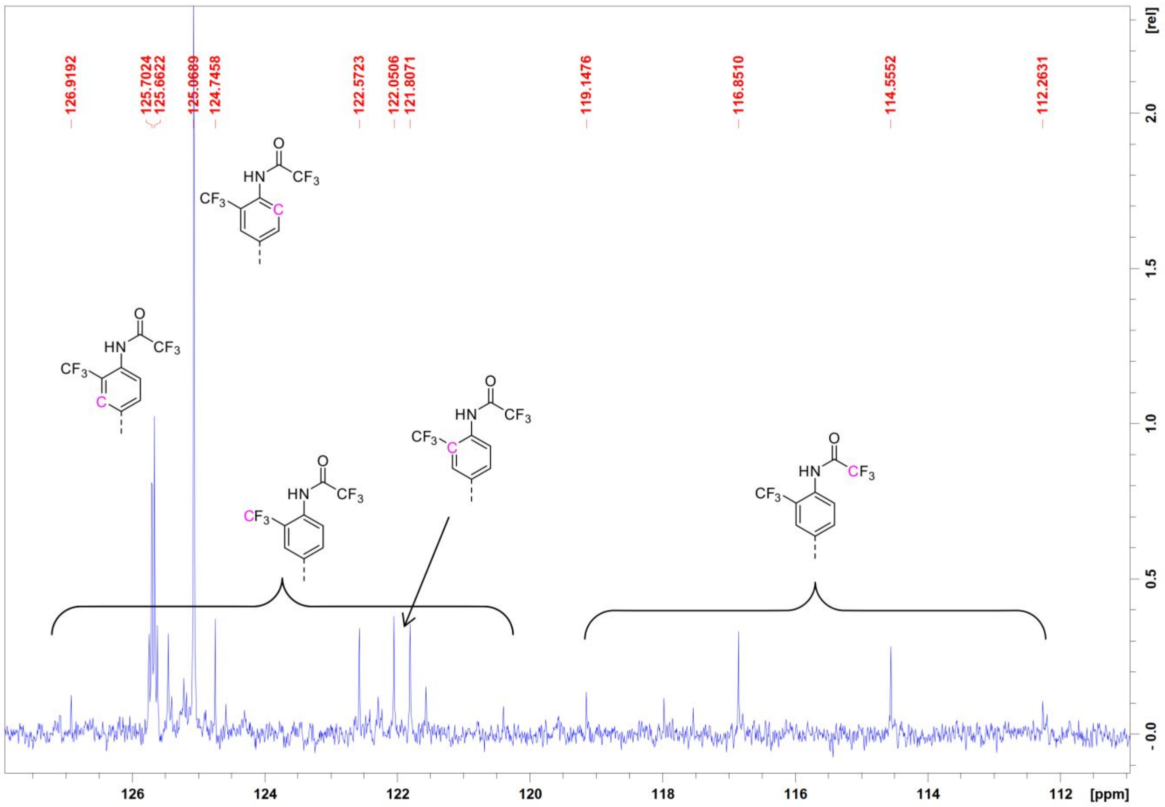

126.9192
125.7024
125.6622
125.0689
124.7458
122.5723
122.0506
121.8071
119.1476
116.8510
114.5552
112.2631
[rel]
[ppm]

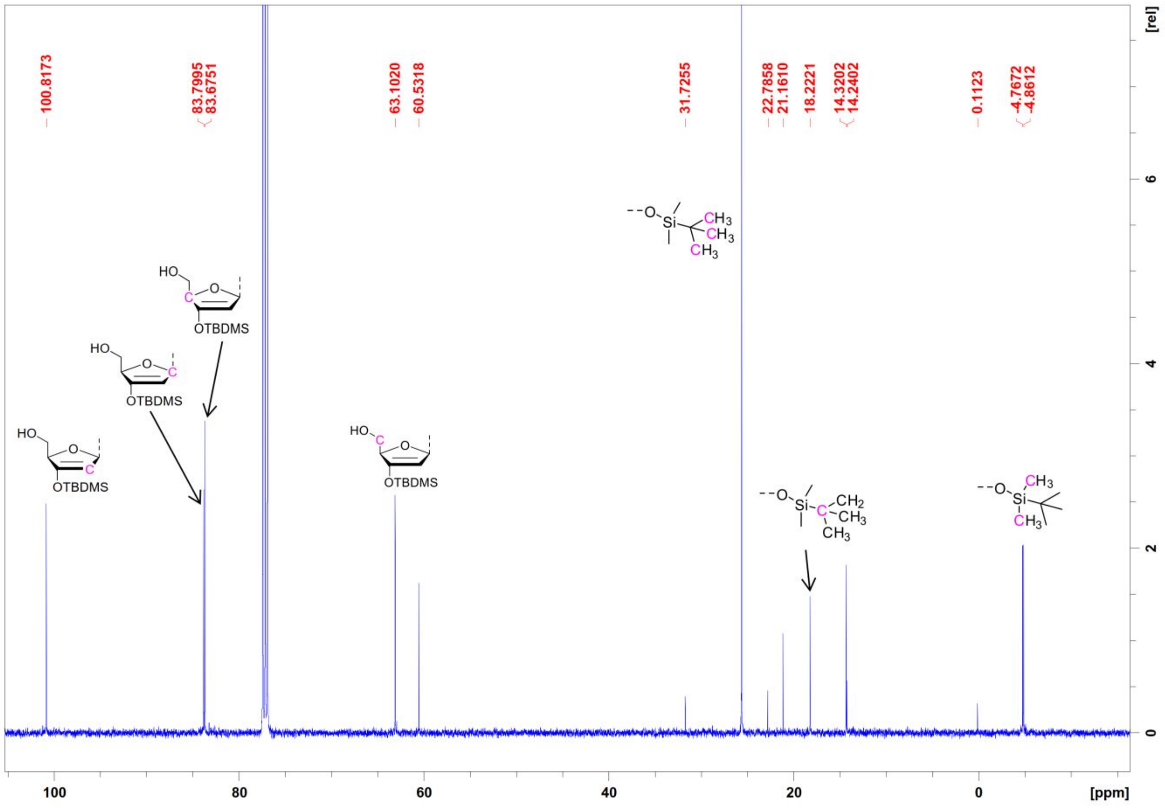

100.8173
83.7995
83.6751
63.1020
60.5318
31.7255
22.7858
21.1610
18.2221
14.3202
14.2402
0.1123
-4.7672
-4.8612
OTBDMS
[rel]
[ppm]

^{19}F NMR spectrum of compound 6 (470 MHz, $CDCl_3$)

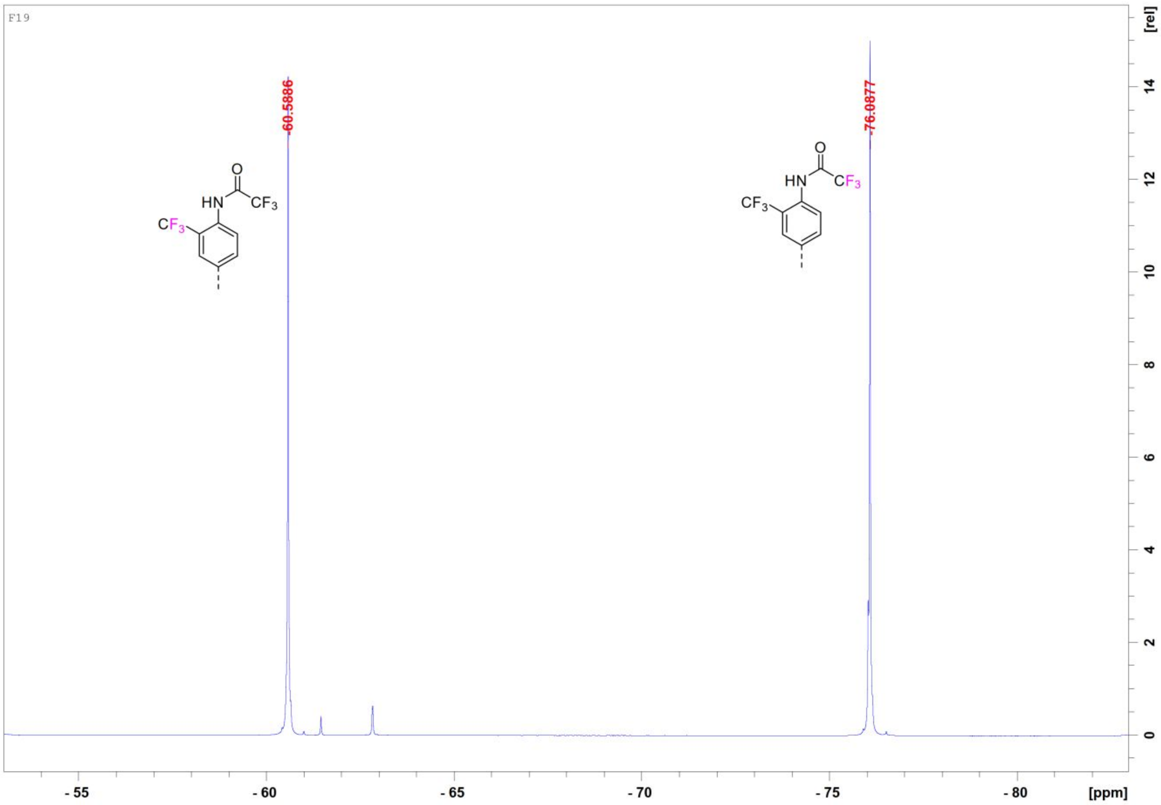

^{1}H NMR spectrum of compound 7 (500 MHz, $CDCl_3$)

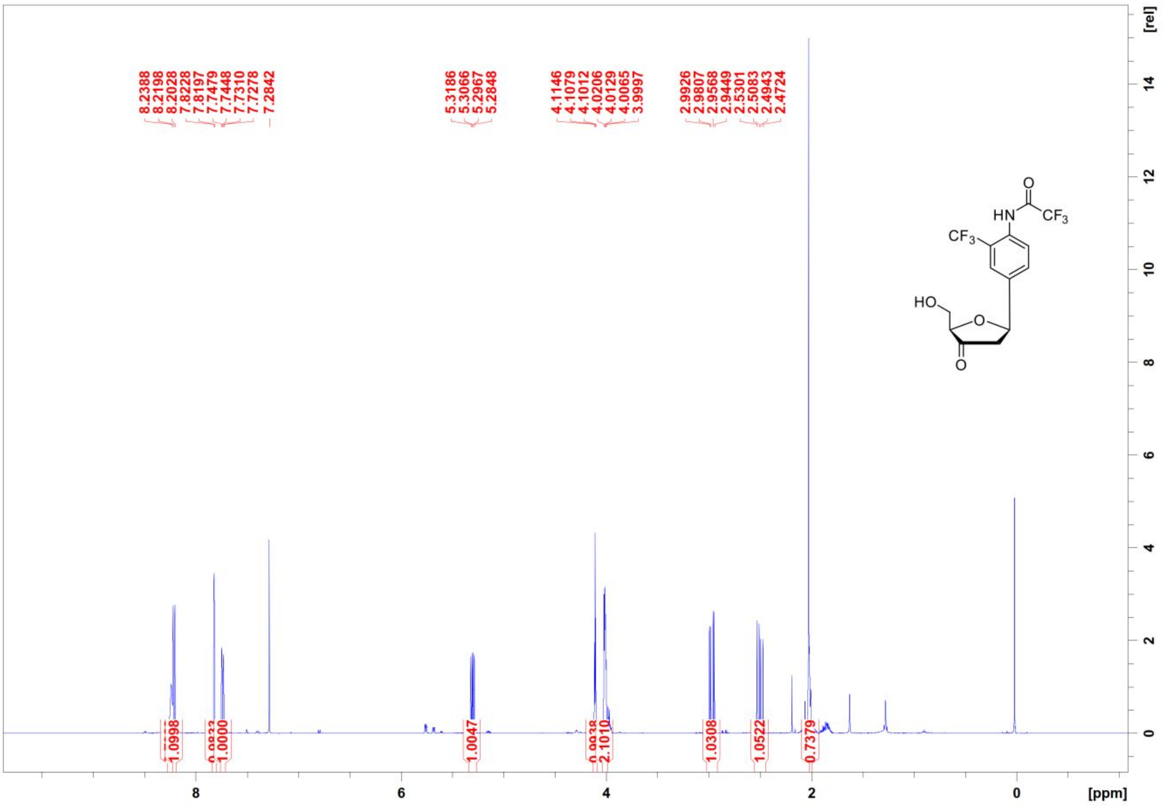

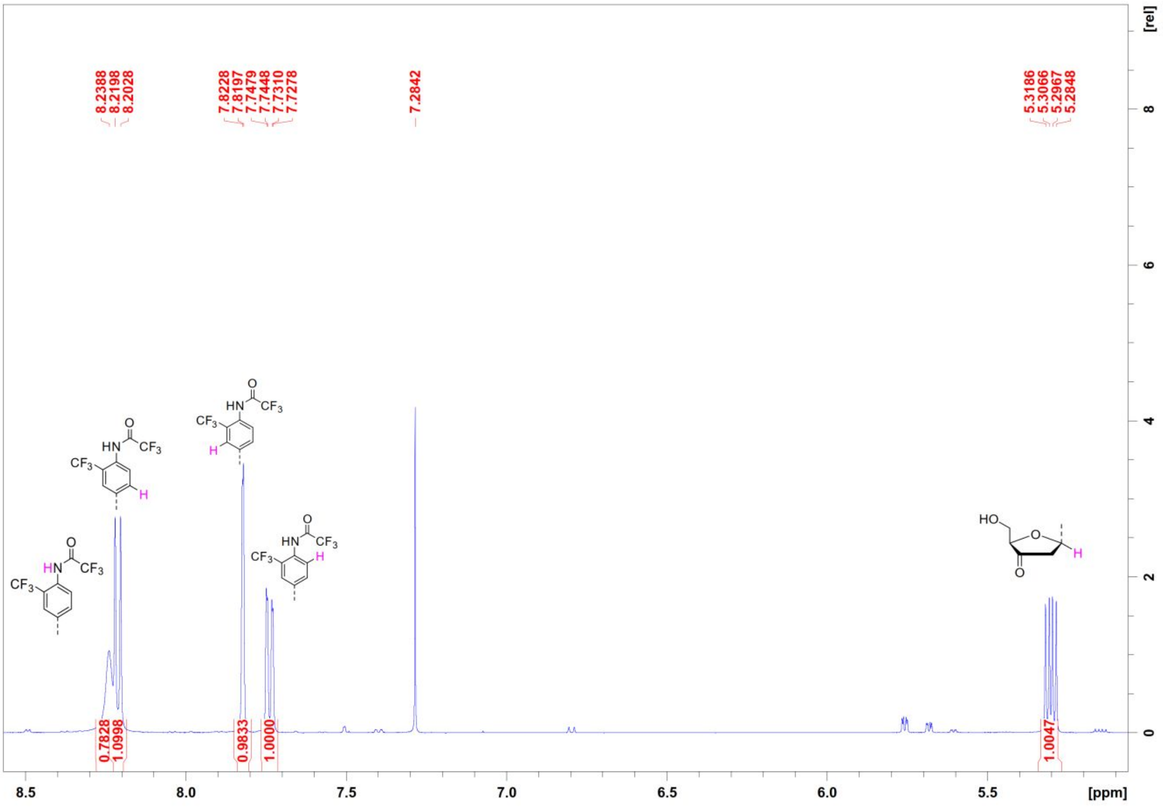

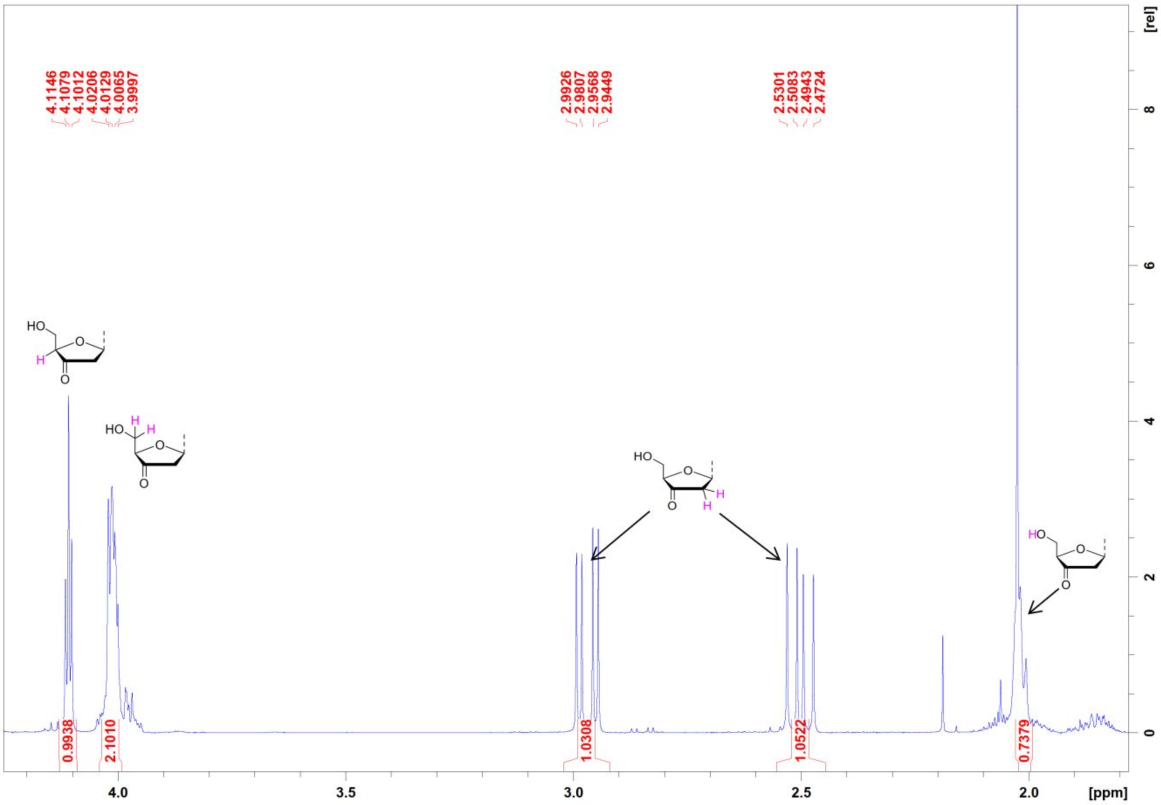
4.1146
4.1079
4.1012
4.0206
4.0129
4.0065
3.9997
2.9926
2.9807
2.9568
2.9449
2.5301
2.5083
2.4943
2.4724
HO
O
H
O
0.9938
2.1010
1.0308
1.0522
0.7379
4.0
3.5
3.0
2.5
2.0
[ppm]
[rel]
8
6
4
2
0

$^{13}C\{^1H\}$ NMR spectrum of compound 7 (125 MHz, $CDCl_3$)

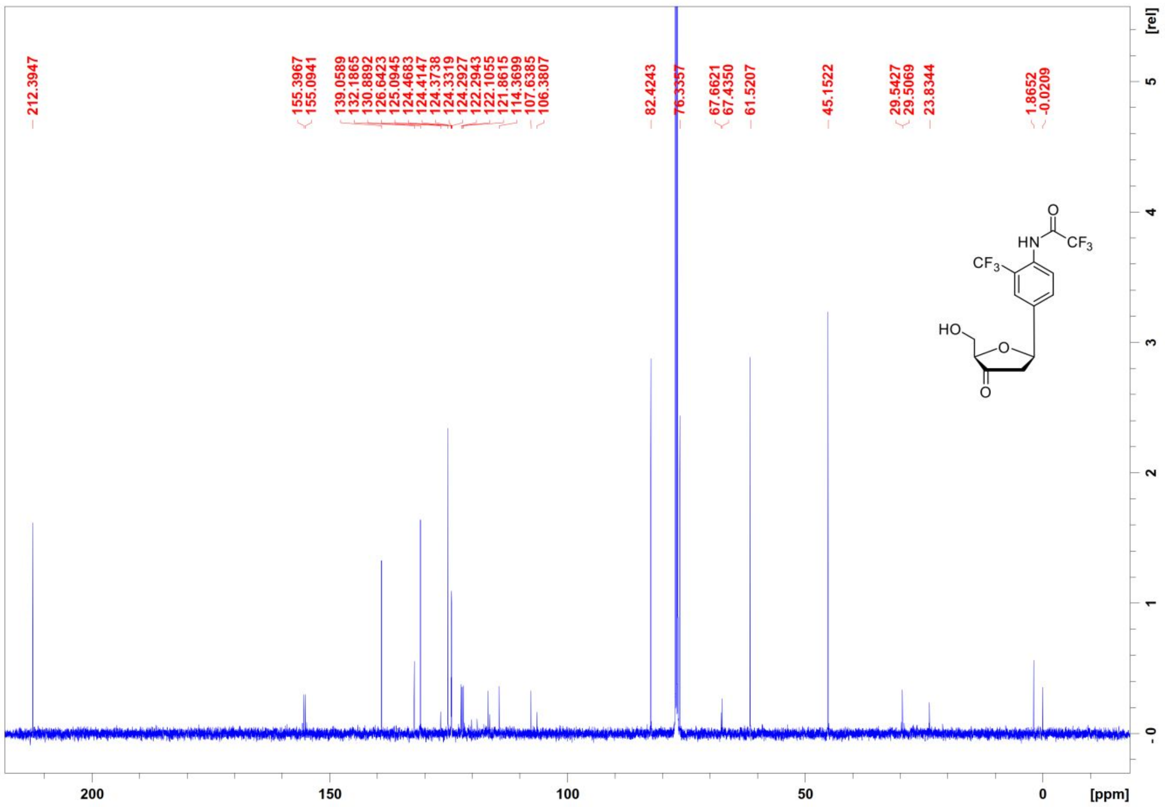

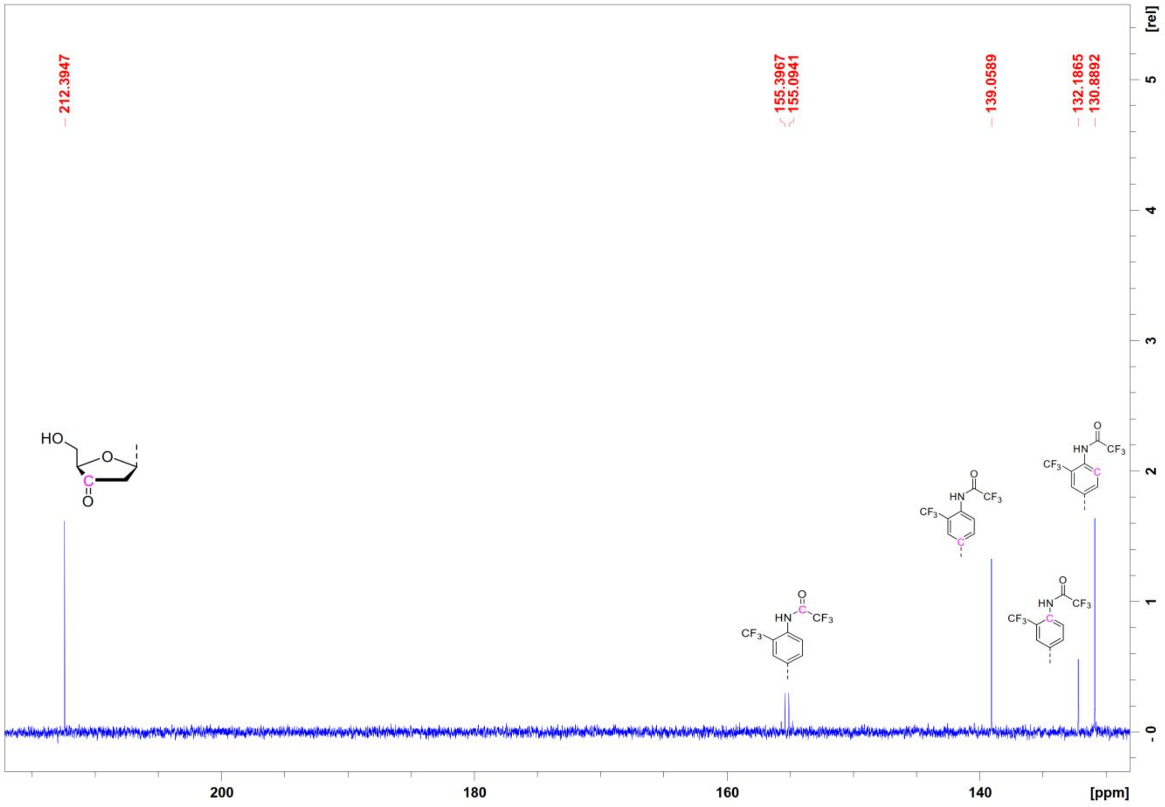

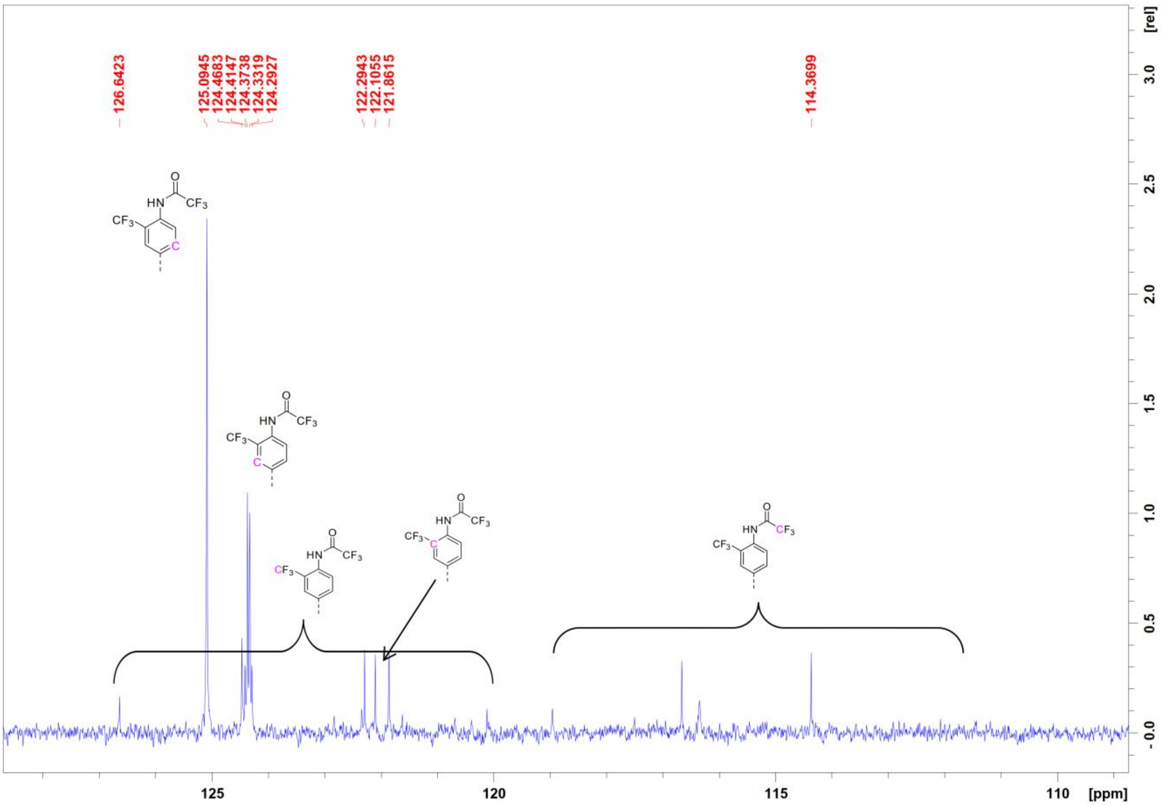
126.6423
125.0945
124.4683
124.4147
124.3738
124.3319
124.2927
122.2943
122.1055
121.8615
114.3699
HN
CF3
CF3
O
C
125
120
115
110
[ppm]
[rel]
3.0
2.5
2.0
1.5
1.0
0.5
0.0

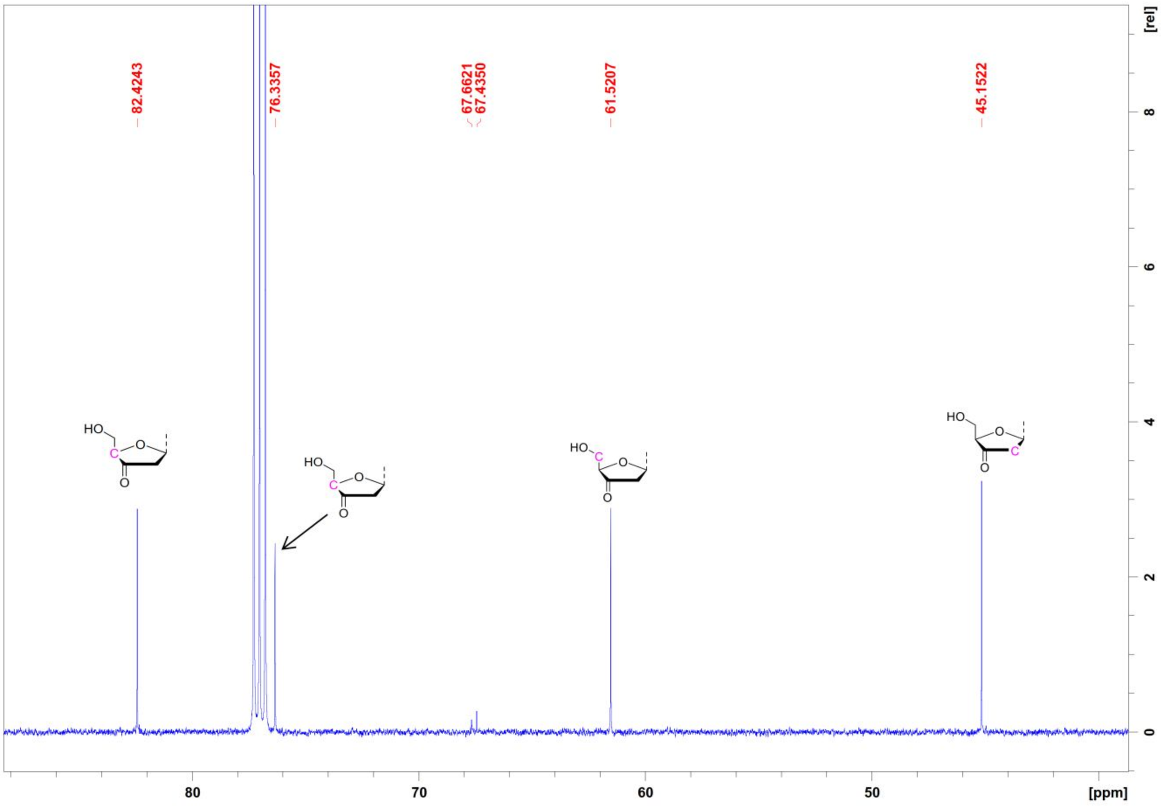
82.4243
76.3357
67.6621
67.4350
61.5207
45.1522
HO
O
C
80
70
60
50
[ppm]
[rel]
8
6
4
2
0

^{1}H NMR spectrum of compound 8 (500 MHz, CD_3CN)

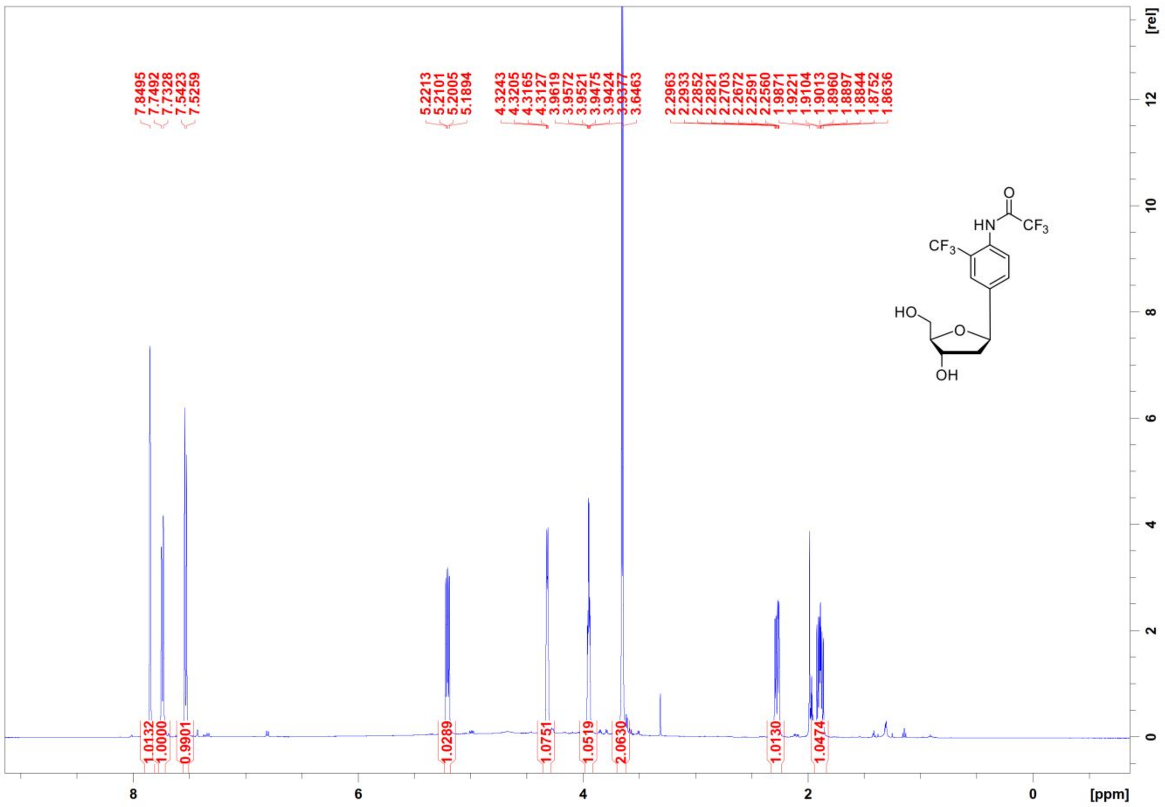

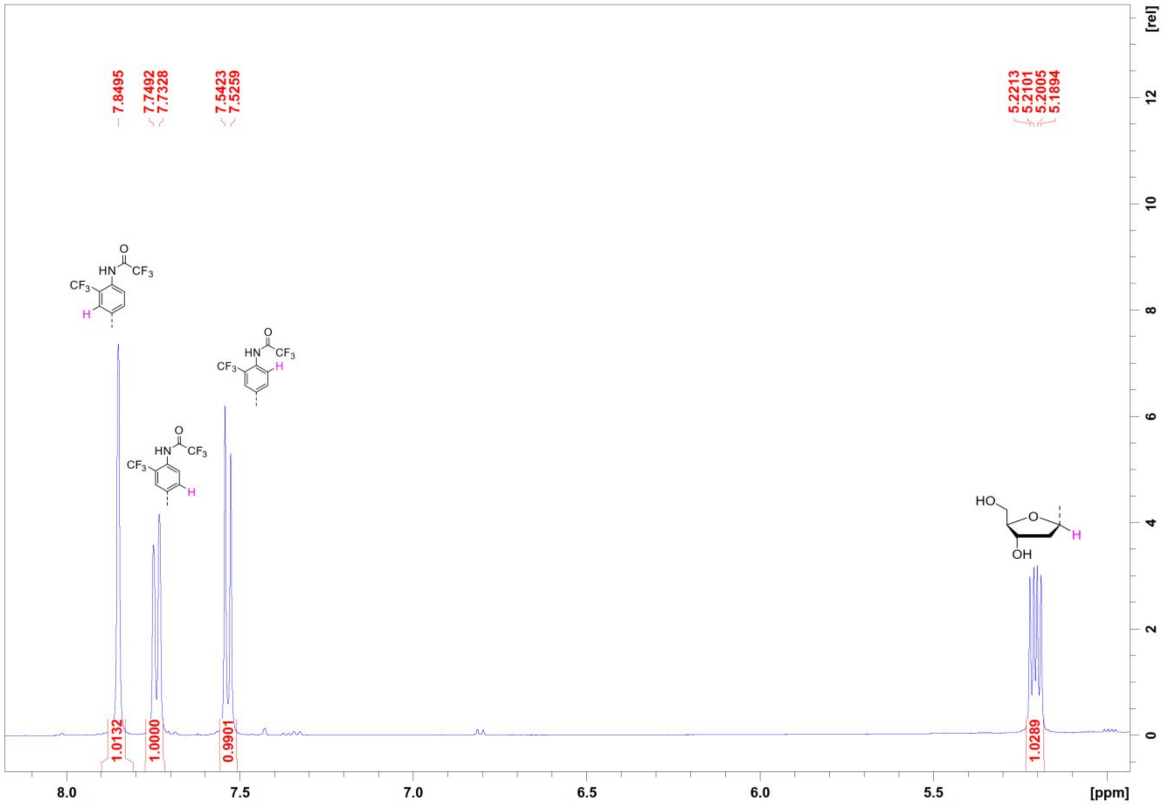

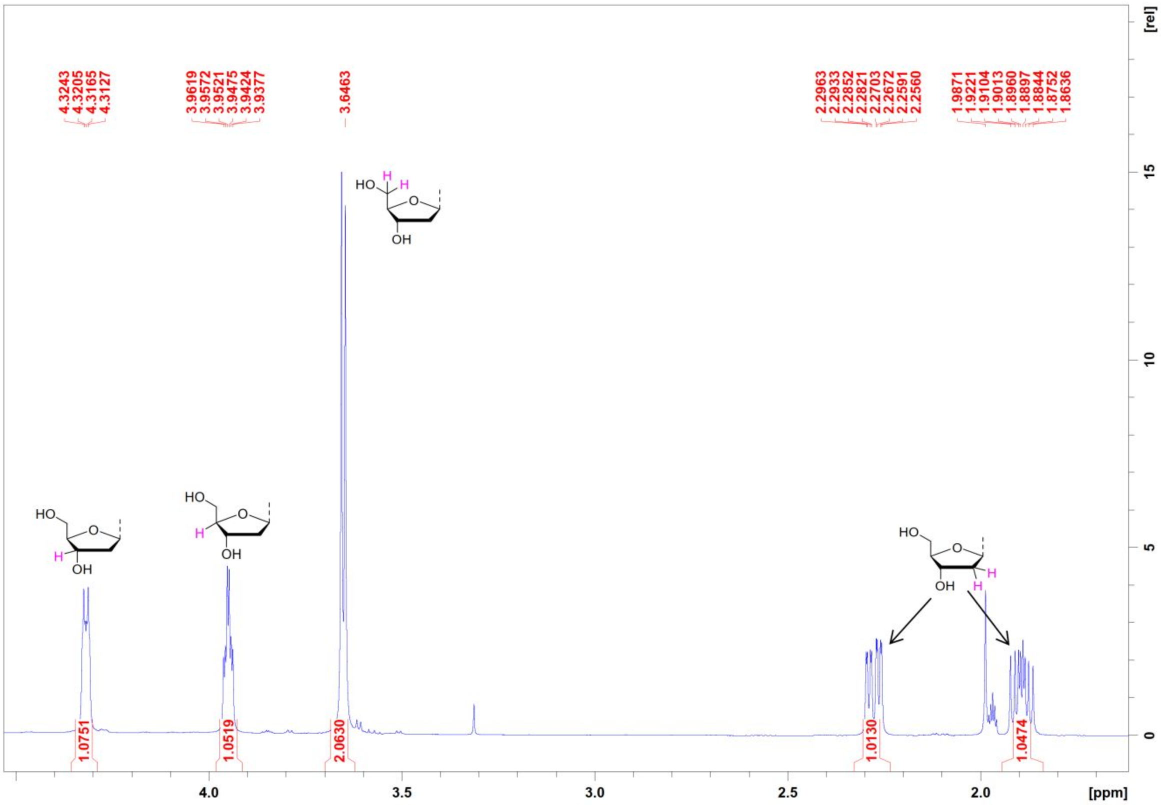
4.3243
4.3205
4.3165
4.3127
3.9619
3.9572
3.9521
3.9475
3.9424
3.9377
3.6463
2.2963
2.2933
2.2852
2.2821
2.2703
2.2672
2.2591
2.2560
1.9871
1.9221
1.9104
1.9013
1.8960
1.8897
1.8844
1.8752
1.8636
HO
O
OH
H
1.0751
1.0519
2.0630
1.0130
1.0474
[rel]
15
10
5
0
4.0
3.5
3.0
2.5
2.0
[ppm]

$^{13}C\{^{1}H\}$ NMR spectrum of compound 8 (125 MHz, CD_3CN)

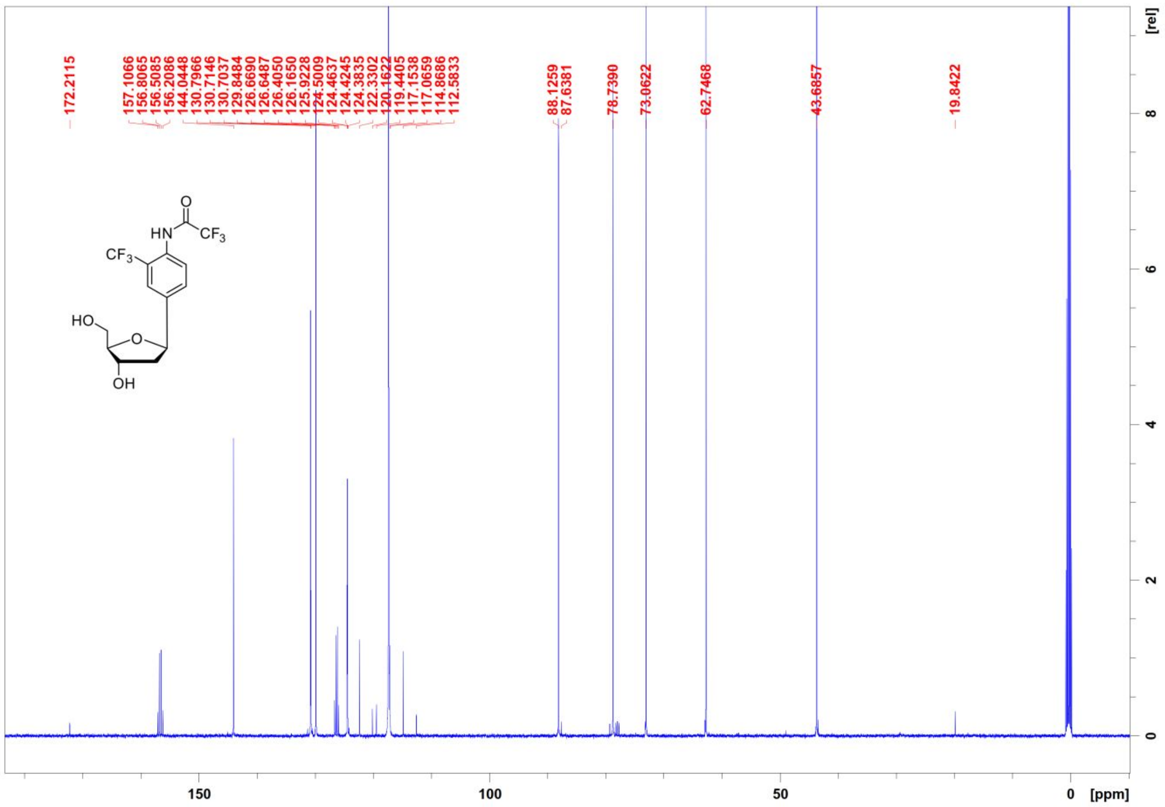

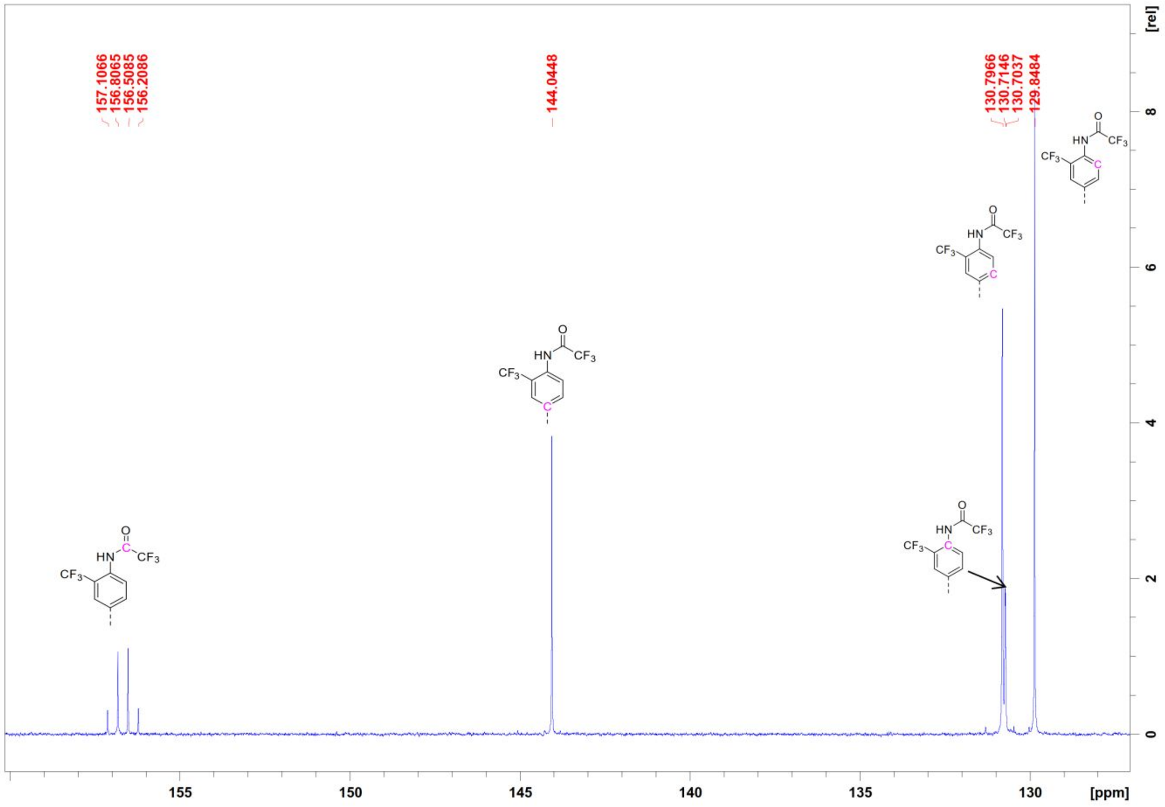

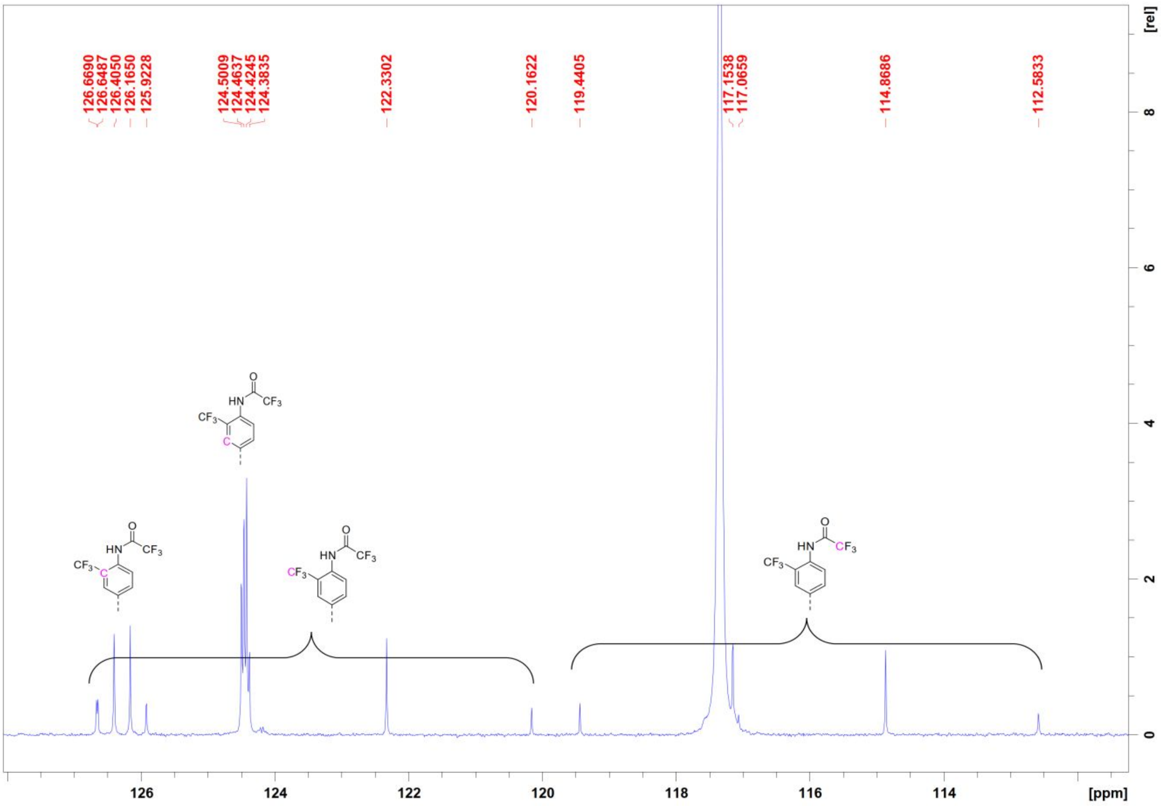
126.6690
126.6487
126.4050
126.1650
125.9228
124.5009
124.4637
124.4245
124.3835
122.3302
120.1622
119.4405
117.1538
117.0659
114.8686
112.5833
[rel]
[ppm]

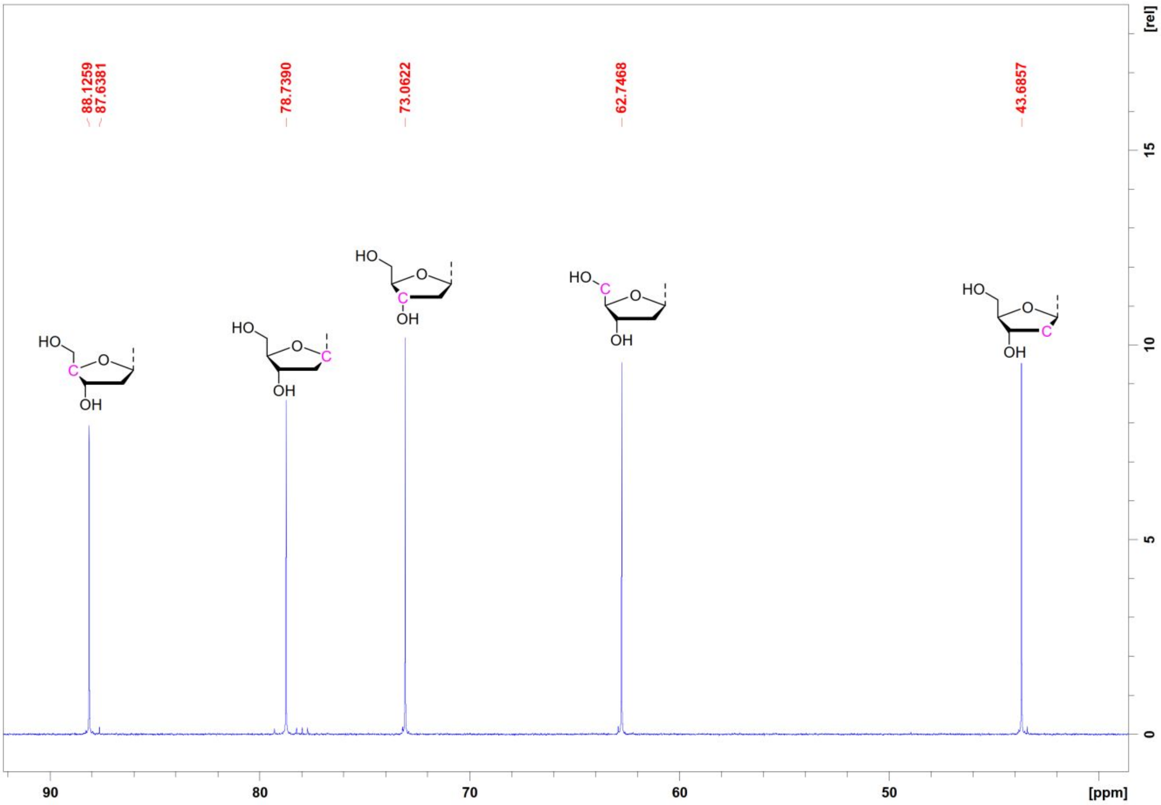
88.1259
87.6381
78.7390
73.0622
62.7468
43.6857
[rel]
[ppm]

^{19}F NMR spectrum of compound 8 (470 MHz, $CDCl_3$)

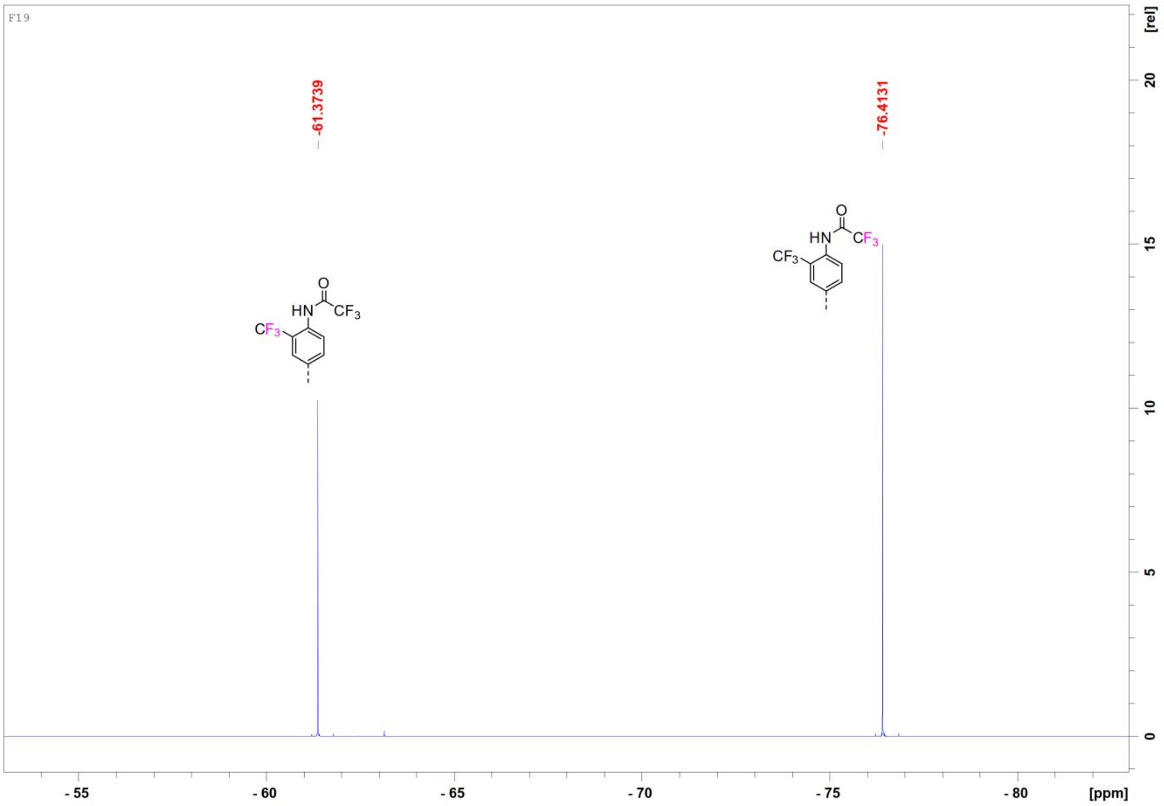

^{1}H NMR spectrum of compound 9 (500 MHz, $CDCl_3$)

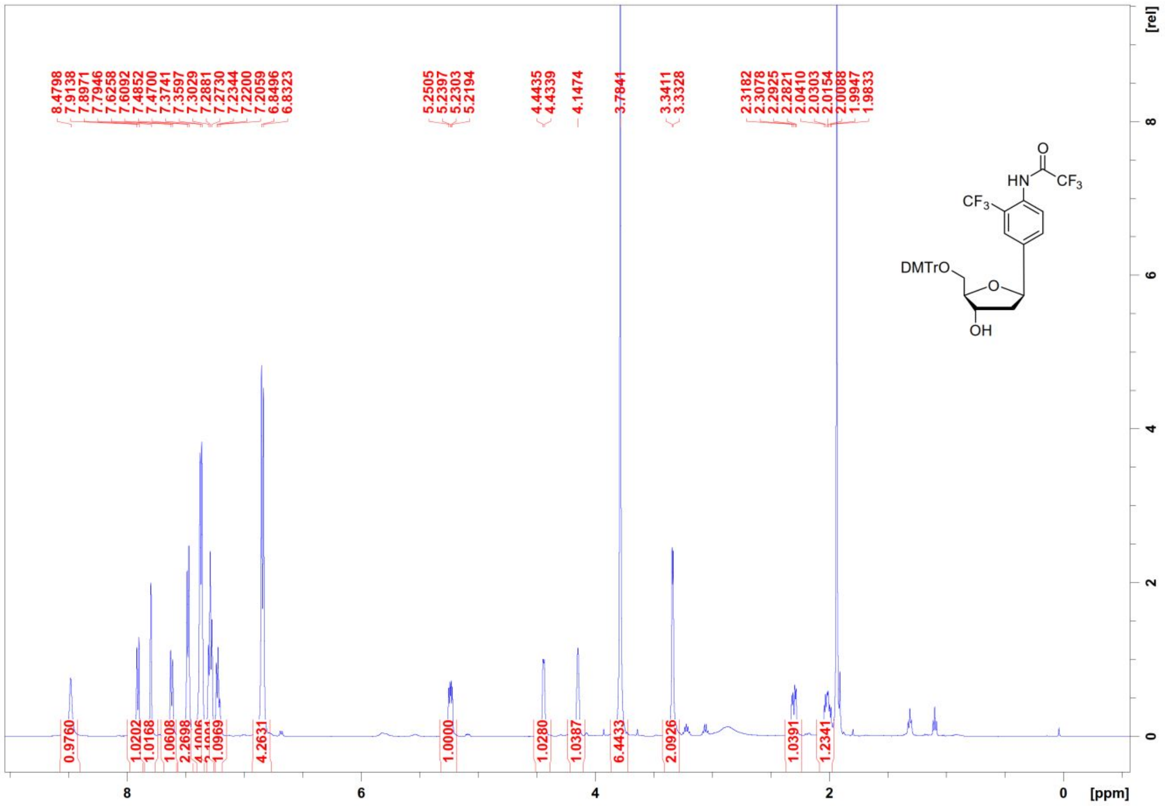

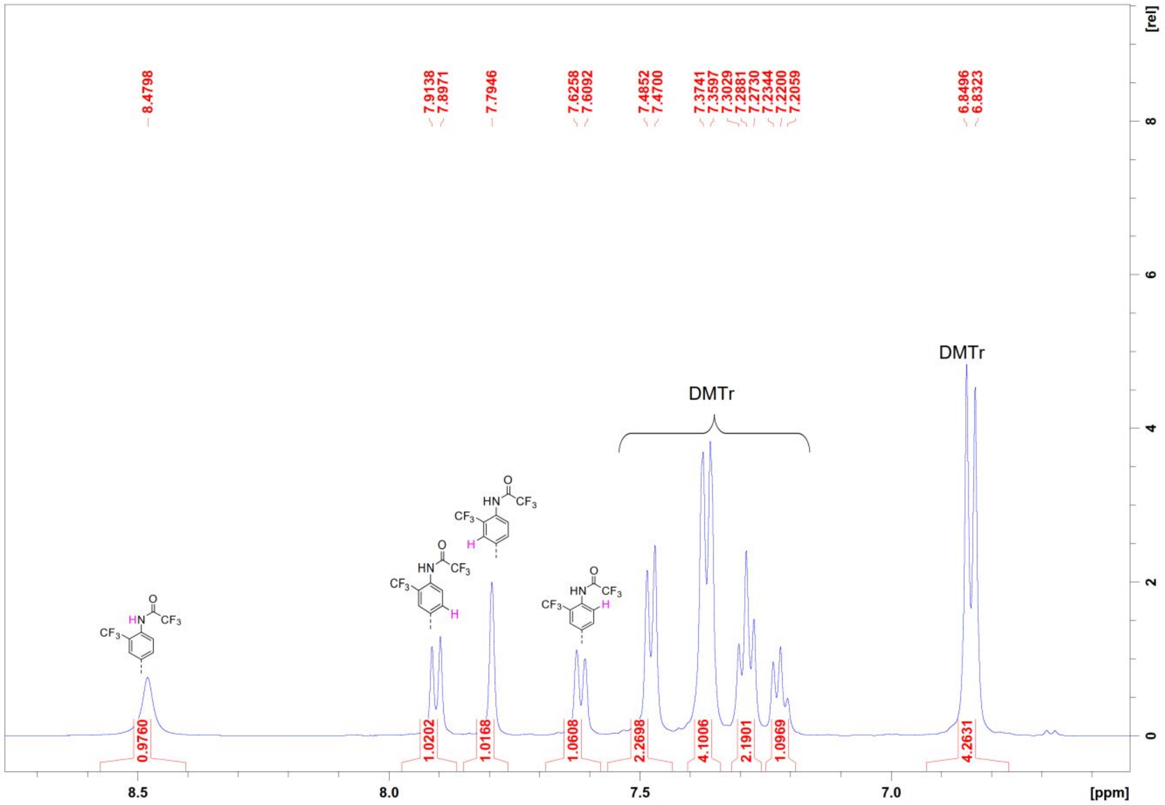

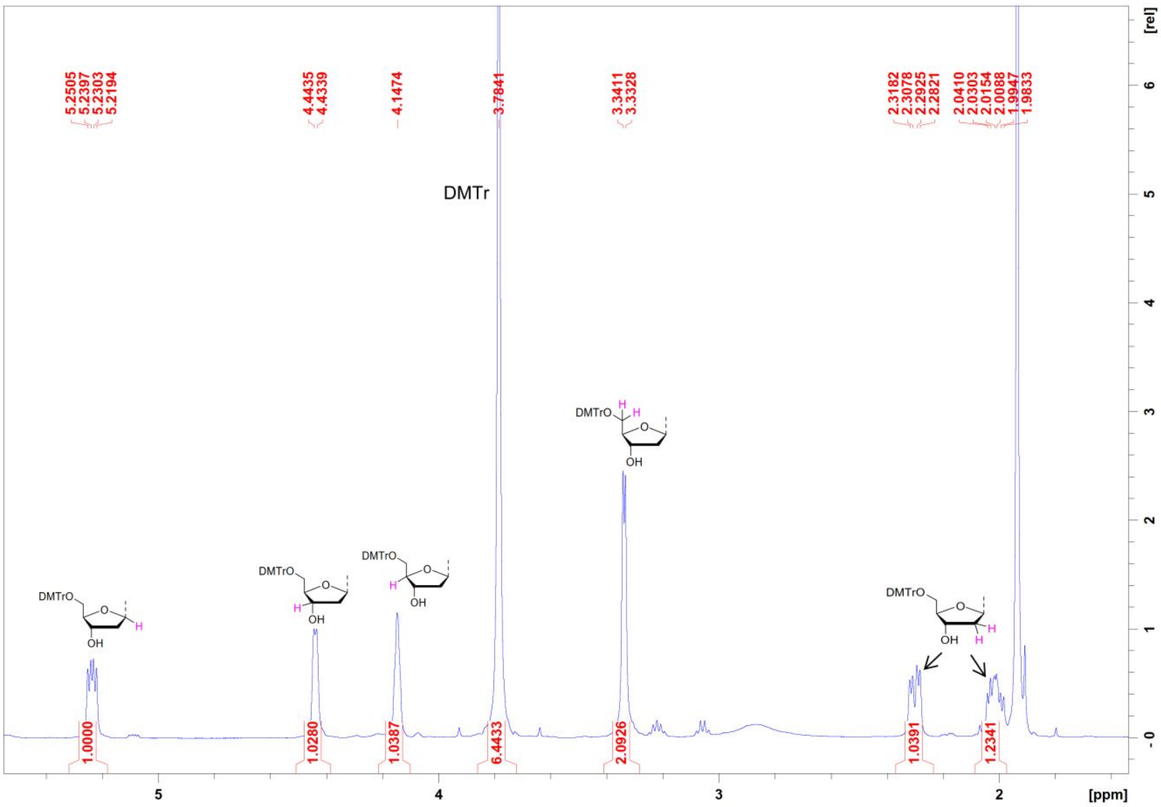
5.2505
5.2397
5.2303
5.2194
4.4435
4.4339
4.1474
3.7841
3.3411
3.3328
2.3182
2.3078
2.2925
2.2821
2.0410
2.0303
2.0154
2.0088
1.9947
1.9833
DMTr
DMTrO
OH
H
O
1.0000
1.0280
1.0387
6.4433
2.0926
1.0391
1.2341
[rel]
[ppm]

$^{13}C\{^1H\}$ NMR spectrum of compound 9 (125 MHz, $CDCl_3$)

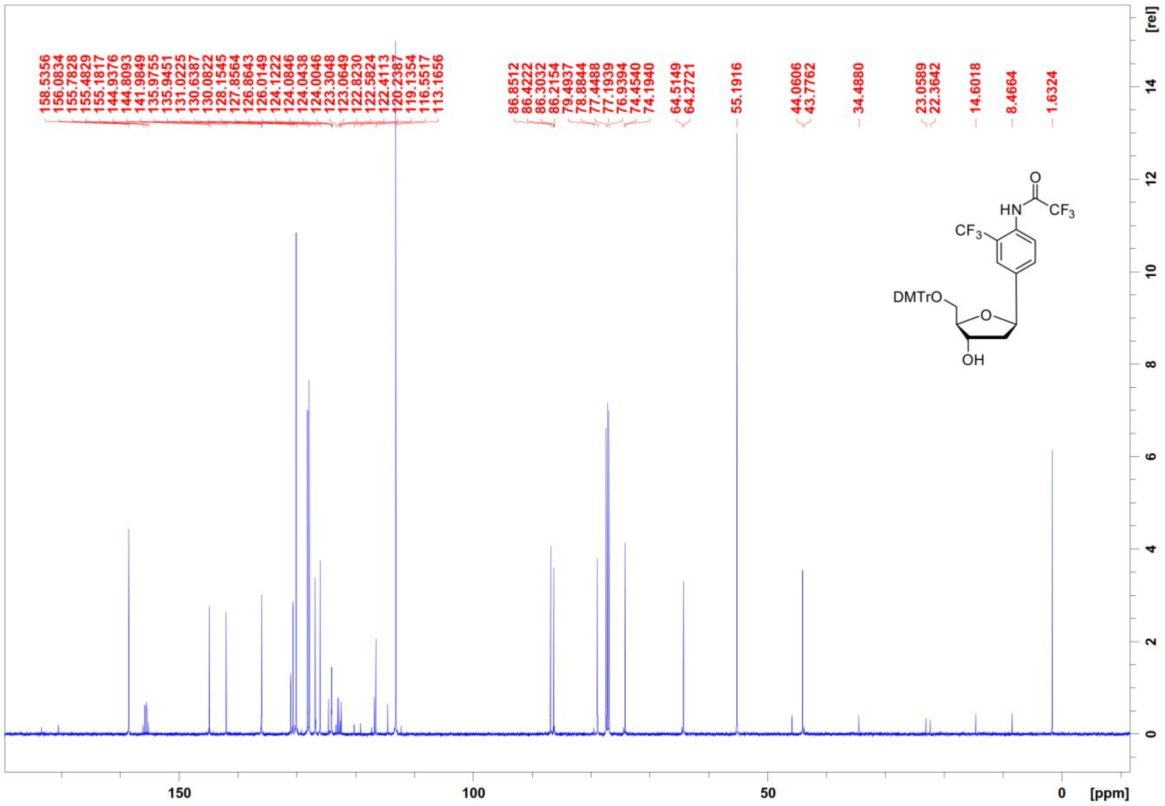

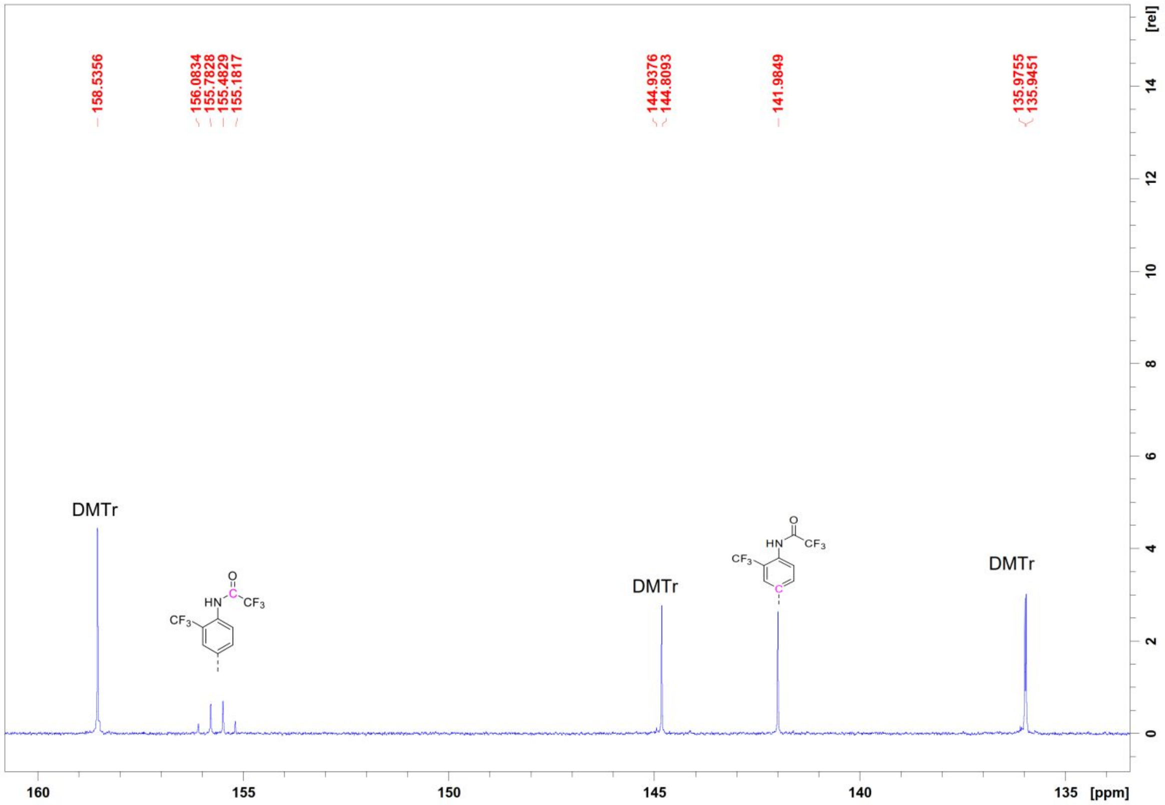

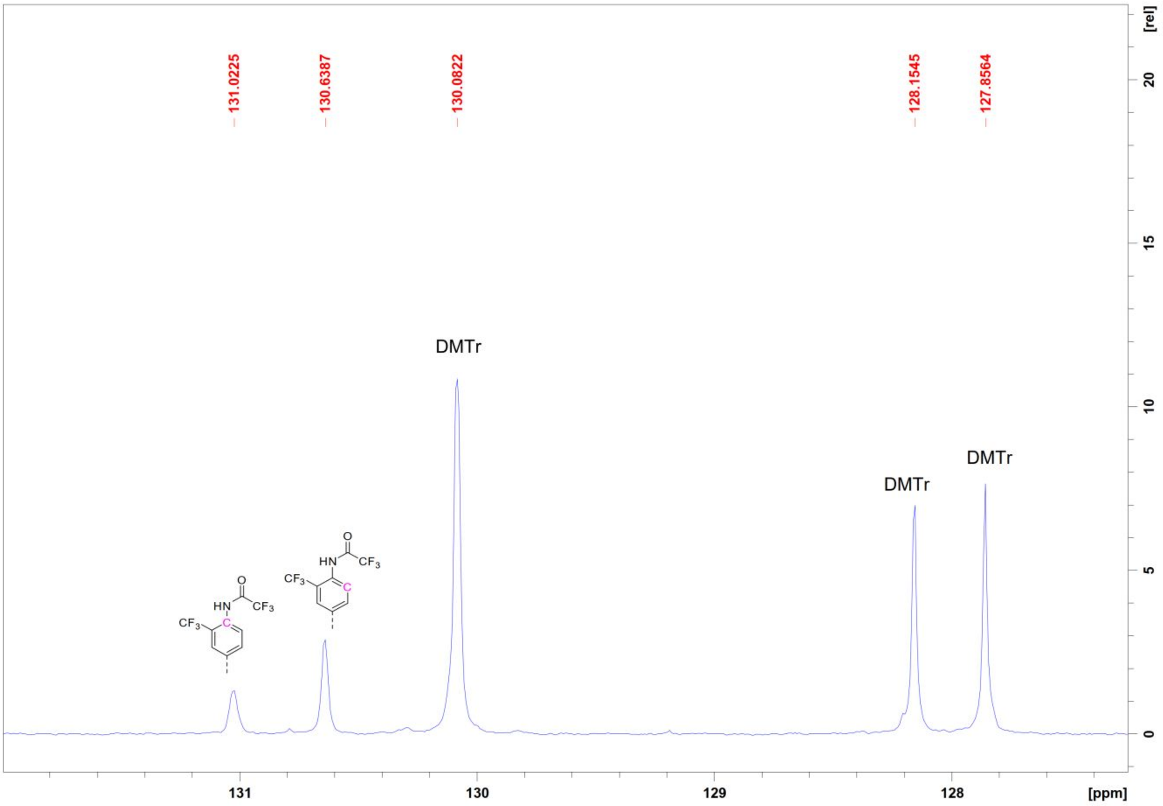

131.0225
130.6387
130.0822
128.1545
127.8564
DMTr
DMTr
DMTr
[rel]
[ppm]

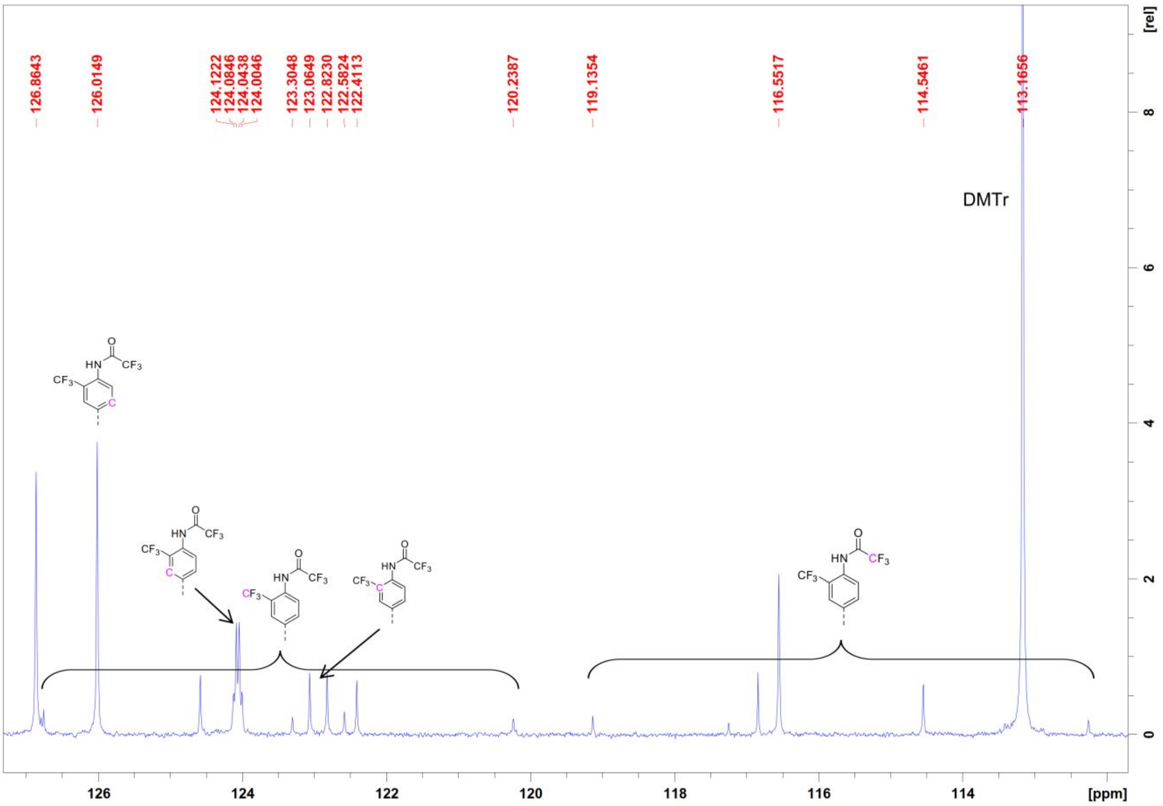

126.8643
126.0149
124.1222
124.0846
124.0438
124.0046
123.3048
123.0649
122.8230
122.5824
122.4113
120.2387
119.1354
116.5517
114.5461
113.1656
DMTr
[rel]
[ppm]

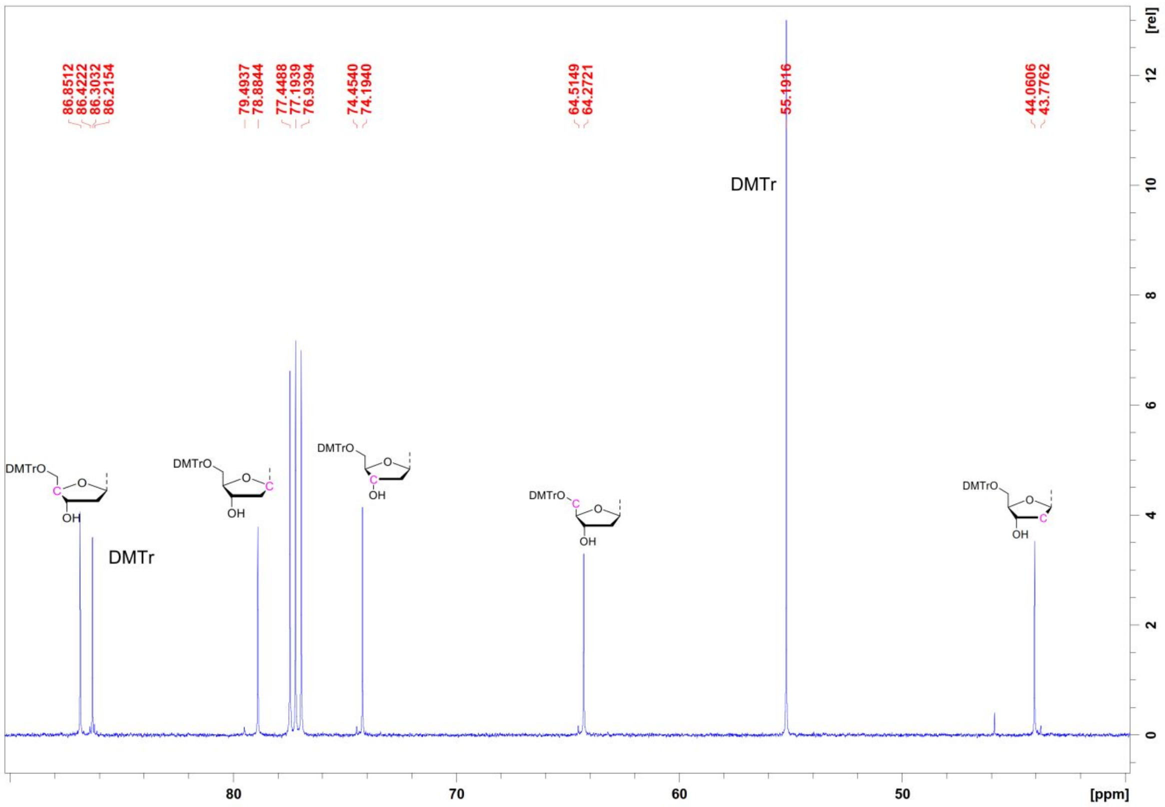

86.8512
86.4222
86.3032
86.2154
79.4937
78.8844
77.4488
77.1939
76.9394
74.4540
74.1940
64.5149
64.2721
55.1916
44.0606
43.7762
DMTr
DMTr
DMTrO
OH
[rel]
[ppm]

^{19}F NMR spectrum of compound 9 (470 MHz, *d_6*-DMSO)

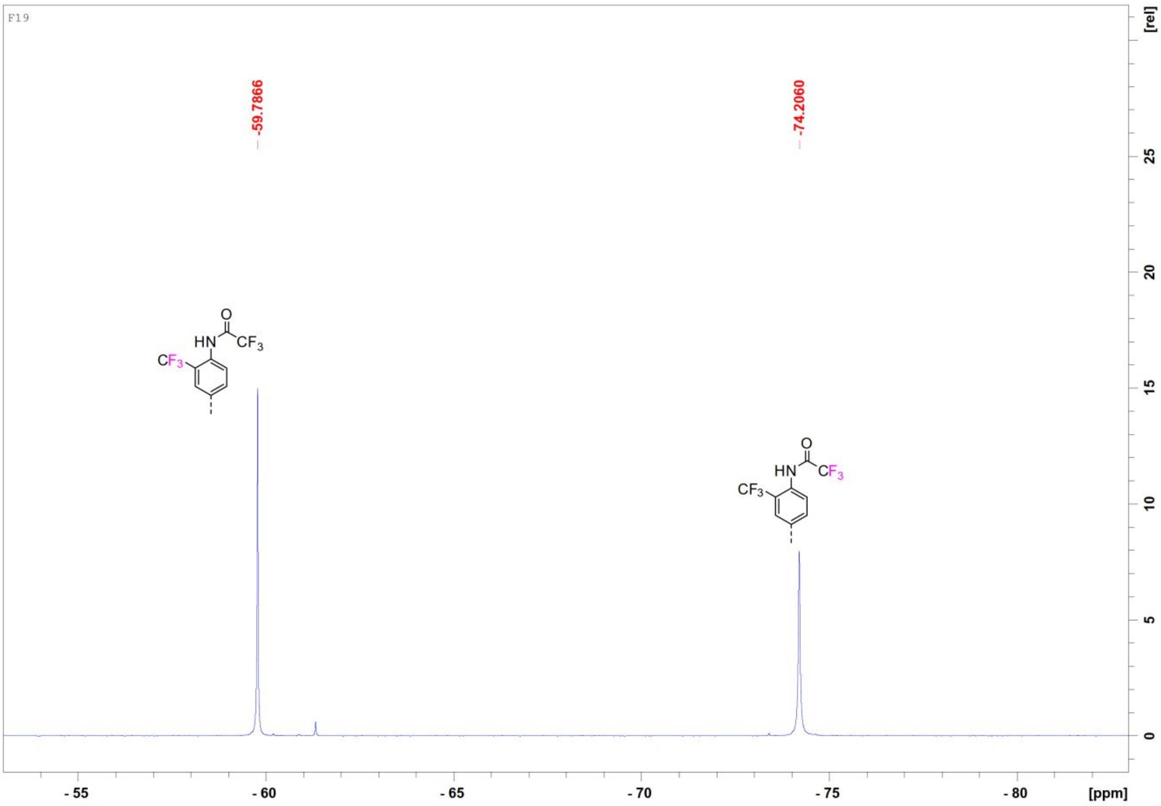

^{1}H NMR spectrum of compound 3 (500 MHz, $CDCl_3$)

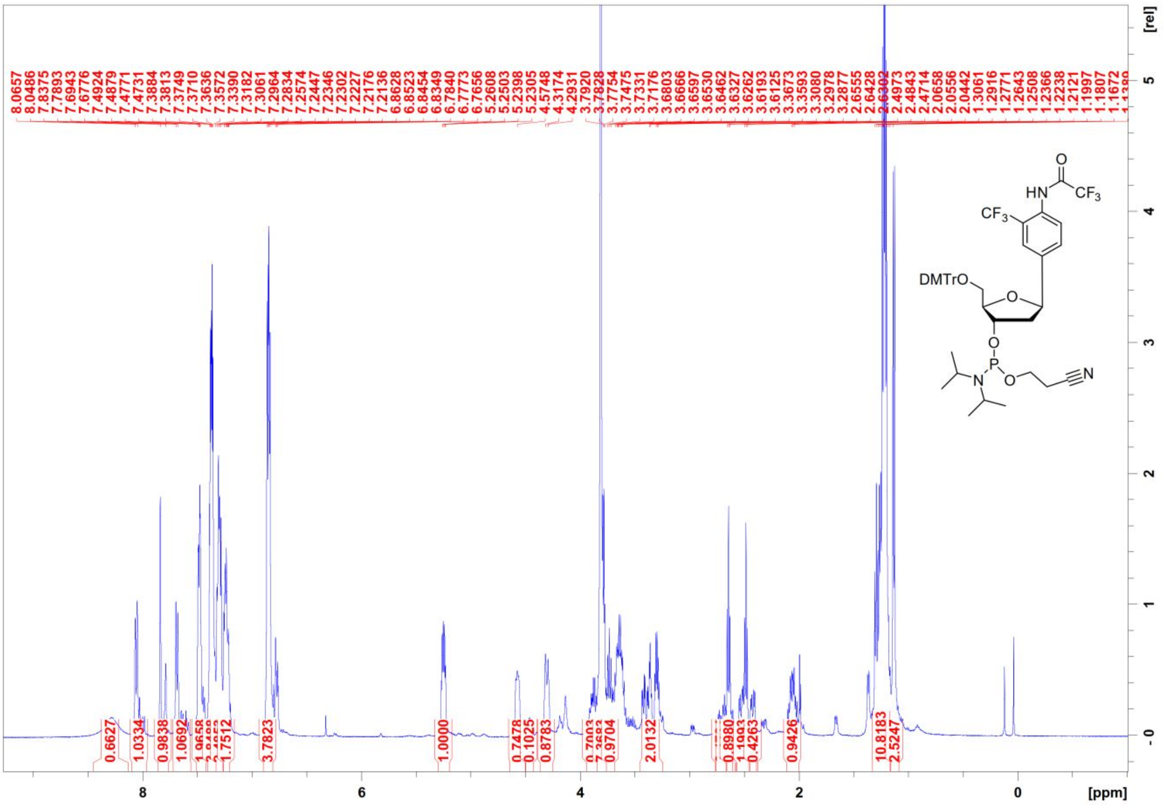

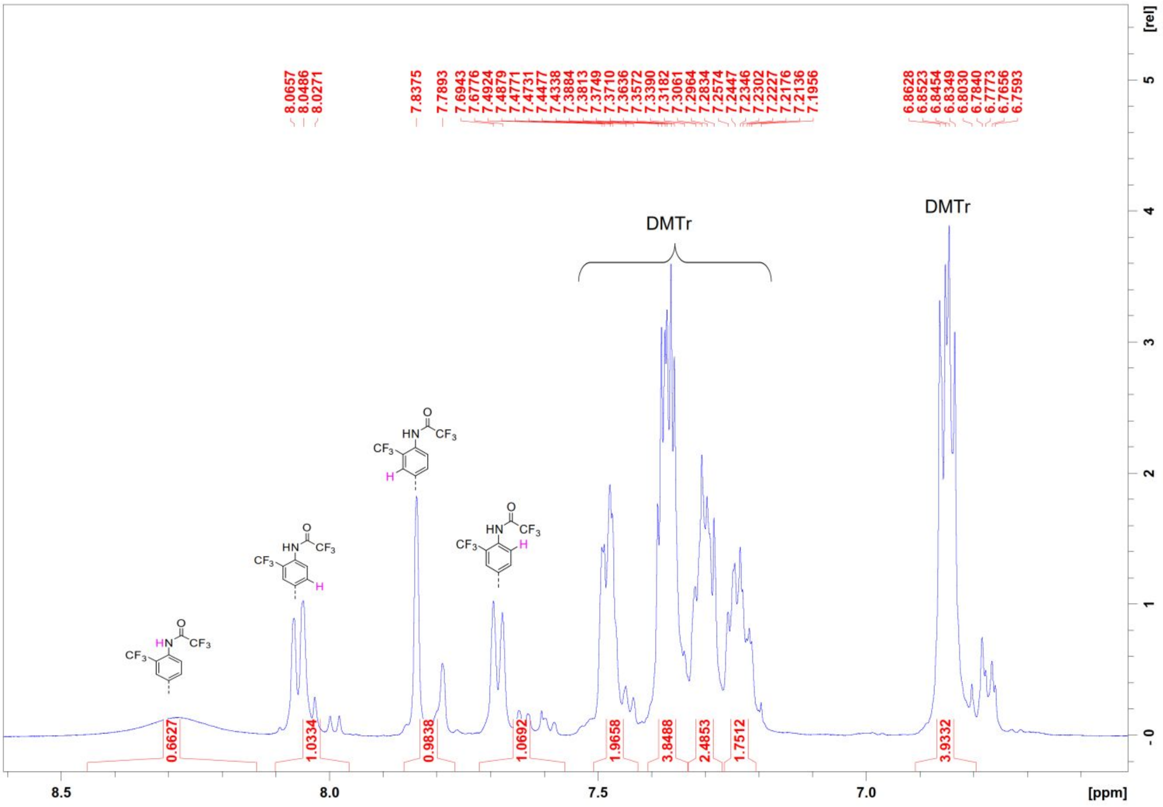

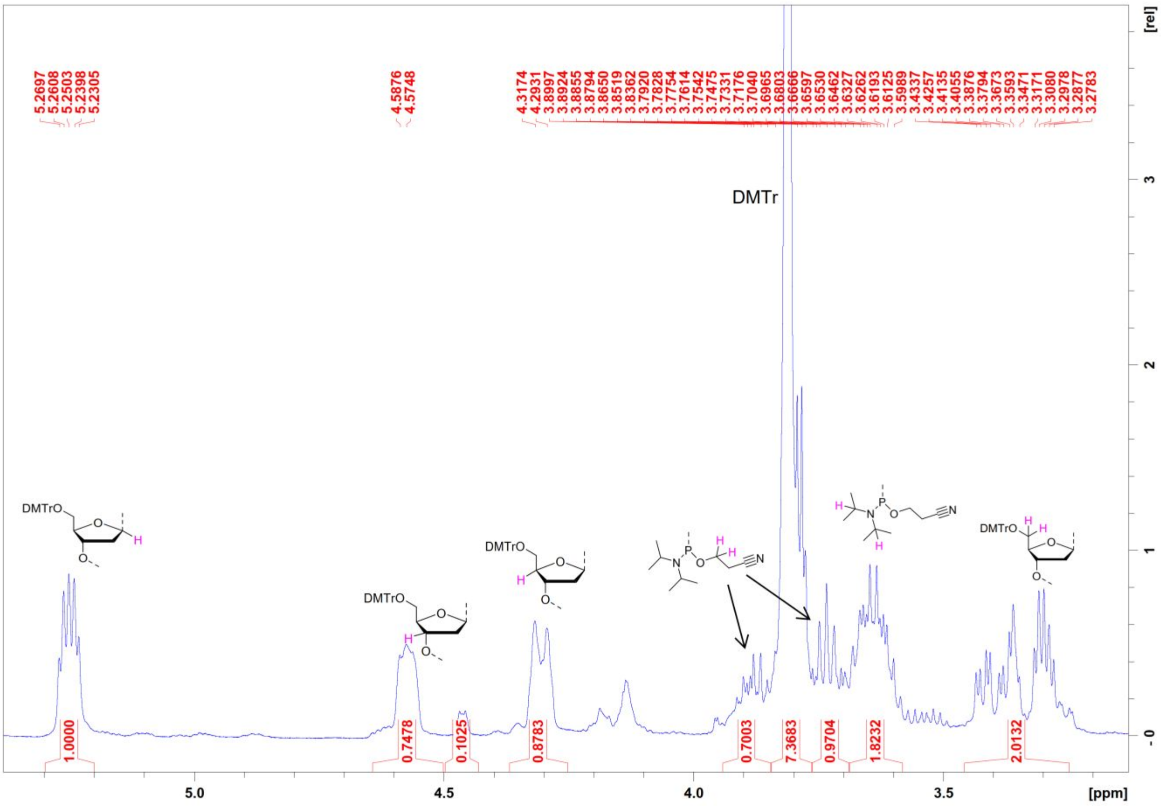
5.2697 5.2608 5.2503 5.2398 5.2305
4.5876 4.5748
4.3174 4.2931 3.8997 3.8924 3.8855 3.8794 3.8650 3.8519 3.8362 3.7920 3.7828 3.7754 3.7614 3.7542 3.7475 3.7331 3.7176 3.7040 3.6965 3.6803 3.6666 3.6597 3.6530 3.6462 3.6327 3.6262 3.6193 3.6125 3.5989 3.4337 3.4257 3.4135 3.4055 3.3876 3.3794 3.3673 3.3593 3.3471 3.3171 3.3080 3.2978 3.2877 3.2783
DMTr
DMTrO
1.0000
0.7478
0.1025
0.8783
0.7003
7.3683
0.9704
1.8232
2.0132
[rel]
[ppm]

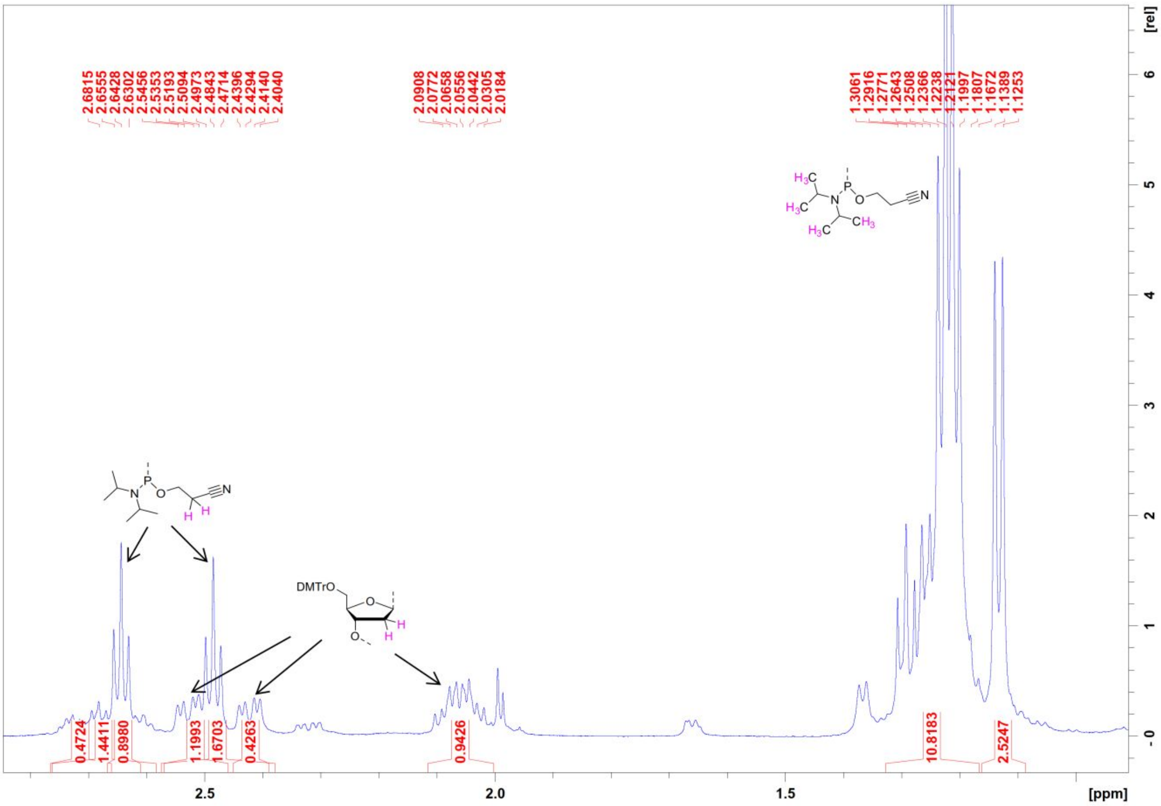
2.6815 2.6555 2.6428 2.6302 2.5456 2.5353 2.5193 2.5094 2.4973 2.4843 2.4714 2.4396 2.4294 2.4140 2.4040
2.0908 2.0772 2.0658 2.0556 2.0442 2.0305 2.0184
1.3061 1.2916 1.2771 1.2643 1.2508 1.2366 1.2238 1.2121 1.1997 1.1807 1.1672 1.1389 1.1253
DMTrO
0.4724
1.4411
0.8980
1.1993
1.6703
0.4263
0.9426
10.8183
2.5247
[rel]
[ppm]

$^{13}C\{^{1}H\}$ NMR spectrum of compound 3 (125 MHz, $CDCl_3$)

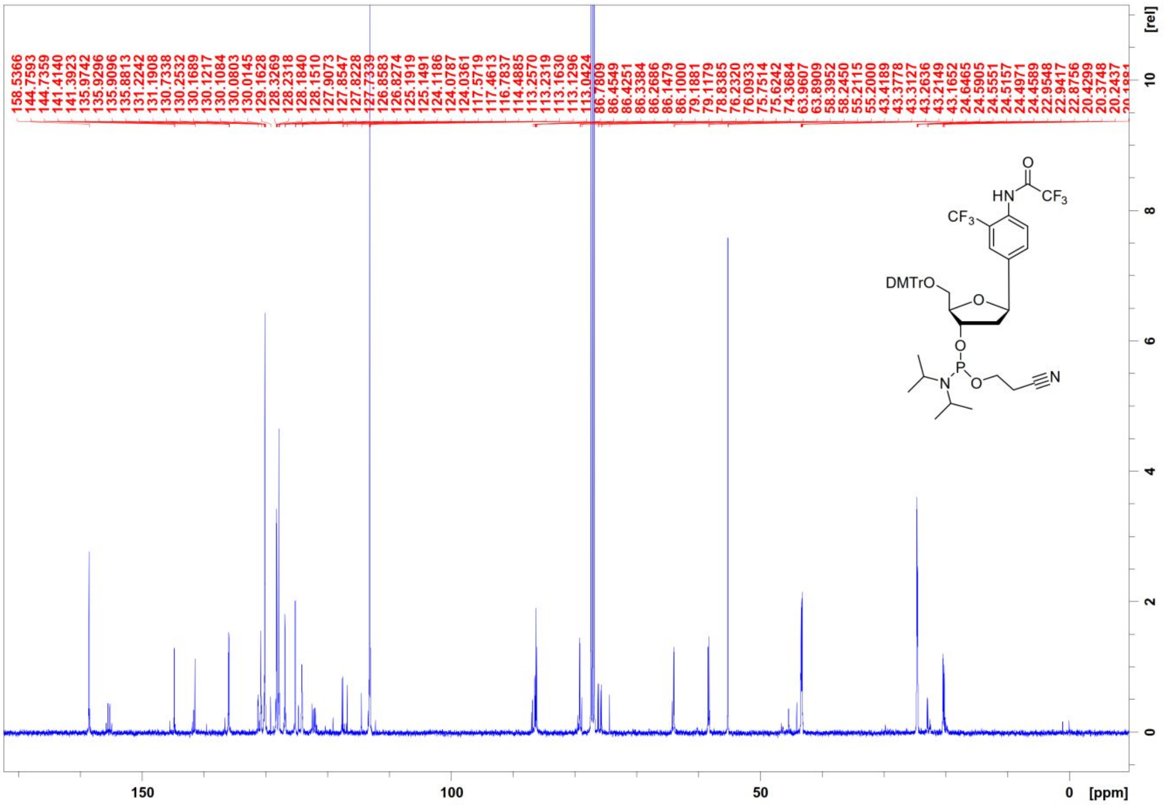

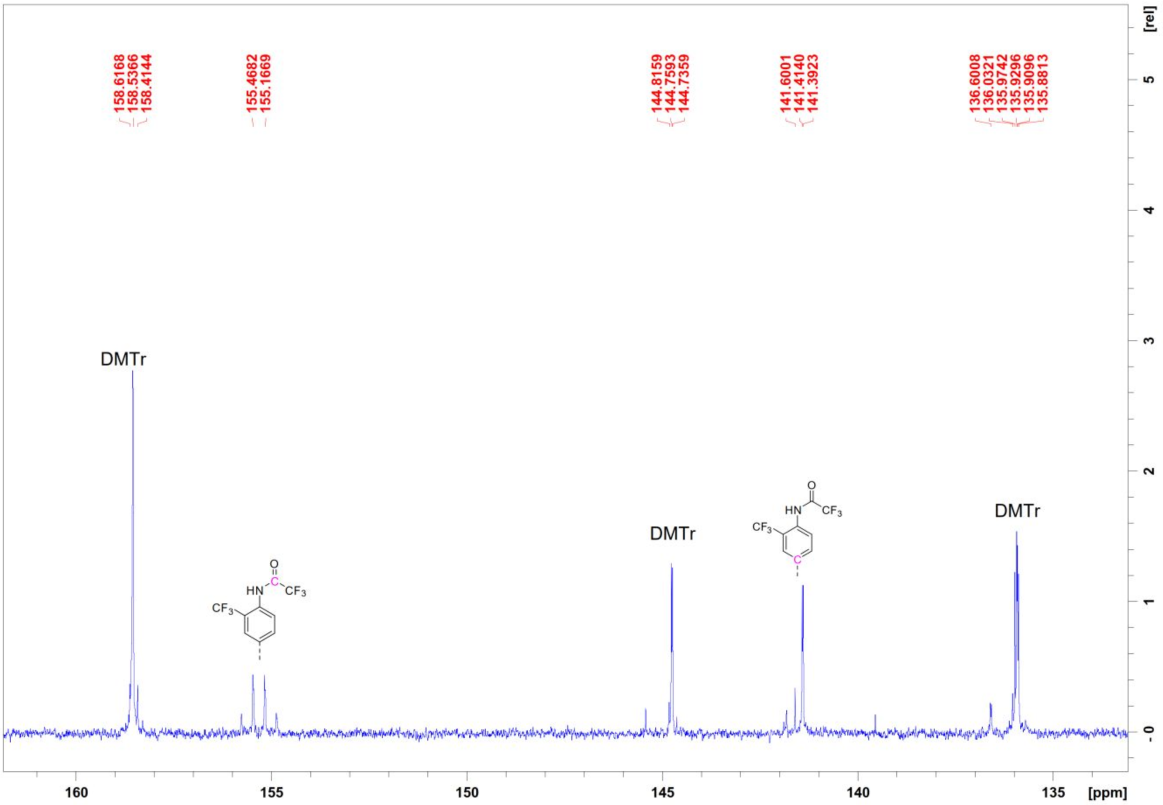

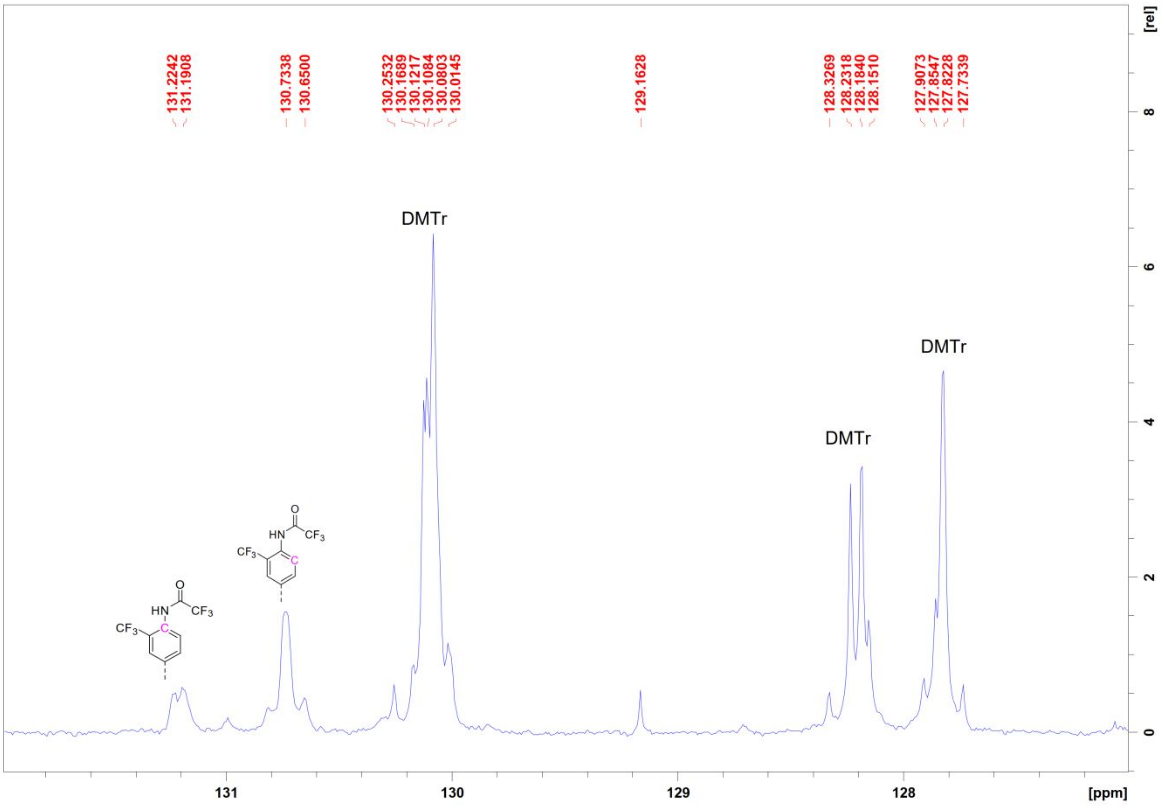
131.2242
131.1908
130.7338
130.6500
130.2532
130.1689
130.1217
130.1084
130.0803
130.0145
129.1628
128.3269
128.2318
128.1840
128.1510
127.9073
127.8547
127.8228
127.7339
DMTr
DMTr
DMTr
[rel]
[ppm]

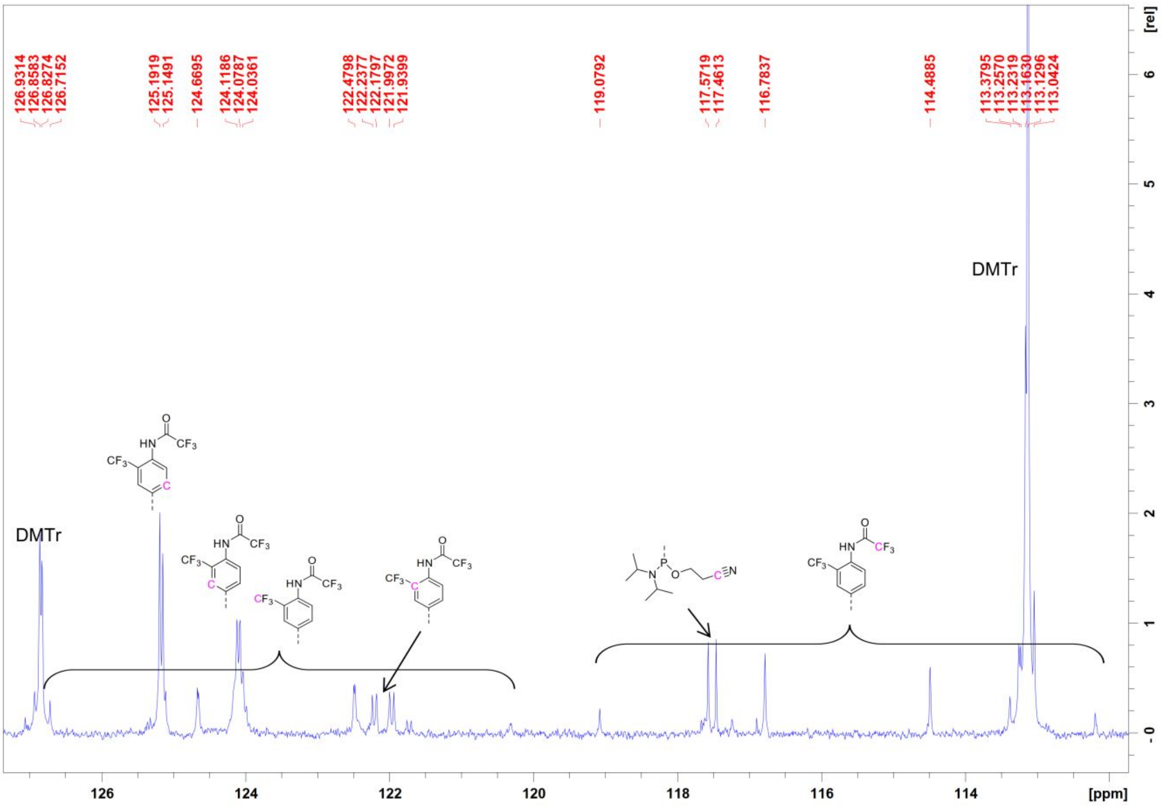
126.9314
126.8583
126.8274
126.7152
125.1919
125.1491
124.6695
124.1186
124.0787
124.0361
122.4798
122.2377
122.1797
121.9972
121.9399
119.0792
117.5719
117.4613
116.7837
114.4885
113.3795
113.2570
113.2319
113.1630
113.1296
113.0424
DMTr
DMTr
[rel]
[ppm]

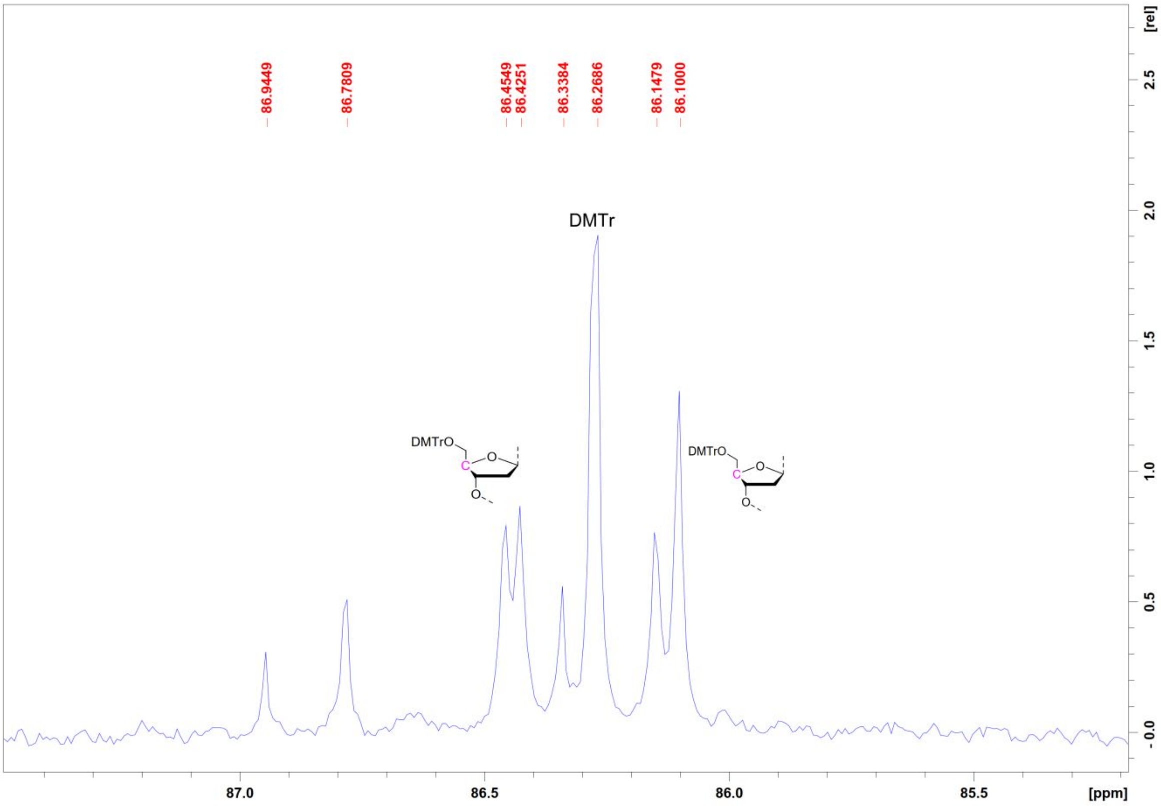
86.9449
86.7809
86.4549
86.4251
86.3384
86.2686
86.1479
86.1000
DMTr
DMTrO
DMTrO
[rel]
[ppm]

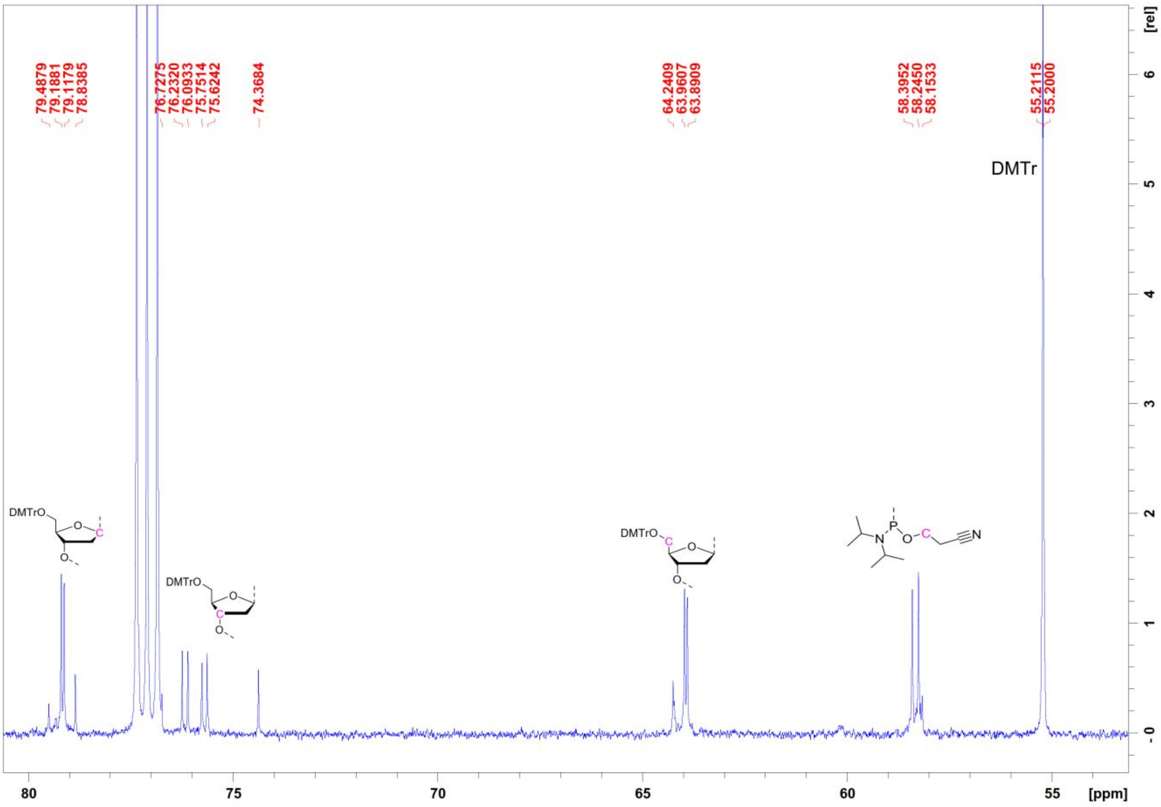
79.4879
79.1881
79.1179
78.8385
76.7275
76.2320
76.0933
75.7514
75.6242
74.3684
64.2409
63.9607
63.8909
58.3952
58.2450
58.1533
55.2115
55.2000
DMTr
DMTrO
DMTrO
DMTrO
N
P
N
[rel]
[ppm]

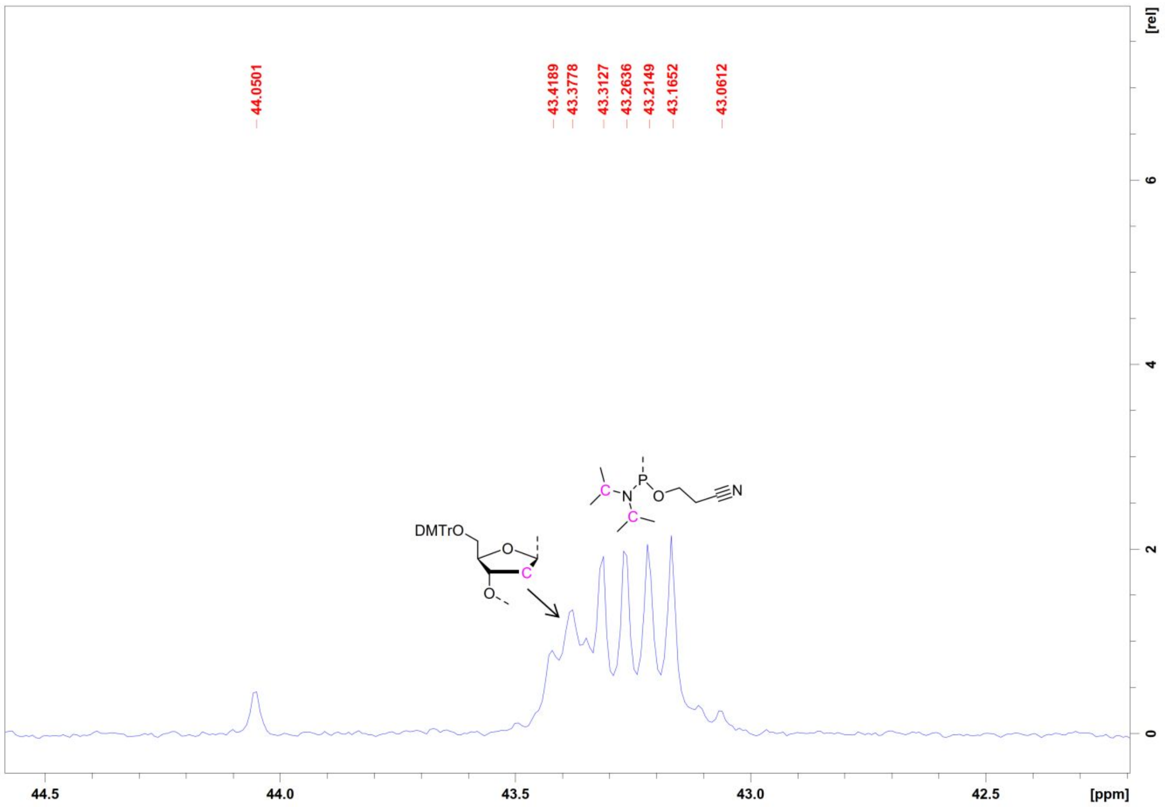
44.0501
43.4189
43.3778
43.3127
43.2636
43.2149
43.1652
43.0612
DMTrO
O
C
[rel]
[ppm]

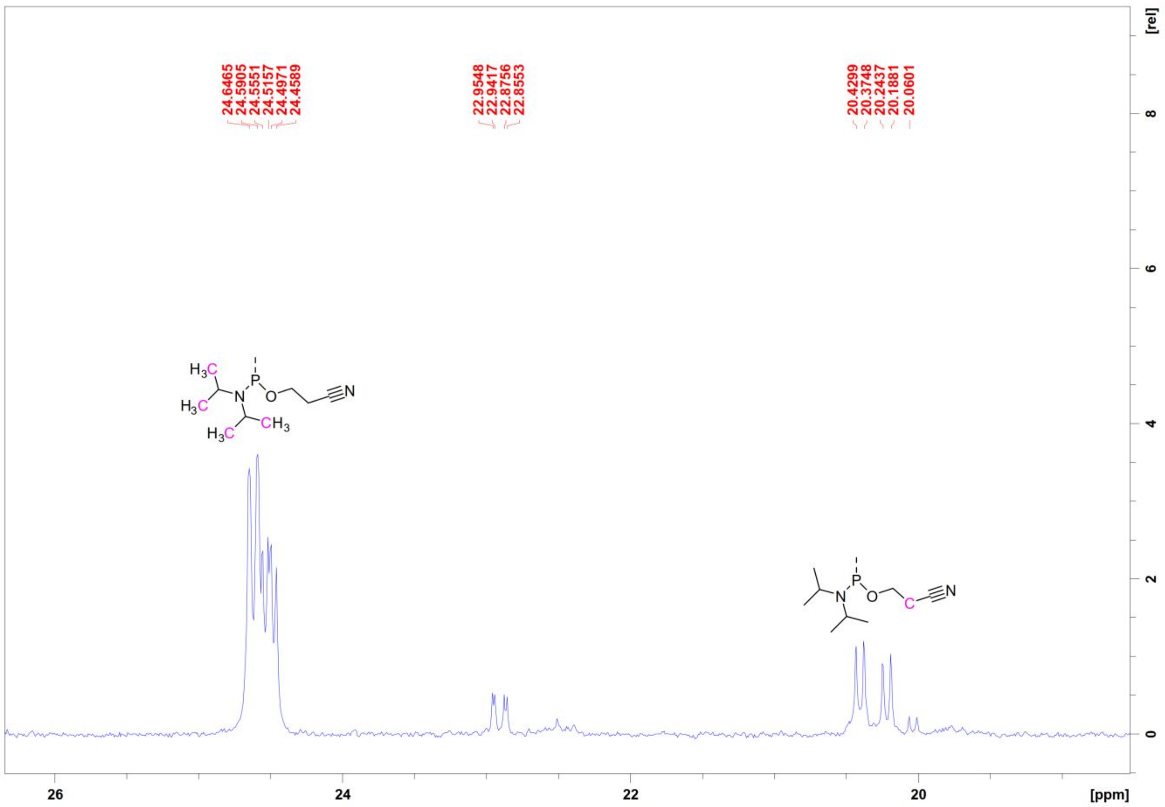
24.6465
24.5905
24.5551
24.5157
24.4971
24.4589
22.9548
22.9417
22.8756
22.8553
20.4299
20.3748
20.2437
20.1881
20.0601
[rel]
[ppm]

^{19}F NMR spectrum of compound 3 (470 MHz, $CDCl_3$)

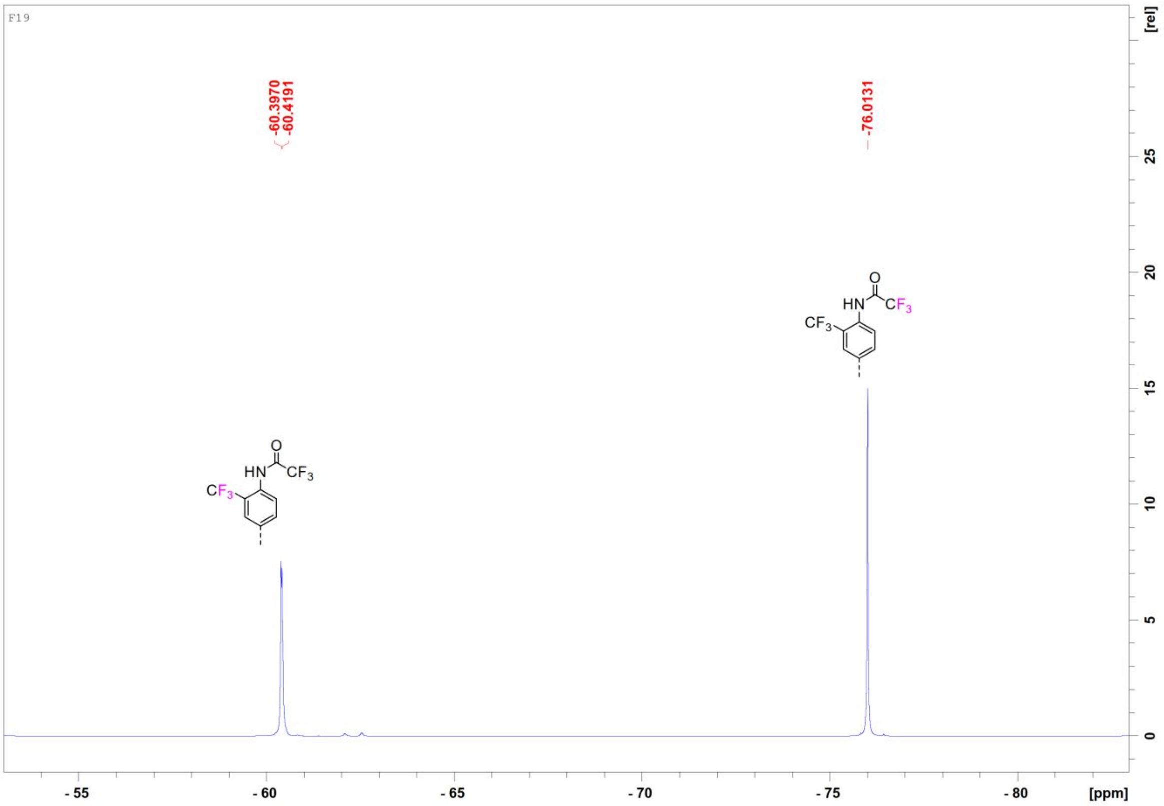

^{31}P NMR spectrum of compound 3 (202 MHz, $CDCl_3$)

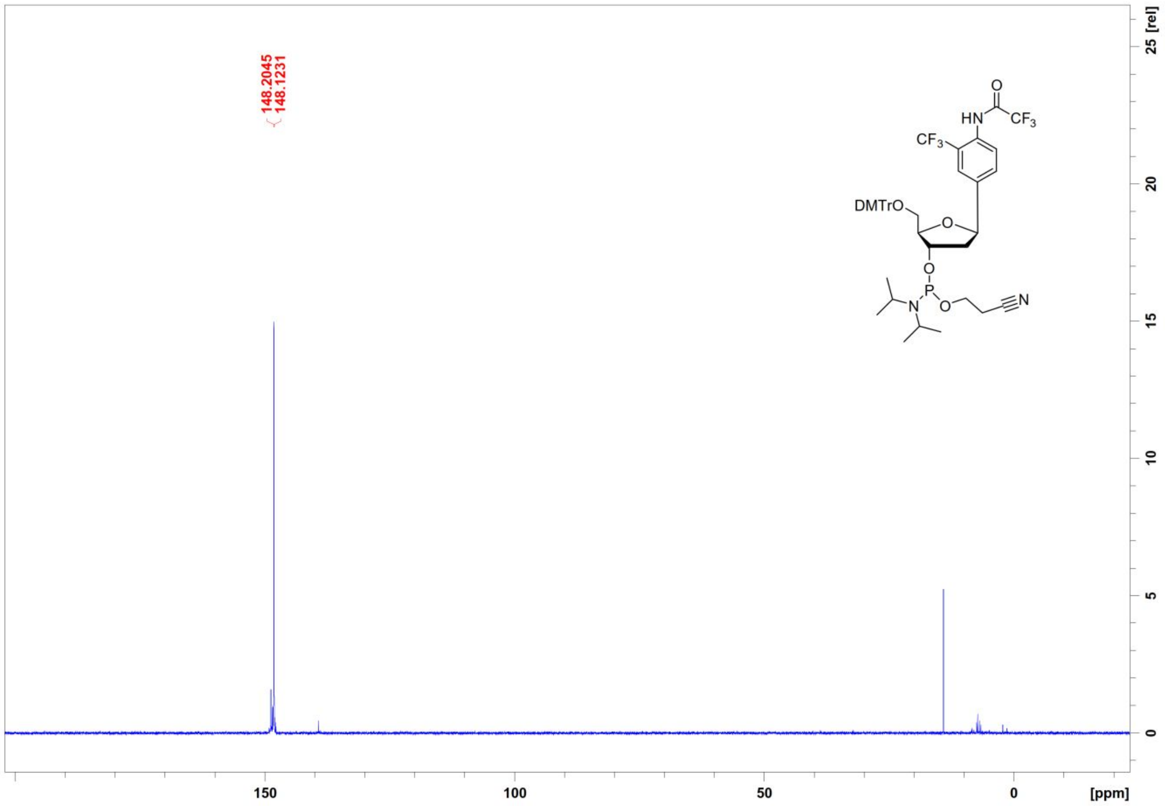

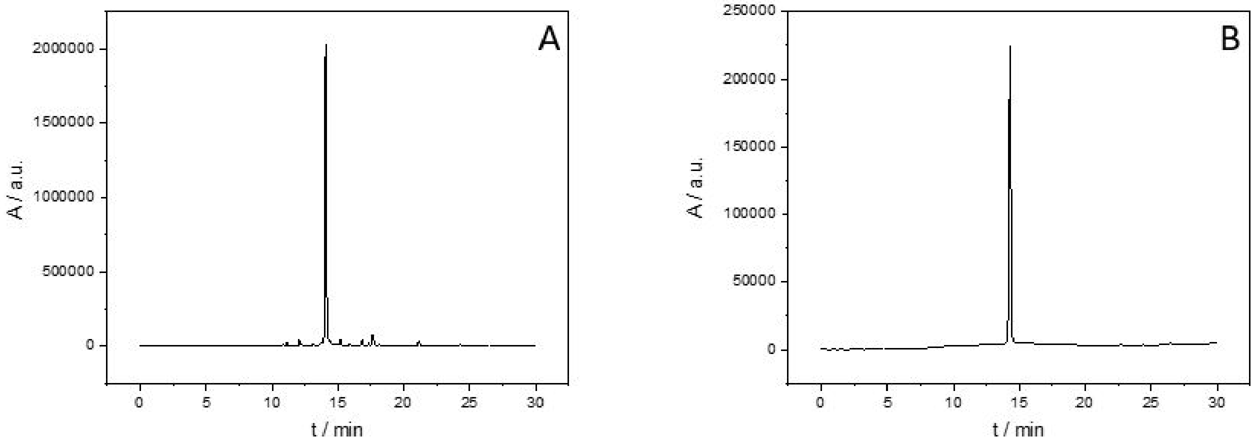

Figure S1. RP-HPLC chromatograms of A) ON(1) crude product and B) purified ON(1).
HPLC conditions: Clarity Oligo-RP C18 column (250 x 10 mm, 10 µm), buffer A: 0.1 M triethylammonium acetate in water, buffer B: 0.1 M triethylammonium acetate in acetonitrile, A gradient elution A/B from 95/5 to 65/35, then from 65/35 (0 – 25 min) to 0/100 (25 – 30 min), flow rate 3 mL/min, l = 260 nm tR = 14.2 min.

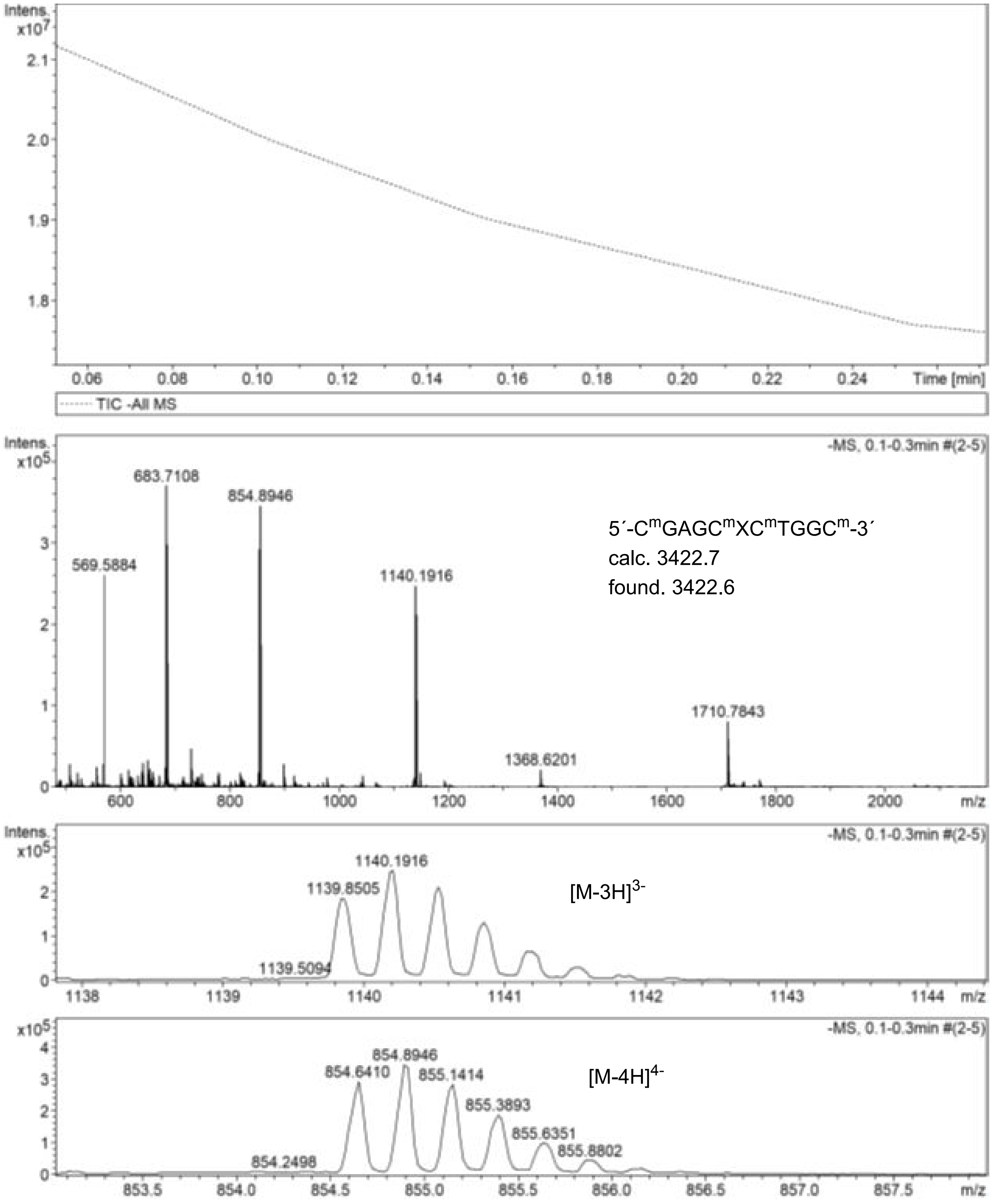

Figure S2. MS spectrum of ON(1)

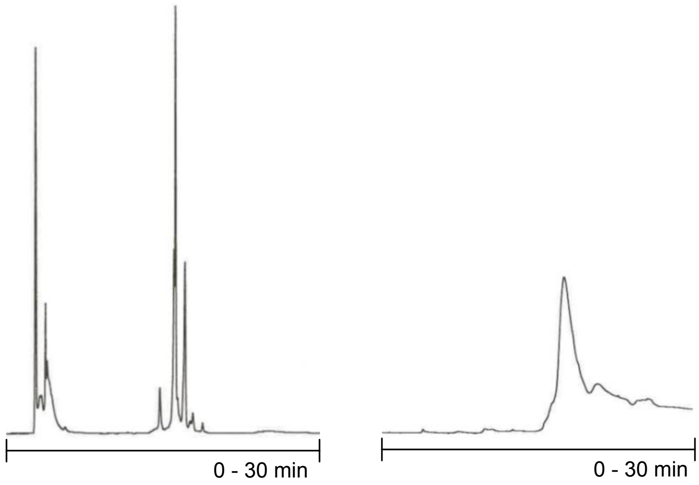

Figure S3. RP-HPLC chromatograms of ON(2) crude product with EDTA (left) and without EDTA (right).

HPLC conditions: Hypersil ODS C18 column (250 x 4.6mm, 5µm), buffer A: 0.1 M triethylammonium acetate in water, buffer B: 0.1 M triethylammonium acetate in acetonitrile. A gradient elution A/B: from 95/5 to 65/35 (0 - 25 min), then from 65/35 to 0/100 (25 – 30 min), flow rate = 1.0 mL/min, l = 260 nm, tR = 17.0 min (**ON2**).

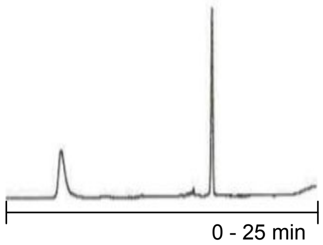

Figure S4. RP-HPLC chromatogram of purified ON(2).

HPLC conditions: Hypersil ODS C18 column (250 x 4,6mm, 5µm) flow rate 1 ml/min, l = 260 nm, buffer A: 0.1 M triethylammonium acetate in water, buffer B: 0.1 M triethylammonium acetate in acetonitrile, A gradient elution A/B: from 95/5 to 65/35 (0 – 25 min) flow rate = 1.0 mL/min, I = 260 nm, tR = 17.0 min (**ON2**).

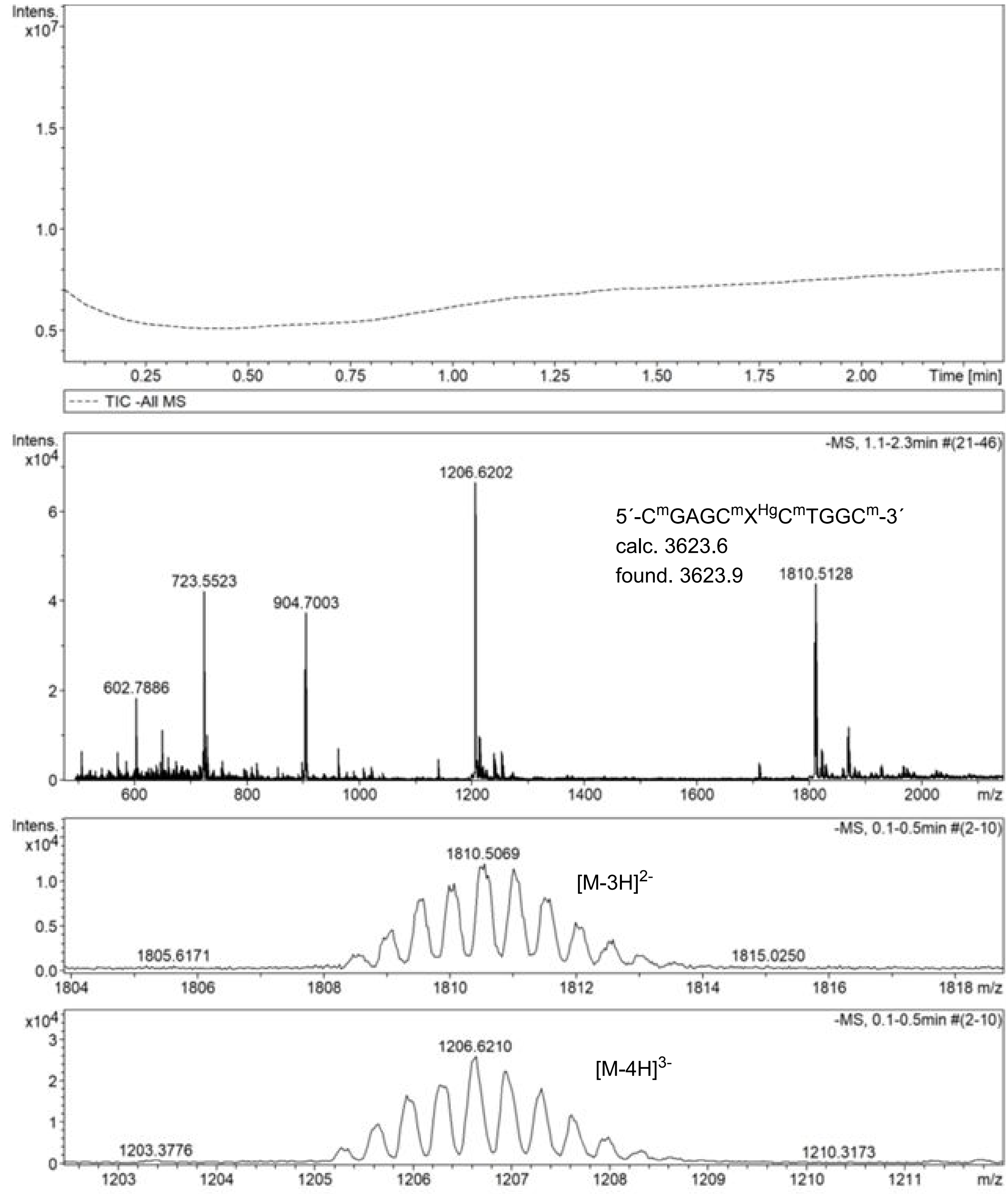

Figure S5. MS spectrum of ON(2)

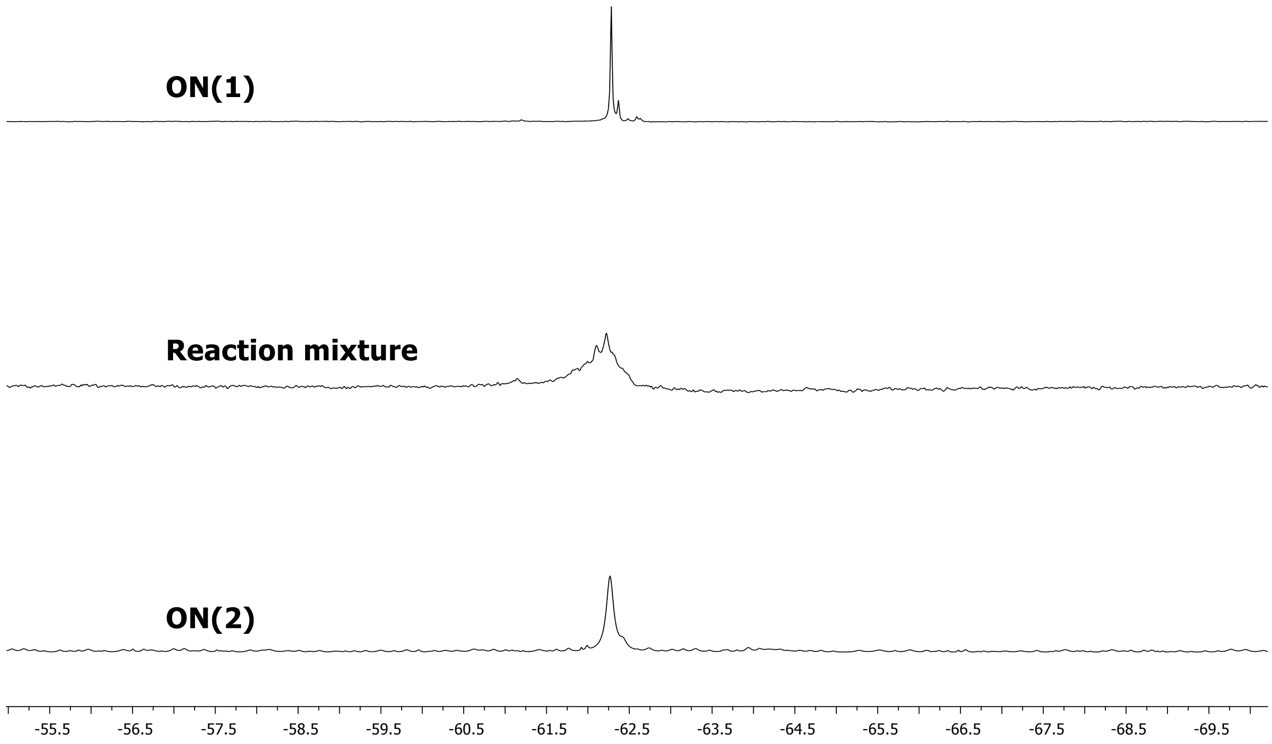

Figure S6. ^{19}F NMR spectra of ON(1), mercuration reaction mixture and ON(2).
(564 MHz, D_2O–H_2O, 1:9 *v/v*)

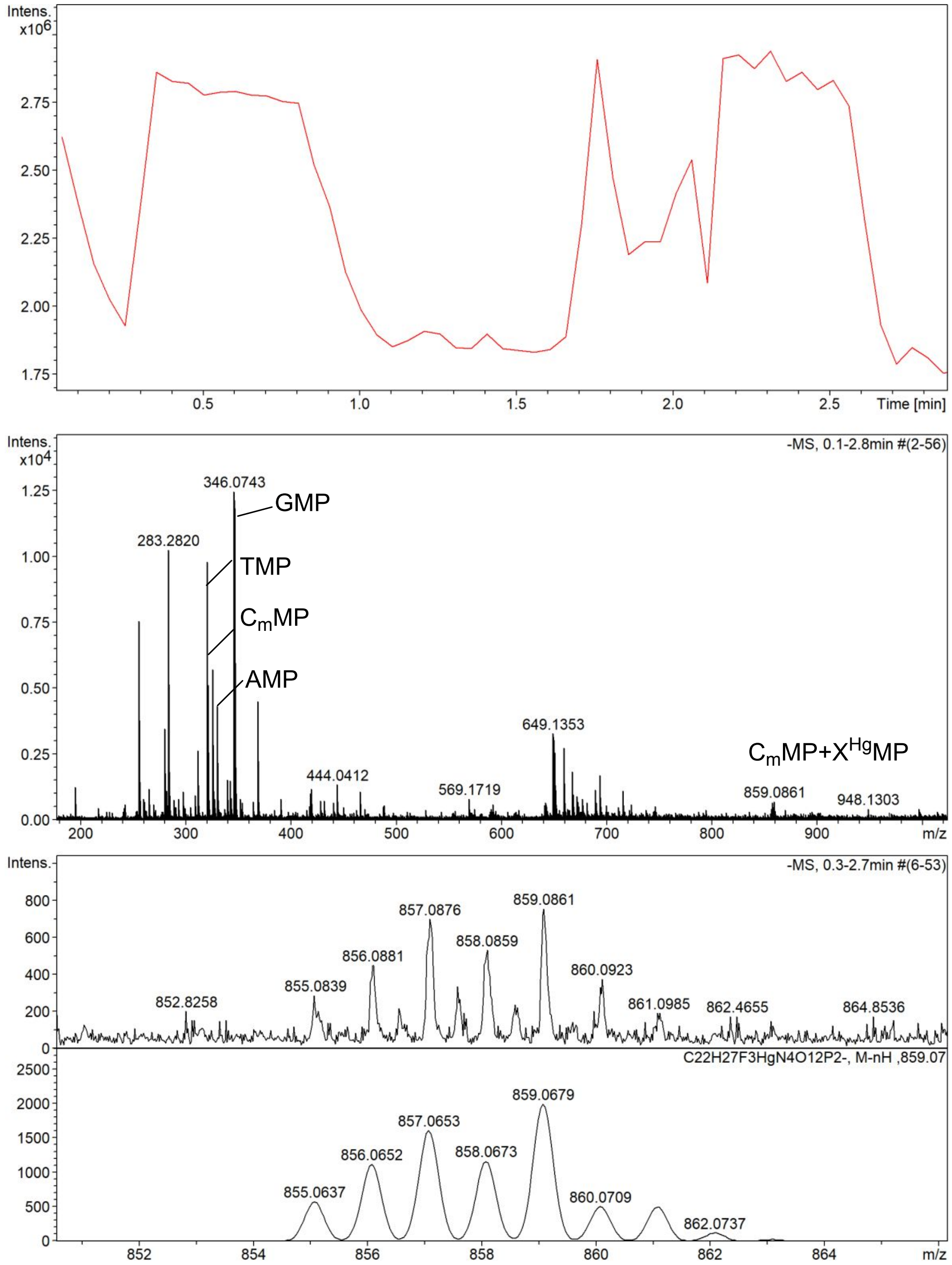

Figure S7. MS spectrum after digestion of P1 nuclease

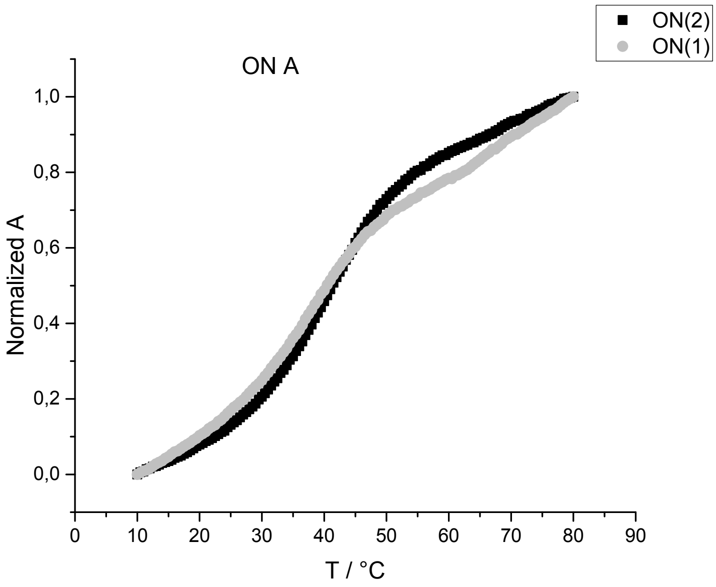

ON(2)
ON(1)
ON A
Normalized A
1,0
0,8
0,6
0,4
0,2
0,0
0
10
20
30
40
50
60
70
80
90
T / °C

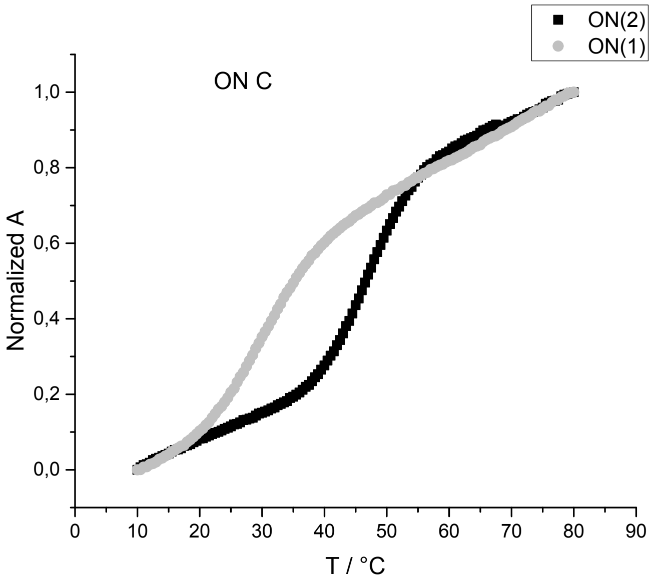

ON(2)
ON(1)
ON C
Normalized A
1,0
0,8
0,6
0,4
0,2
0,0
0
10
20
30
40
50
60
70
80
90
T / °C

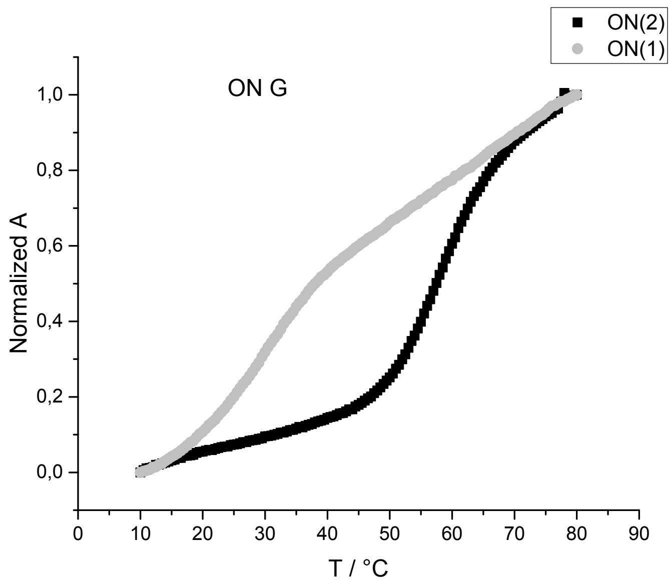

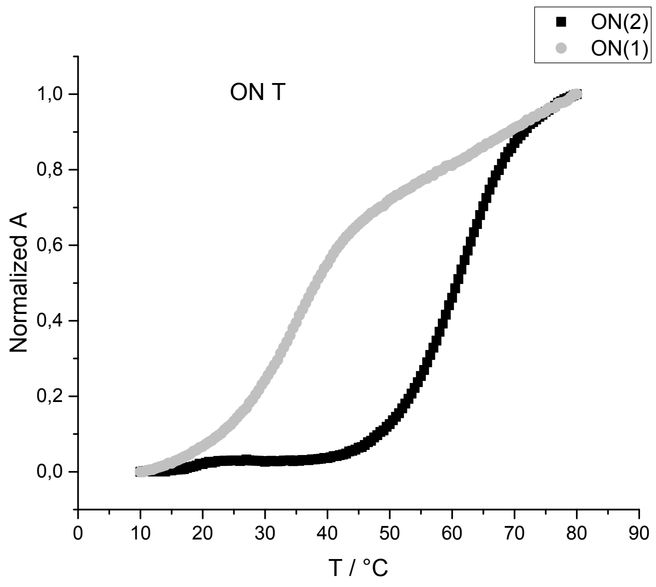

Figure S8. UV melting profiles of ON(2)•ON(Y) (black) and ON(1)•ON(Y) (grey).
2.0 umol L^{-1} of ON in 10 mmol L^{-1} cacodylate buffer (pH 7.0, *I* = 0.10 M adjusted with NaCl)

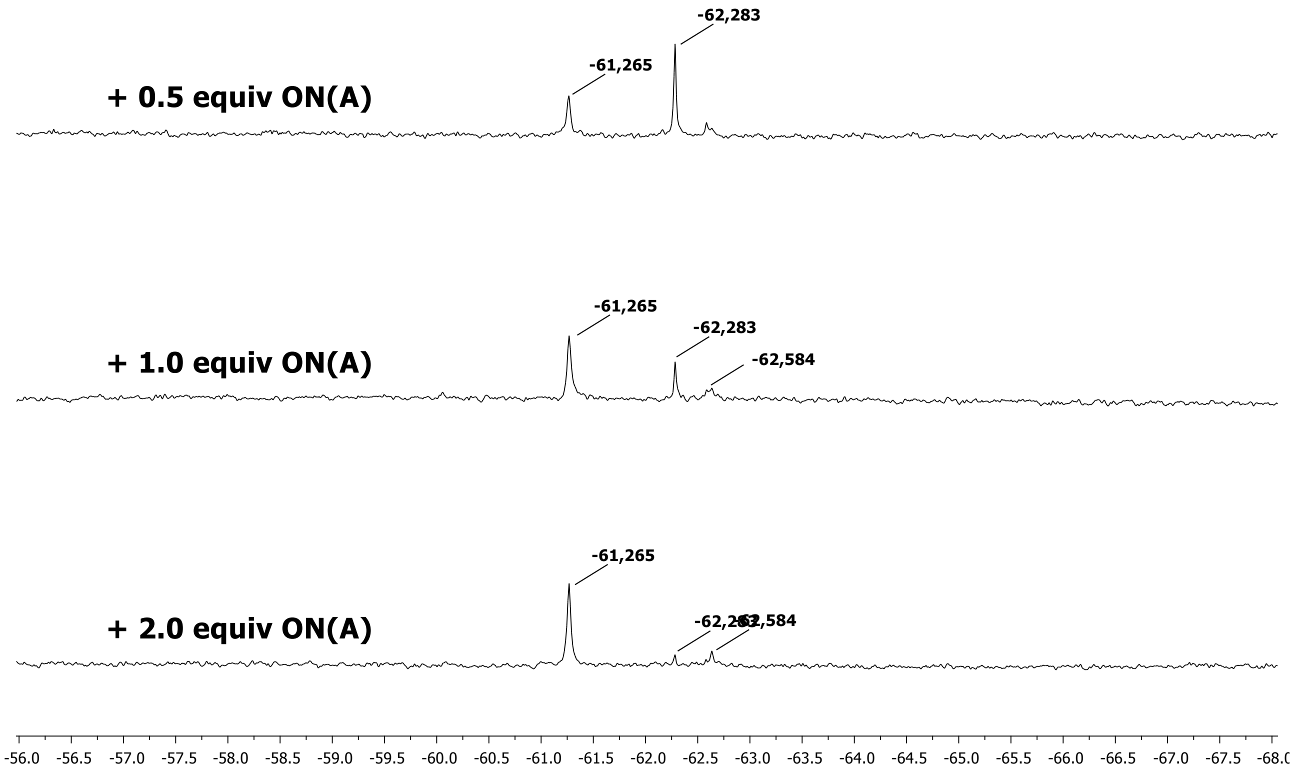

Figure S9. ^{19}F NMR spectra of ON(1)•ON(A)

Sample composition: 5 μM **ON(1)** in 10 mM cacodylate buffer (pH = 7.0, D_2O–H_2O, 1:9, *v/v*, *I* = 0.1 M adjusted with NaCl) at 25 °C. Number of scans were 2048. ^{19}F NMR frequency is 564 MHz.

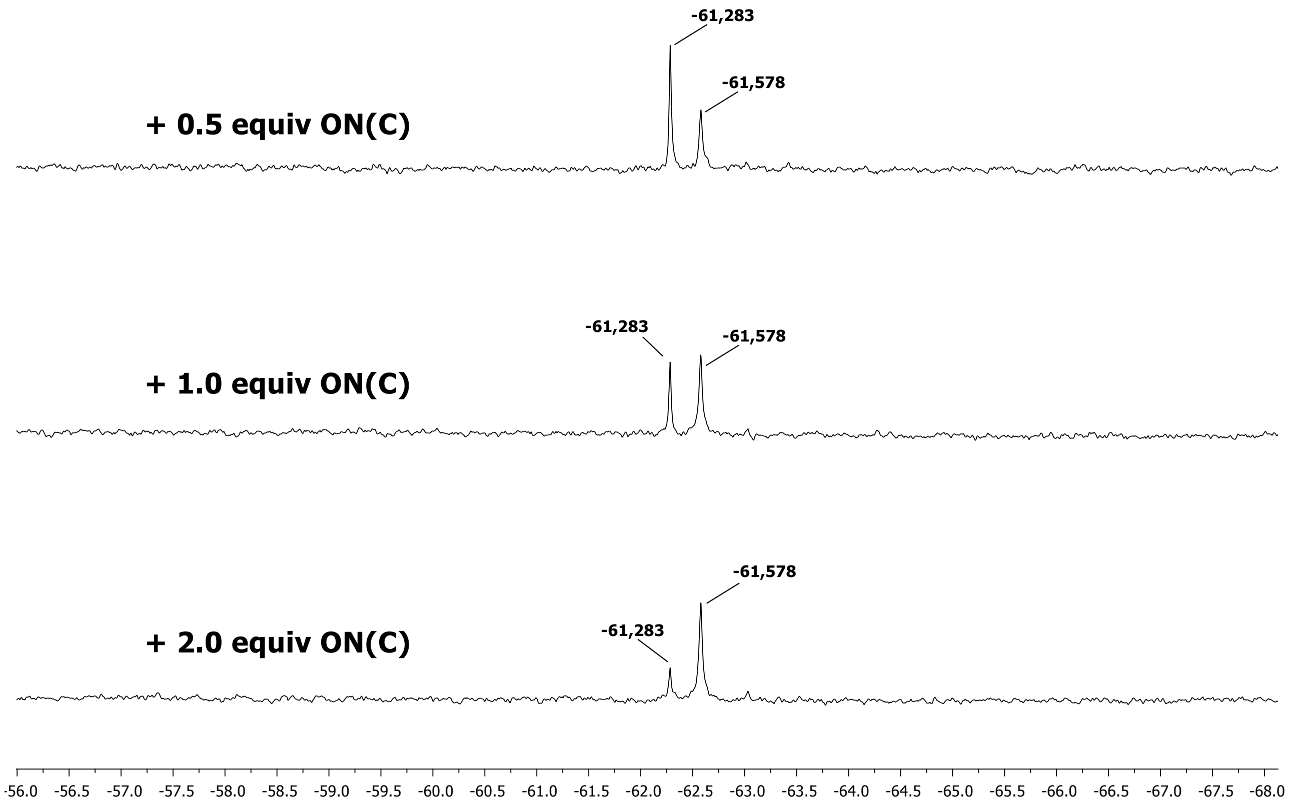

Figure S10. ^{19}F NMR spectra of ON(1)•ON(C)

Sample composition: 5 µM **ON(1)** in 10 mM cacodylate buffer (pH = 7.0, D_2O−H_2O, 1:9, *v/v*, *I* = 0.1 M adjusted with NaCl) at 25 °C. Number of scans were 2048. ^{19}F NMR frequency is 564 MHz.

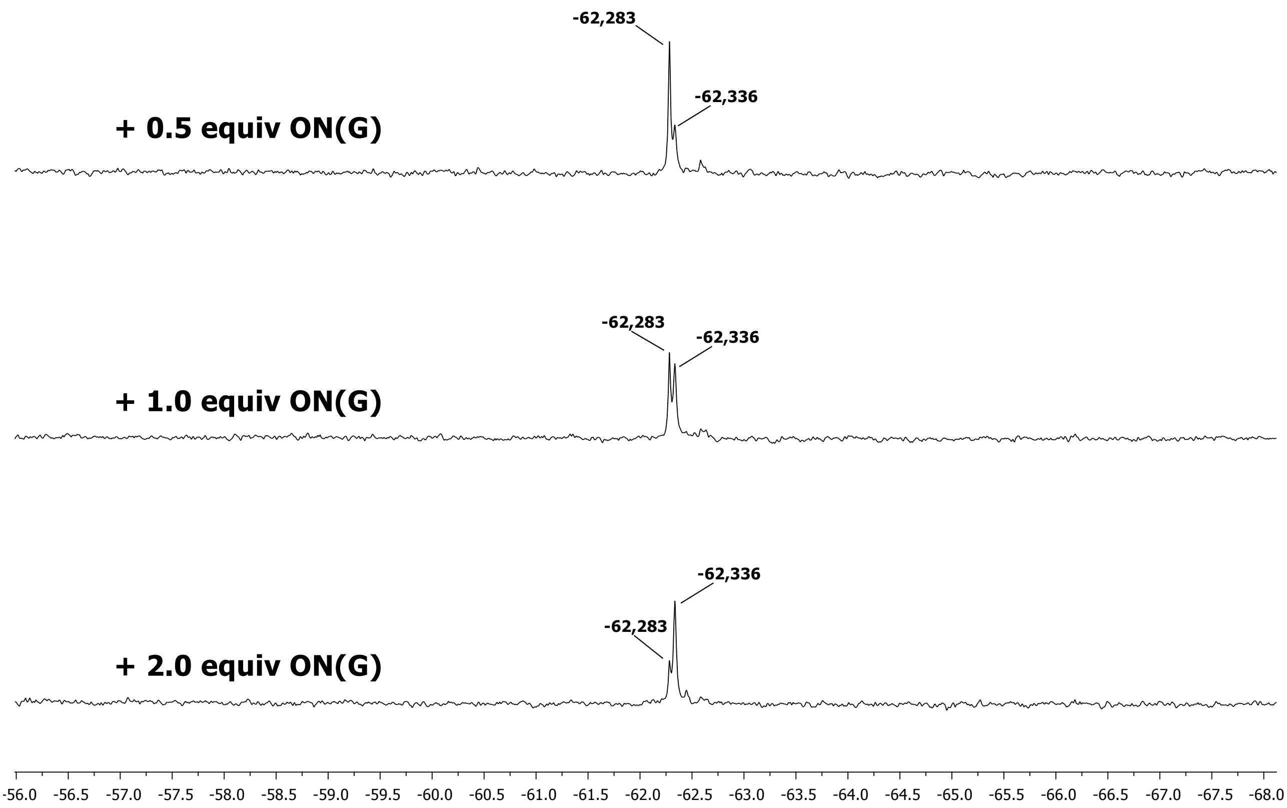

Figure S11. ^{19}F NMR spectra of ON(1)•ON(G)

Sample composition: 5 µM **ON(1)** in 10 mM cacodylate buffer (pH = 7.0, D_2O–H_2O, 1:9, *v/v*, *I* = 0.1 M adjusted with NaCl) at 25 °C. Number of scans were 2048. ^{19}F NMR frequency is 564 MHz.

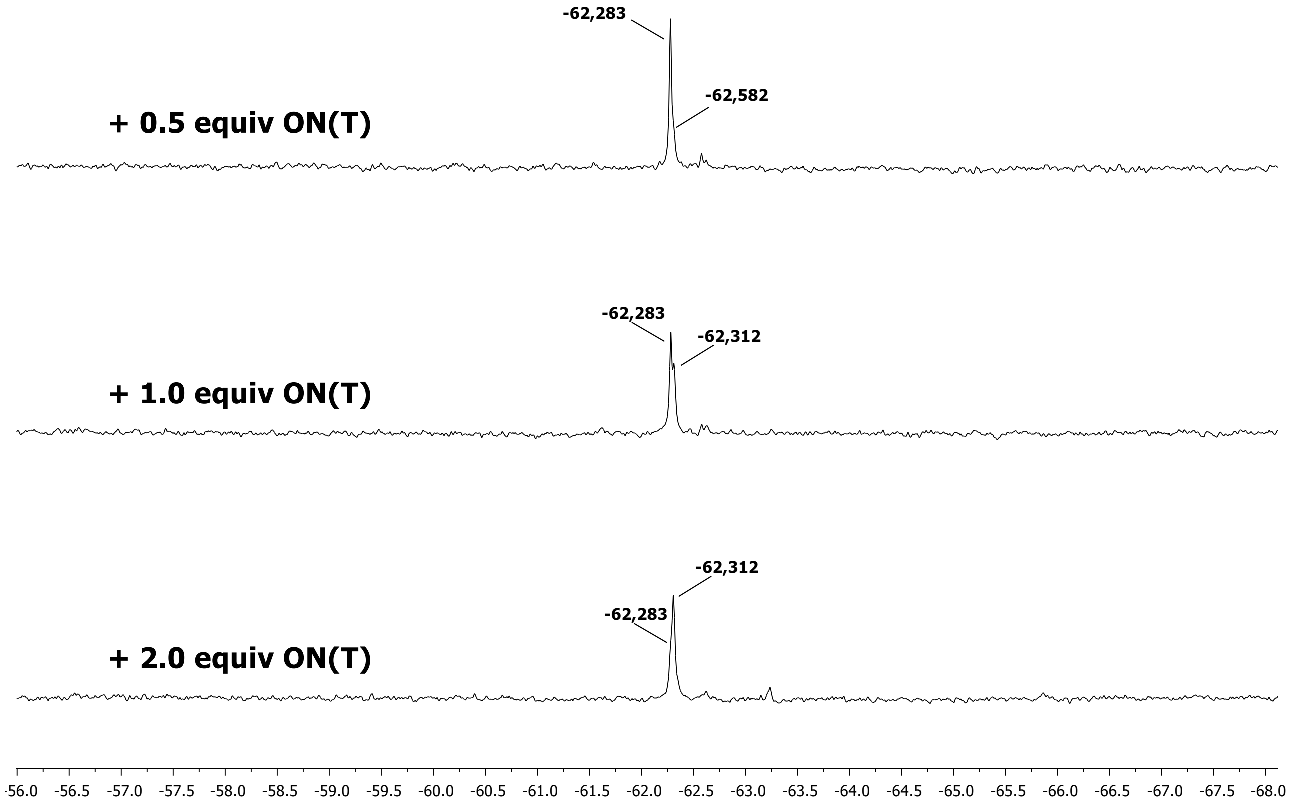

Figure S12. ^{19}F NMR spectra of ON(1)•ON(T)

Sample composition: 5 µM **ON(1)** in 10 mM cacodylate buffer (pH = 7.0, D_2O–H_2O, 1:9, *v/v*, *I* = 0.1 M adjusted with NaCl) at 25 °C. Number of scans were 2048. ^{19}F NMR frequency is 564 MHz.

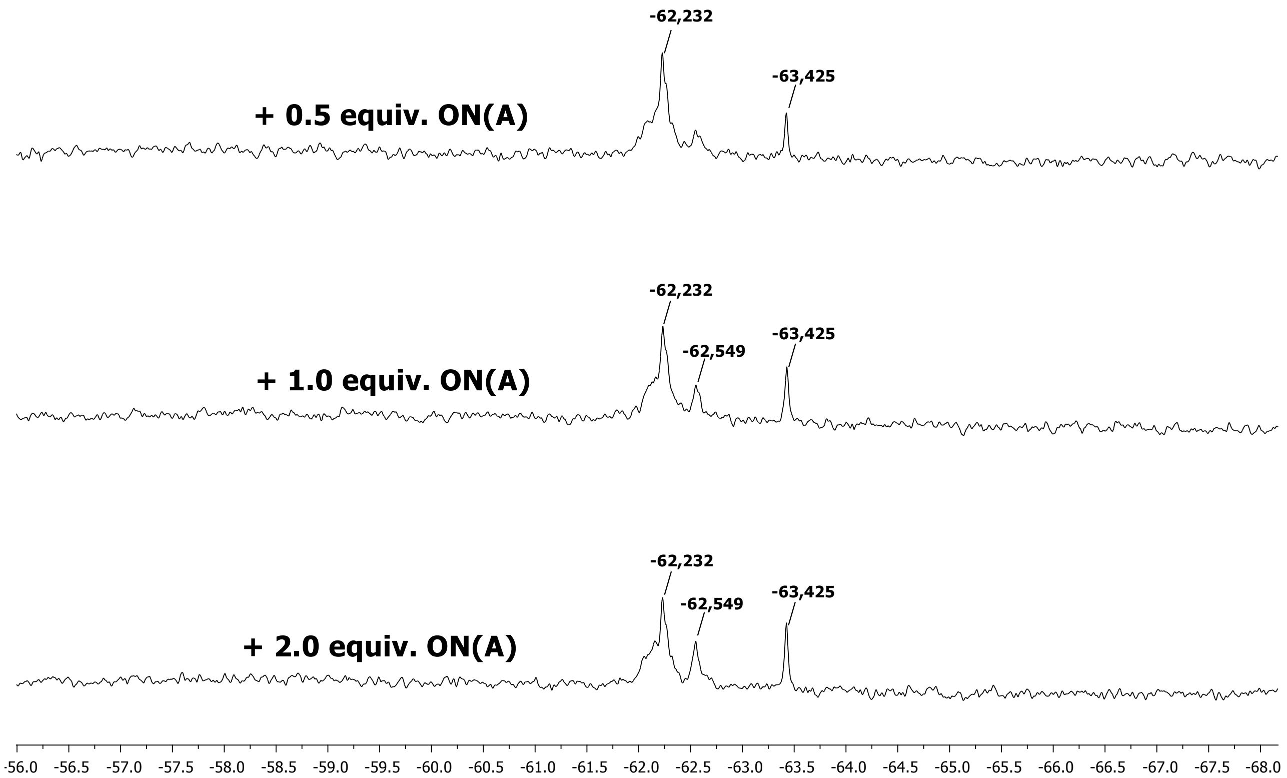

Figure S13. ^{19}F NMR spectra of ON(2)•ON(A)

Sample composition: 10 µM **ON(2)** in 10 mM cacodylate buffer (pH = 7.0, D_2O−H_2O, 1:9, *v/v*, *I* = 0.1 M adjusted with NaCl) at 25 °C. Number of scans were 2048. ^{19}F NMR frequency is 564 MHz.

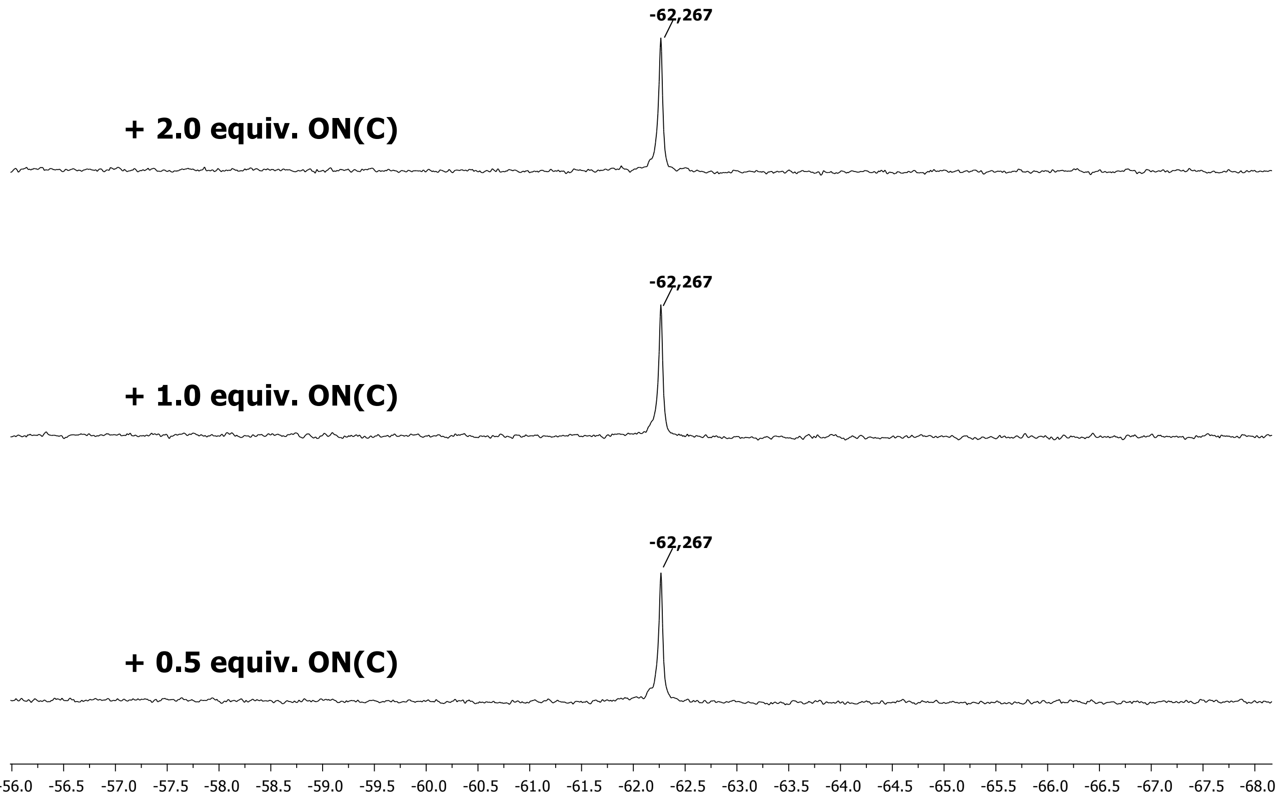

Figure S14. ^{19}F NMR spectra of ON(2)•ON(C)

Sample composition: 10 µM **ON(2)** in 10 mM cacodylate buffer (pH = 7.0, D_2O−H_2O, 1:9, *v/v*, *I* = 0.1 M adjusted with NaCl) at 25 °C. Number of scans were 2048. ^{19}F NMR frequency is 564 MHz.

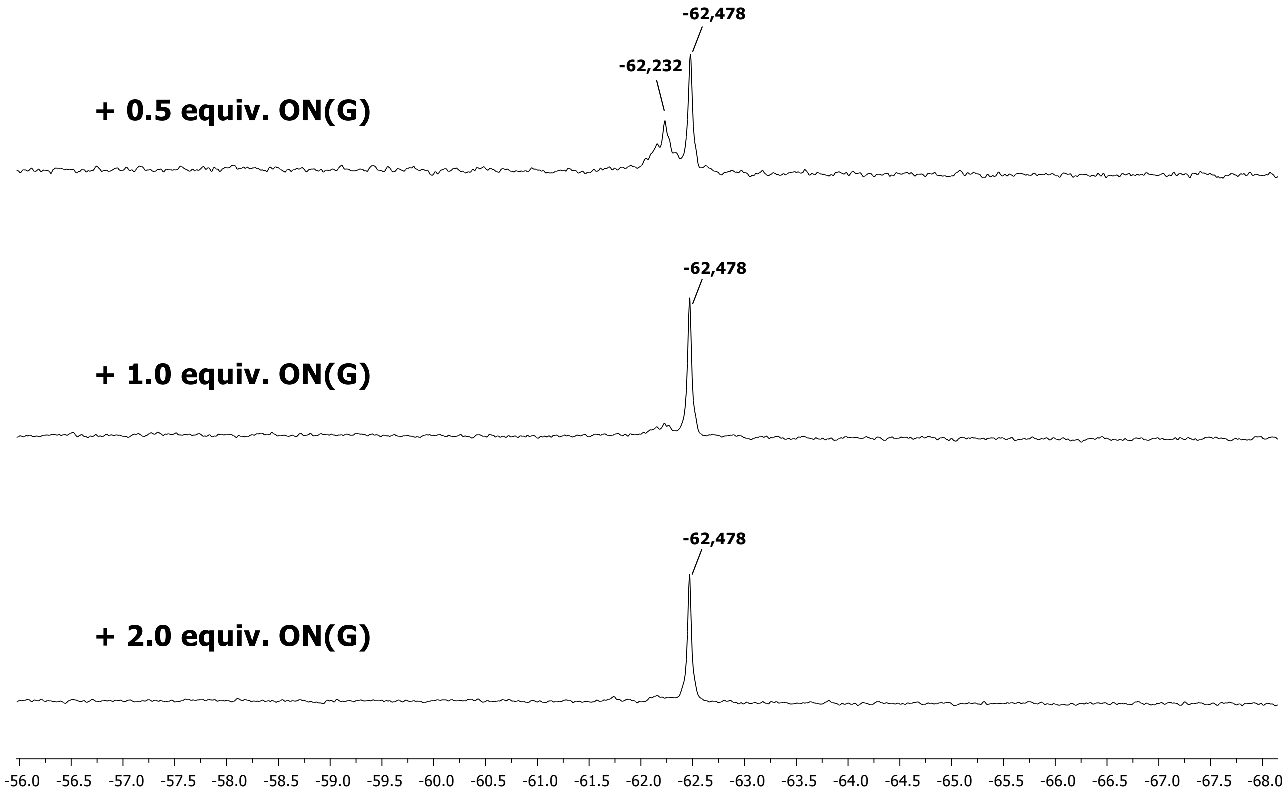

Figure S15. ^{19}F NMR spectra of ON(2)•ON(G)

Sample composition: 10 µM **ON(2)** in 10 mM cacodylate buffer (pH = 7.0, D_2O−H_2O, 1:9, *v/v*, *I* = 0.1 M adjusted with NaCl) at 25 °C. Number of scans were 2048. ^{19}F NMR frequency is 564 MHz.

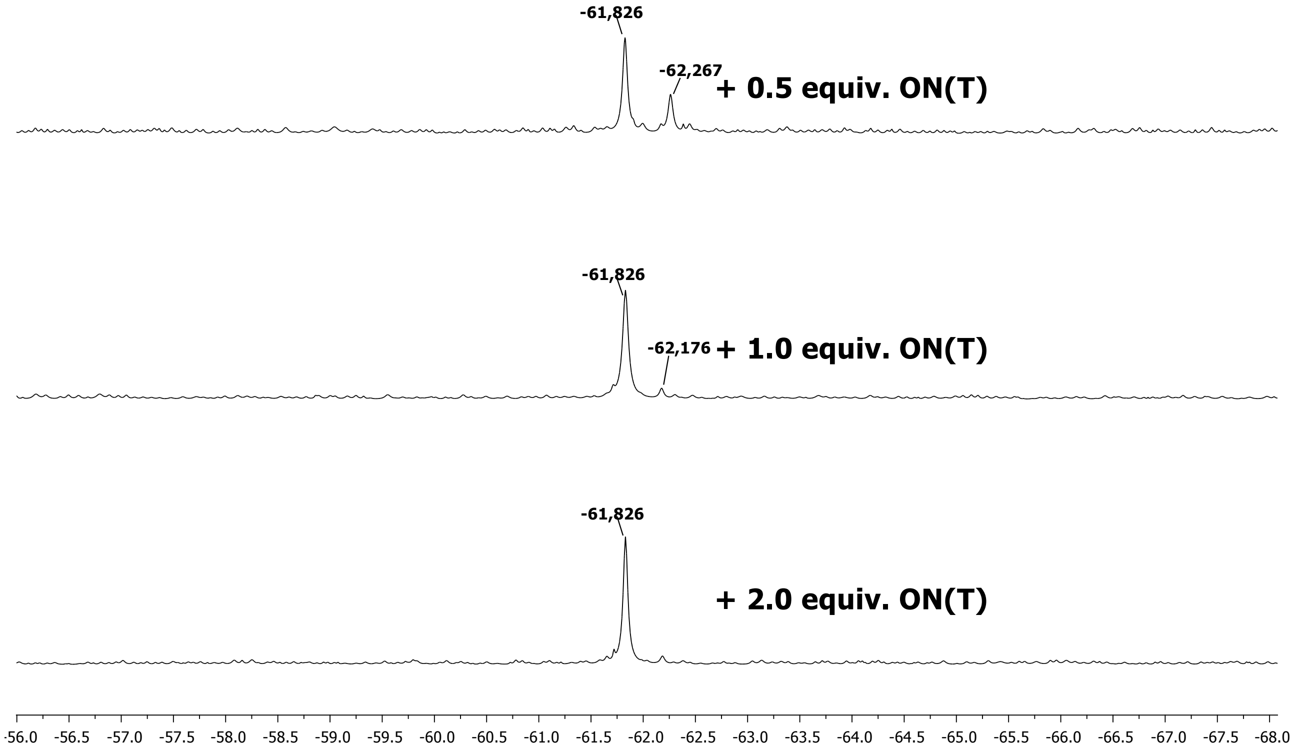

Figure S16. ^{19}F NMR spectra of ON(2)•ON(T)

Sample composition: 10 µM **ON(2)** in 10 mM cacodylate buffer (pH = 7.0, D_2O−H_2O, 1:9, *v/v*, *I* = 0.1 M adjusted with NaCl) at 25 °C. Number of scans were 2048. ^{19}F NMR frequency is 564 MHz.

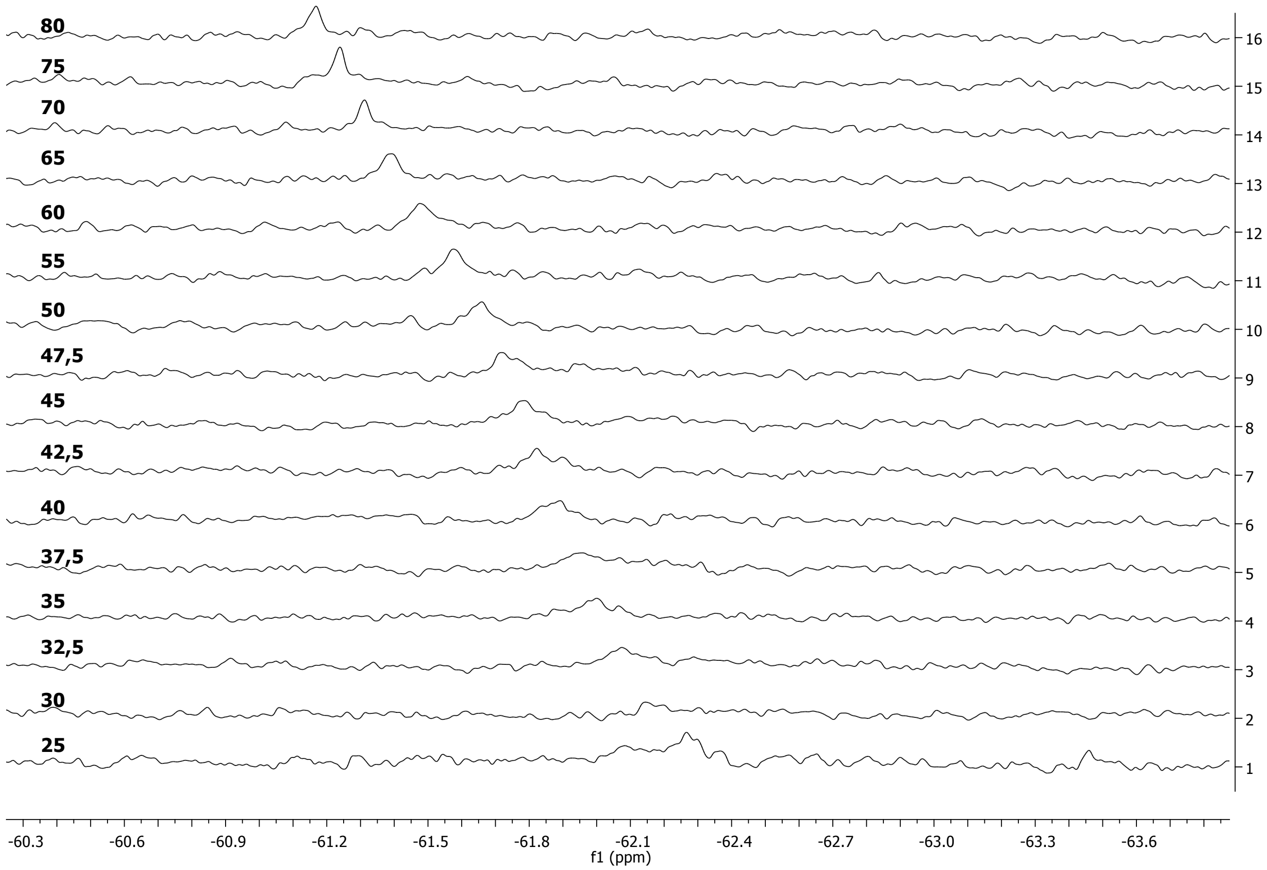

Figure S17. ^{19}**F NMR temperature ramp of ON(2)•ON(A)**

Sample composition: 10 µM **ON(2)** in 10 mM cacodylate buffer (pH = 7.0, D_2O−H_2O, 1:9, *v/v*, *I* = 0.1 M adjusted with NaCl). Number of scans were 2048. Temperature range between 25−70 °C, 2.5 or 5 °C intervals, from bottom to top. ^{19}F NMR frequency is 564 MHz.

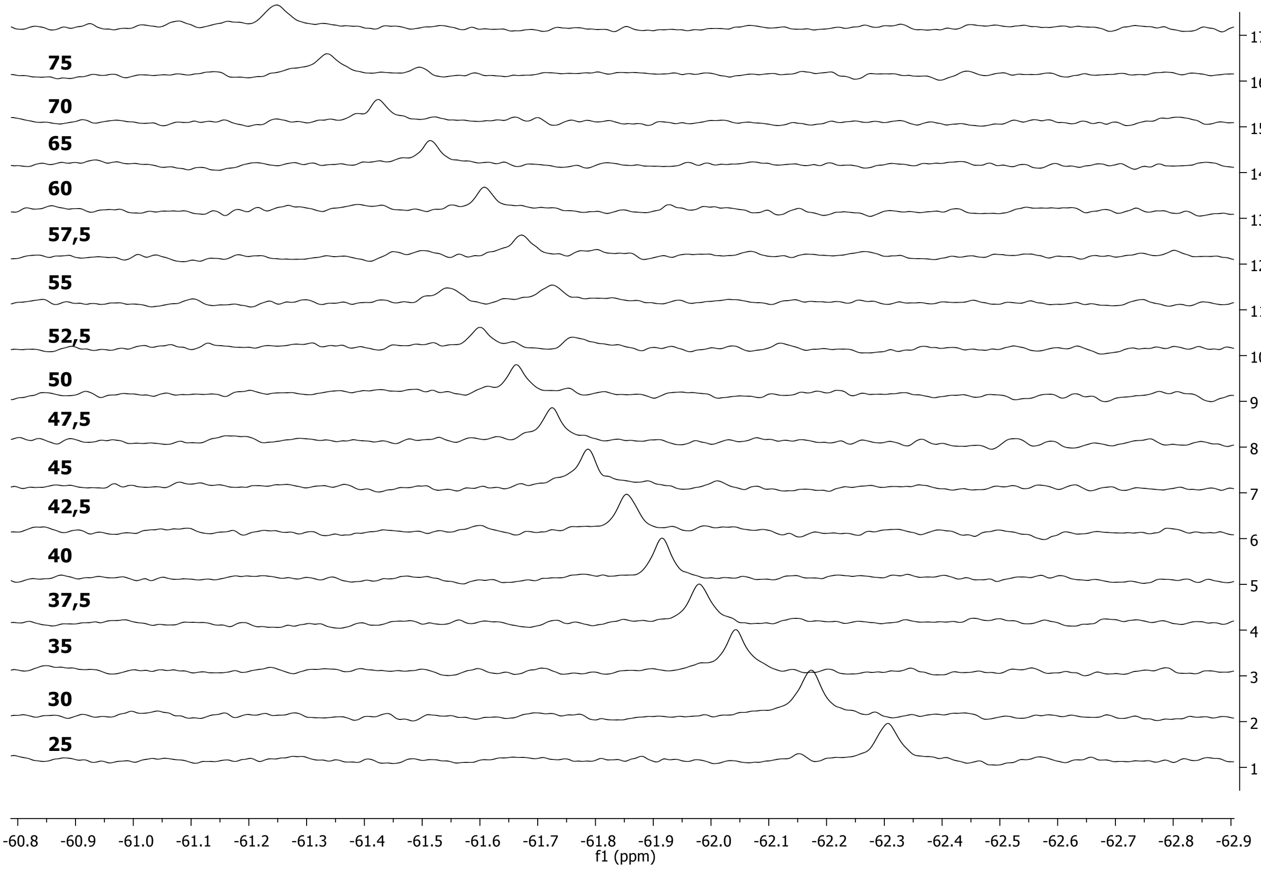

Figure S18. ^{19}F NMR temperature ramp of ON(2)•ON(C)

Sample composition: 10 µM **ON(2)** in 10 mM cacodylate buffer (pH = 7.0, D_2O–H_2O, 1:9, *v/v*, *I* = 0.1 M adjusted with NaCl). Number of scans were 2048. Temperature range between 25−80 °C, 2.5 or 5 °C intervals, from bottom to top. ^{19}F NMR frequency is 564 MHz.

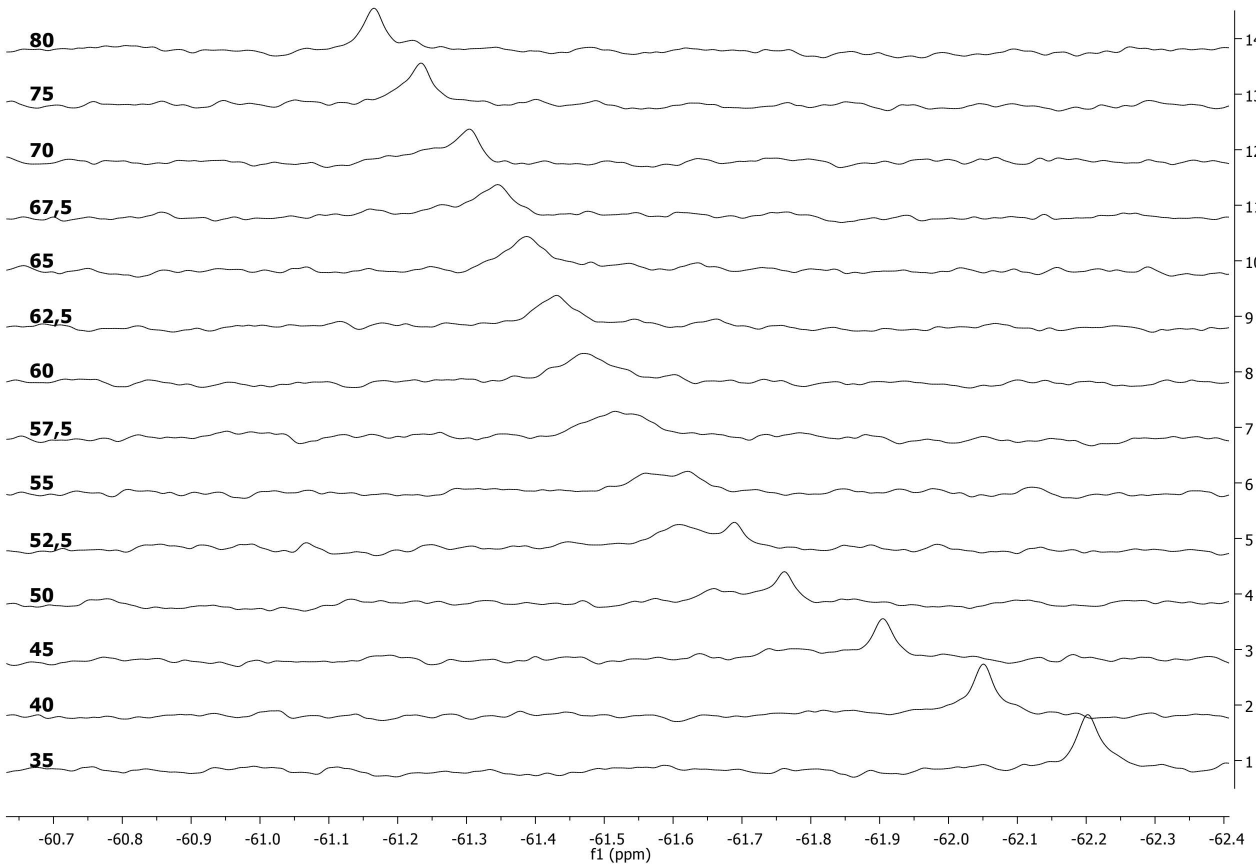

Figure S19. ^{19}**F NMR temperature ramp of ON(2)•ON(G)**

Sample composition: 10 µM **ON(2)** in 10 mM cacodylate buffer (pH = 7.0, D_2O–H_2O, 1:9, *v/v*, *I* = 0.1 M adjusted with NaCl). Number of scans were 2048. Temperature range between 35−80 °C, 2.5 or 5 °C intervals, from bottom to top. ^{19}F NMR frequency is 564 MHz.

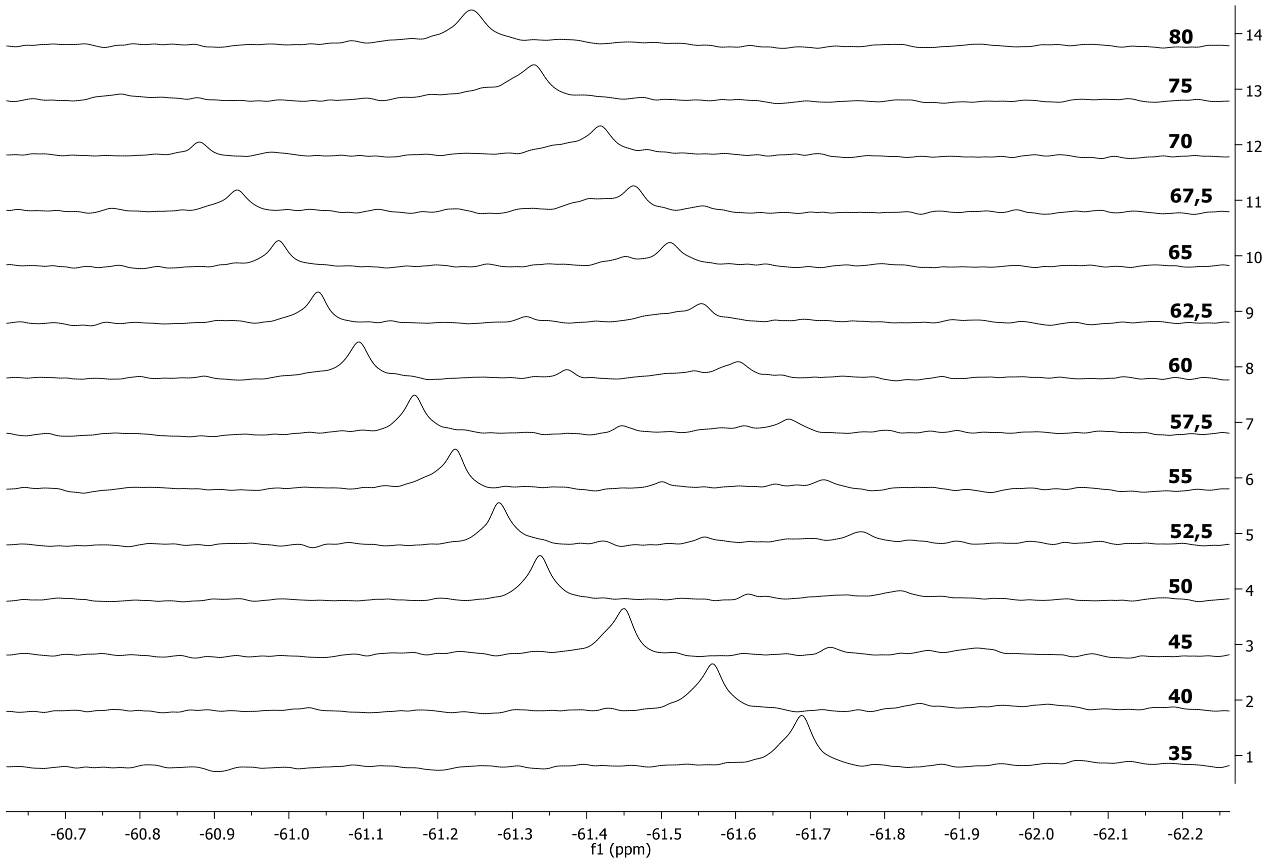

Figure S20. ^{19}F NMR temperature ramp of ON(2)•ON(T)

Sample composition: 10 µM **ON(2)** in 10 mM cacodylate buffer (pH = 7.0, D_2O–H_2O, 1:9, *v/v*, *I* = 0.1 M adjusted with NaCl). Number of scans were 2048. Temperature range between 25−80 °C, 2.5 or 5 °C intervals, from bottom to top. ^{19}F NMR frequency is 564 MHz.

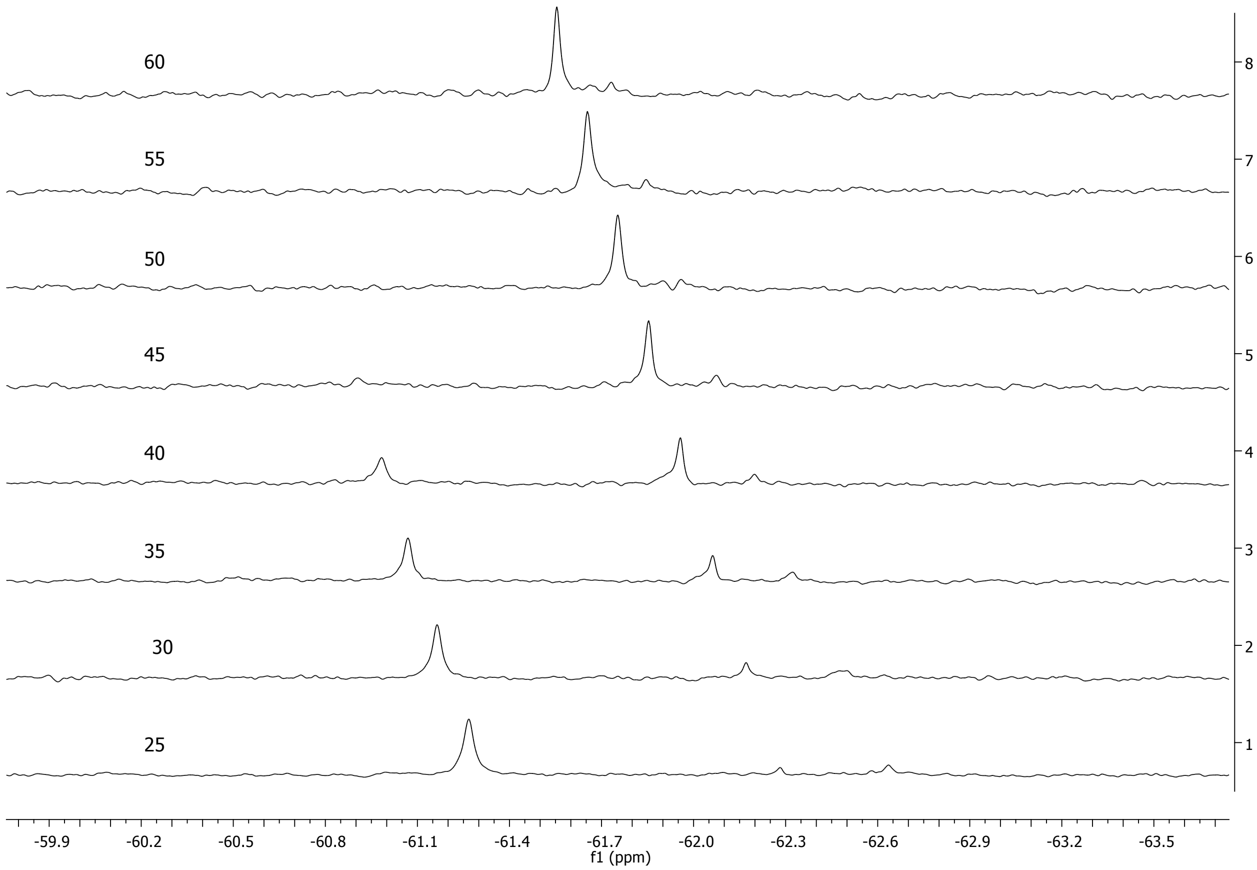

Figure S21. ^{19}F NMR temperature ramp of ON(1)•ON(A)

Sample composition: 5 µM **ON(1)** in 10 mM cacodylate buffer (pH = 7.0, D_2O−H_2O, 1:9, *v/v*, *I* = 0.1 M adjusted with NaCl). Number of scans were 2048. Temperature range between 25−60 °C, 5 °C intervals, from bottom to top. ^{19}F NMR frequency is 564 MHz.

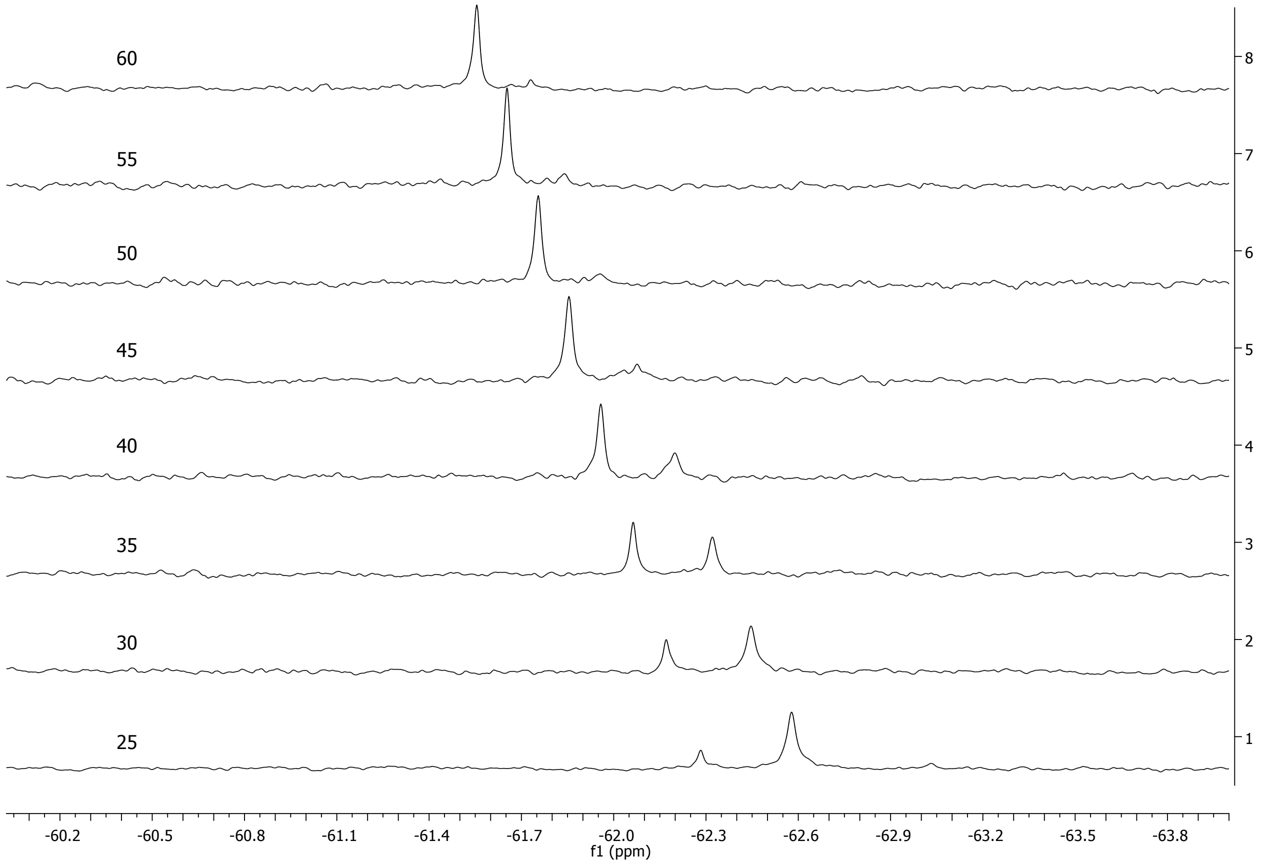

Figure S22. ^{19}F NMR temperature ramp of ON(1)•ON(C)

Sample composition: 5 µM **ON(1)** in 10 mM cacodylate buffer (pH = 7.0, D_2O−H_2O, 1:9, *v/v*, *I* = 0.1 M adjusted with NaCl). Number of scans were 2048. Temperature range between 25−60 °C, 5 °C intervals, from bottom to top. ^{19}F NMR frequency is 564 MHz.

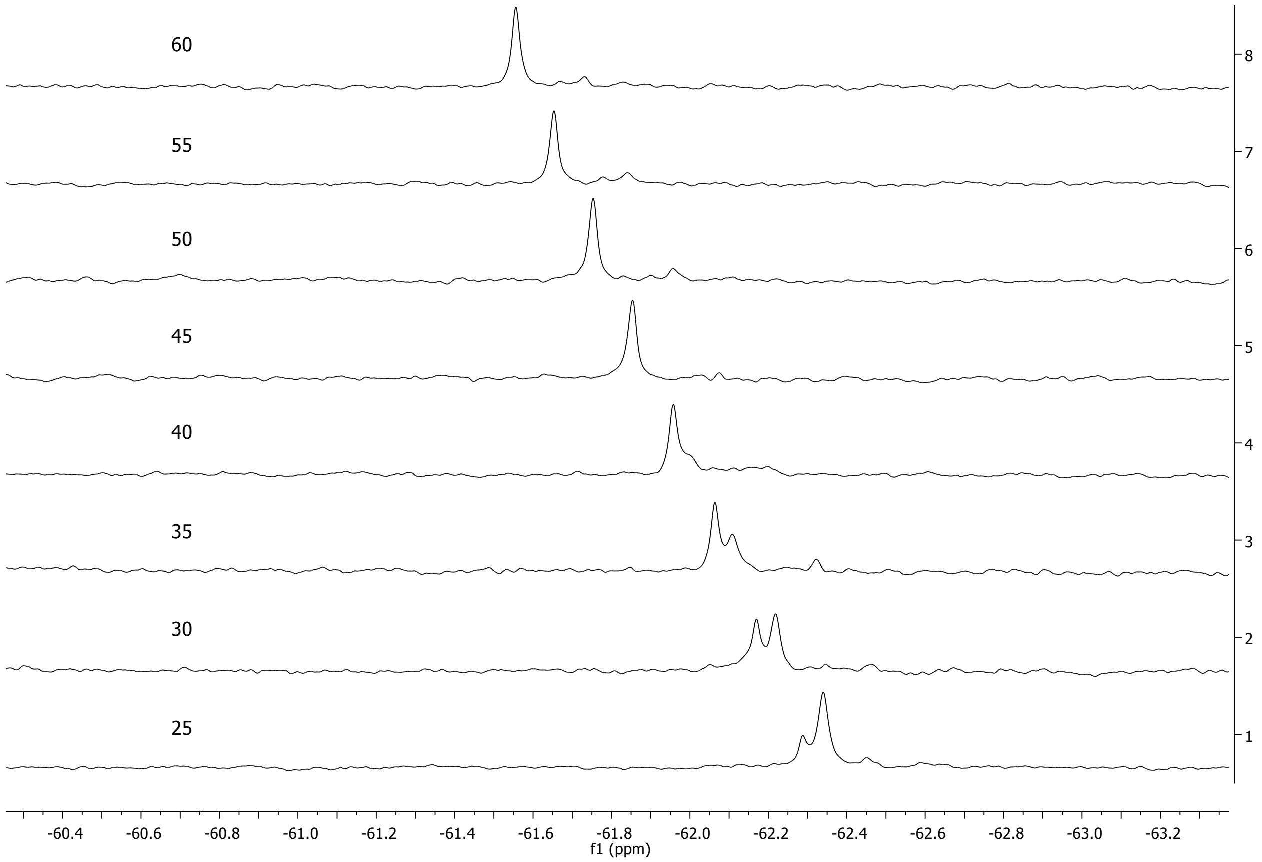

Figure S23. ^{19}F NMR temperature ramp of ON(1)•ON(G)

Sample composition: 5 µM **ON(1)** in 10 mM cacodylate buffer (pH = 7.0, D_2O−H_2O, 1:9, *v/v*, *I* = 0.1 M adjusted with NaCl). Number of scans were 2048. Temperature range between 25−60 °C, 5 °C intervals, from bottom to top. ^{19}F NMR frequency is 564 MHz.

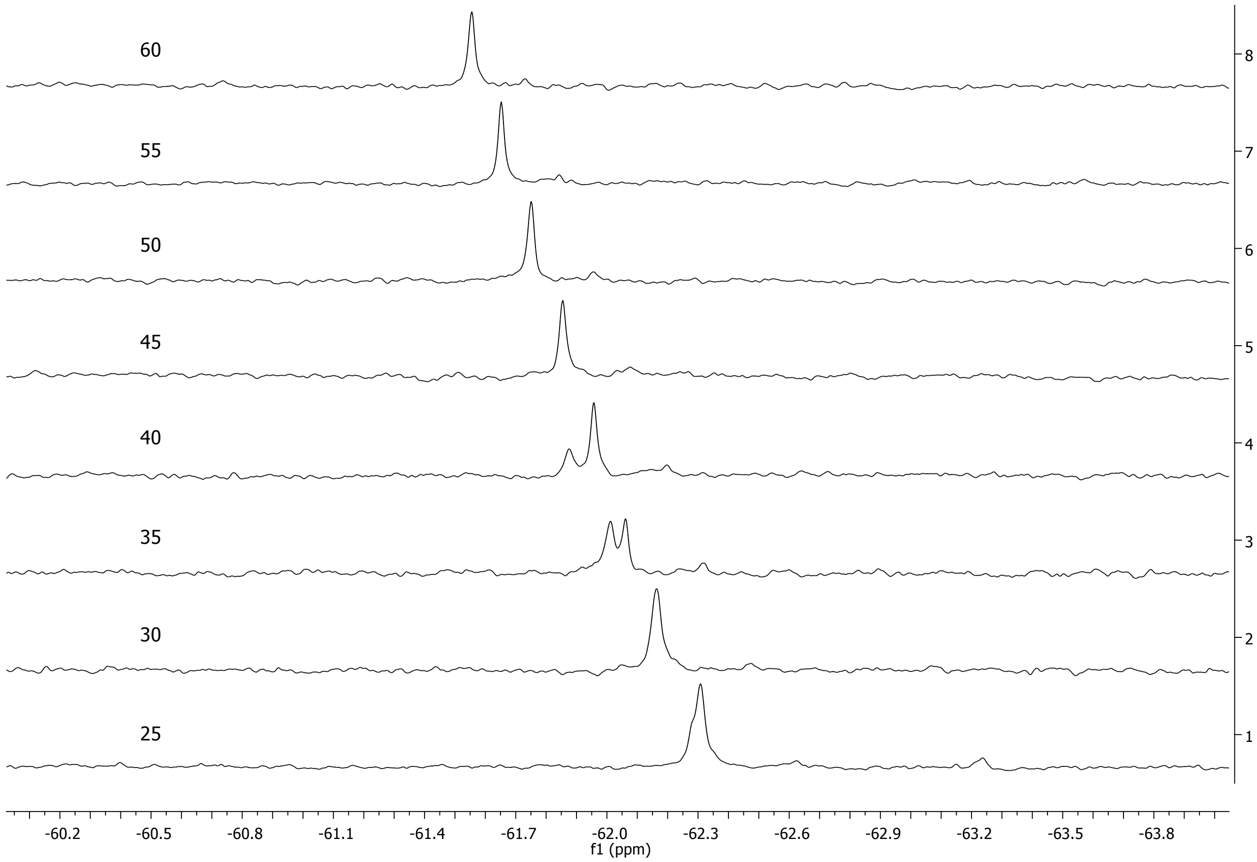

Figure S24. ^{19}F NMR temperature ramp of ON(1)•ON(T)
Sample composition: 5 µM **ON(1)** in 10 mM cacodylate buffer (pH = 7.0, D_2O−H_2O, 1:9, *v/v*, *I* = 0.1 M adjusted with NaCl). Number of scans were 2048. Temperature range between 25−60 °C, 5 °C intervals, from bottom to top. ^{19}F NMR frequency is 564 MHz.

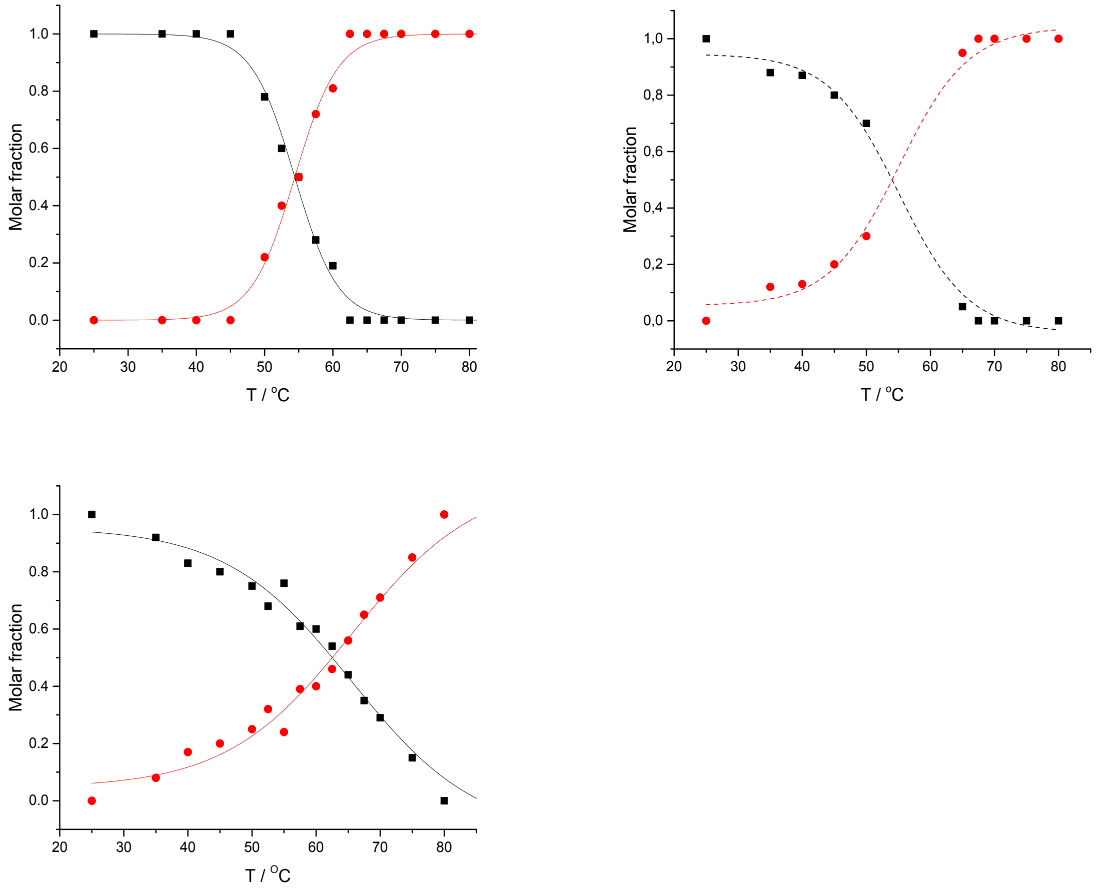

Figure S25. ^{19}F NMR melting curves of ON(2)•ON(Y)
ON(2)•ON(C) (left), ON(2)•ON(G) (right) and ON(2)•ON(T) (down)

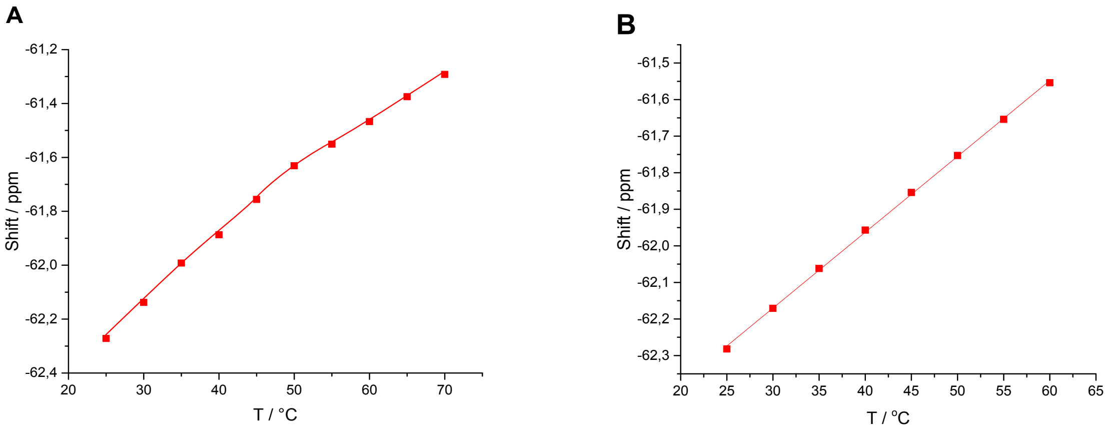

Figure S26. Temperature dependance of ON(2) and ON(1) oligonucleotide.
A) **ssON(2)** and B) **ssON(1)**.

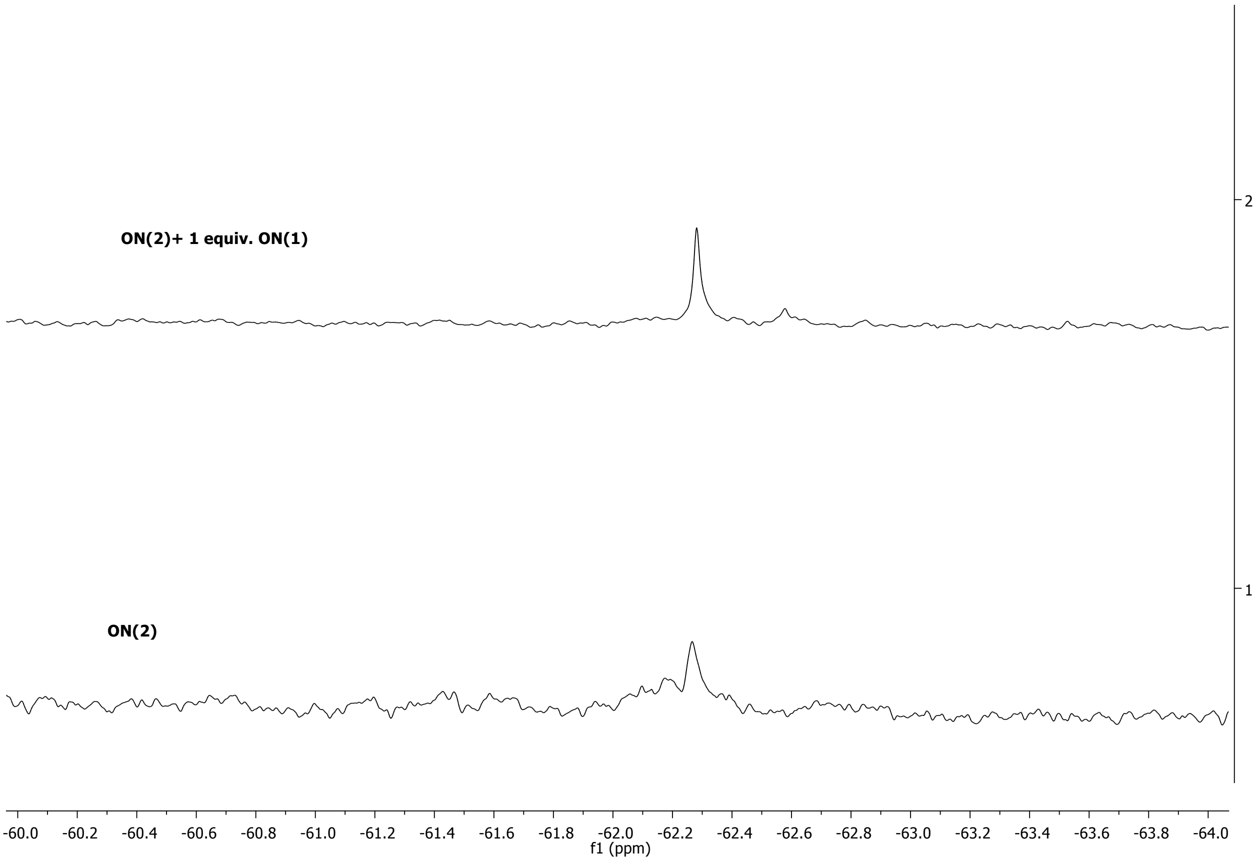

Figure S27. ^{19}F NMR spectra comparison of ON(2) and mixed ON(2)&ON(1).

Sample composition: 2 µM **ON(2)** in 10 mM cacodylate buffer (pH = 7.0, D_2O−H_2O, 1:9, *v/v*, *I* = 0.1 M adjusted with NaCl). Number of scans were 2048. ^{19}F NMR frequency is 564 MHz.

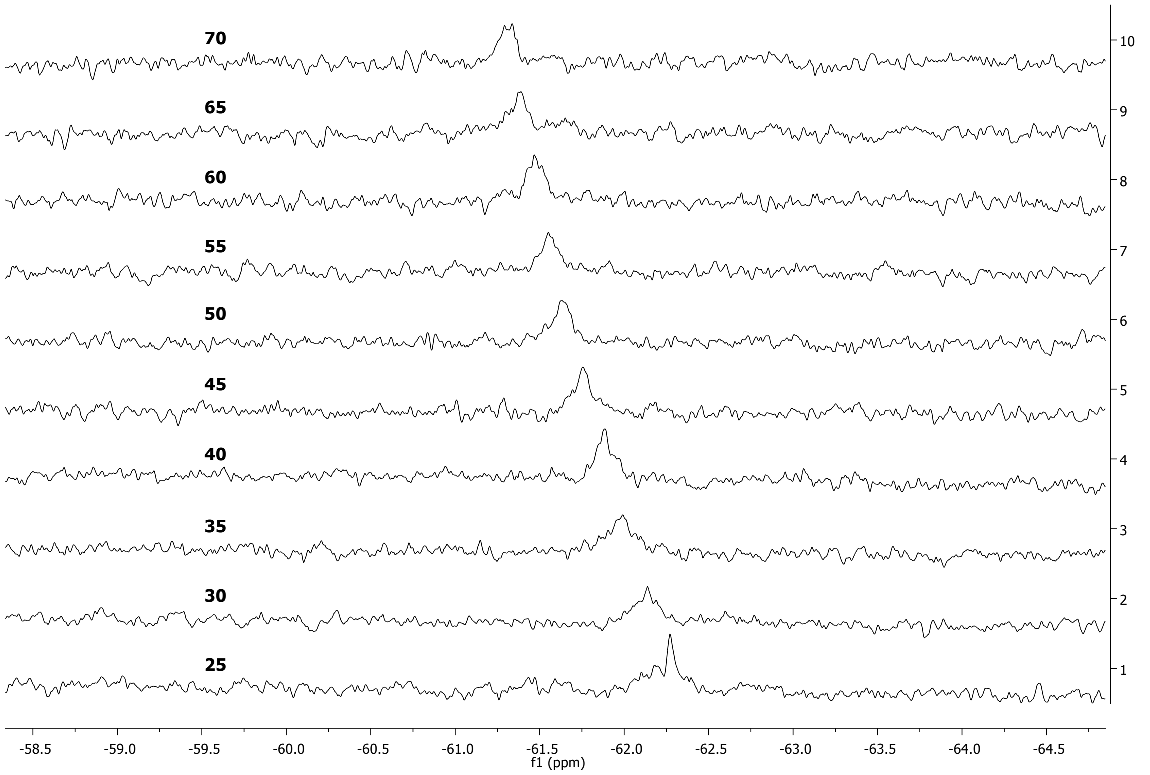

Figure S28. ^{19}F NMR temperature ramp of ssON(2)

Sample composition: 2 µM **ON(2)** in 10 mM cacodylate buffer (pH = 7.0, D_2O−H_2O, 1:9, *v/v*, *I* = 0.1 M adjusted with NaCl). Number of scans were 2048. Temperature range between 25−70 °C, 5 °C intervals, from bottom to top. ^{19}F NMR frequency is 564 MHz.

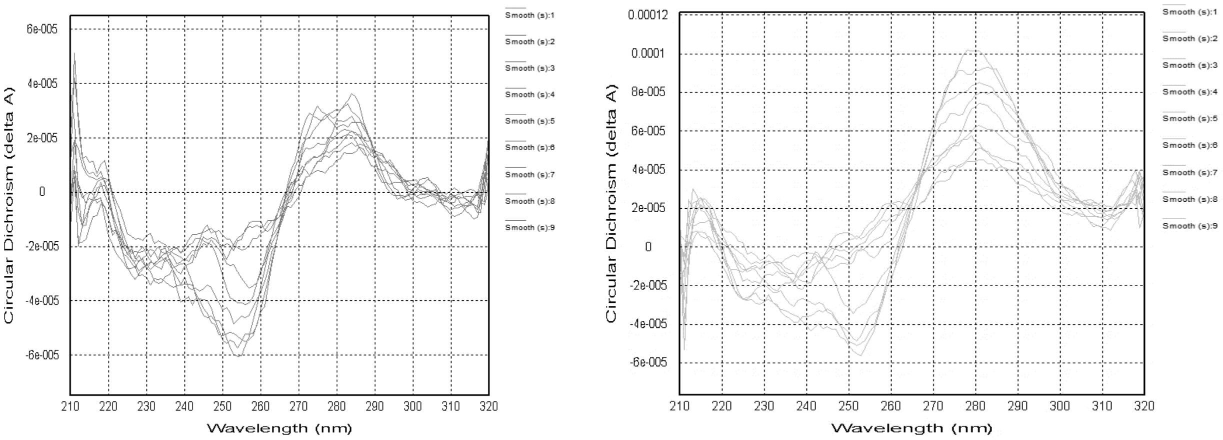

Figure S29. CD spectra: ON(2)•ON(A) (left) and ON(1)•ON(A) (right).

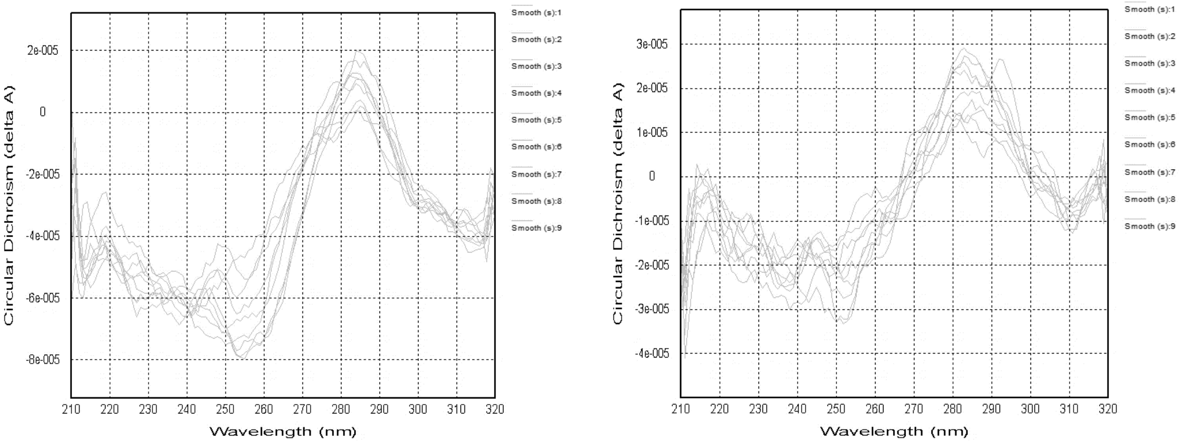

Figure S30. CD spectra: ON(2)•ON(C) (left) and ON(1)•ON(C) (right).

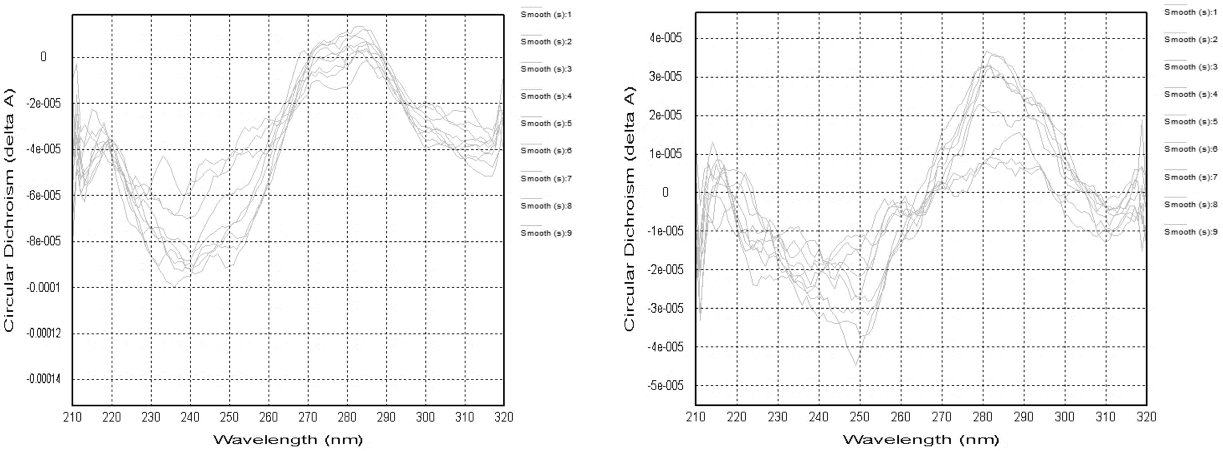

Figure S31. CD spectra: ON(2)•ON(G) (left) and ON(1)•ON(G) (right).

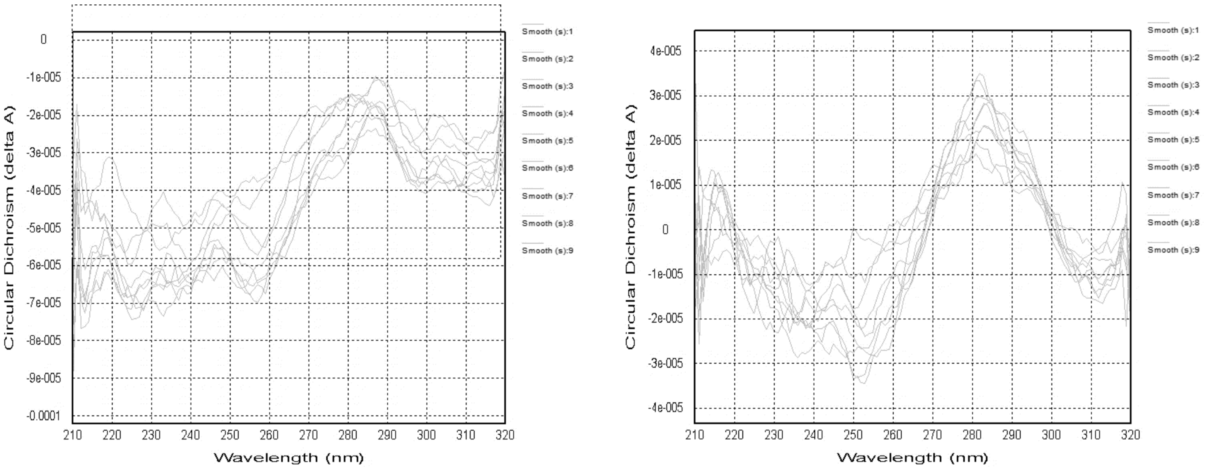

Figure S32. CD spectra: ON(2)•ON(T) (left) and ON(1)•ON(T) (right).

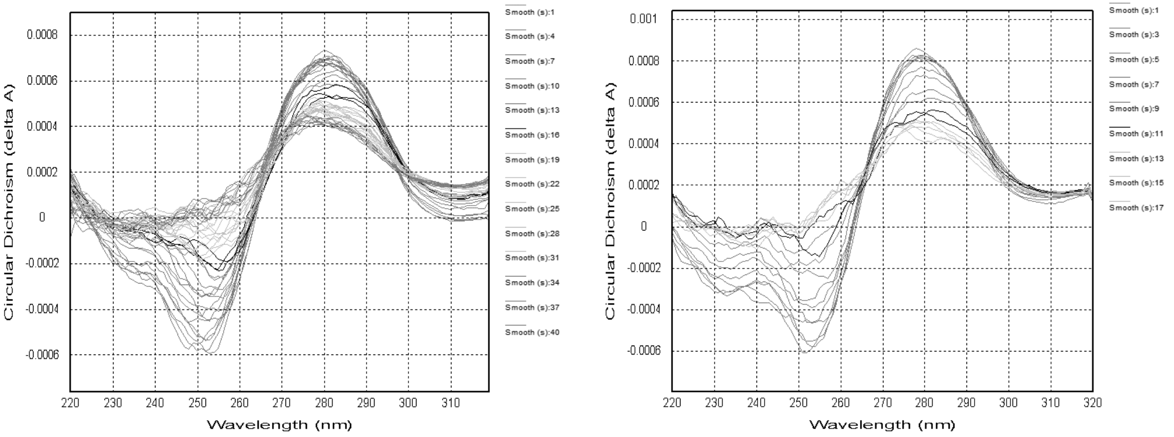

Figure S33. CD spectra of ON(2)•ON(A) (left) and ON(1)•ON(A) (right) (NMR samples) .

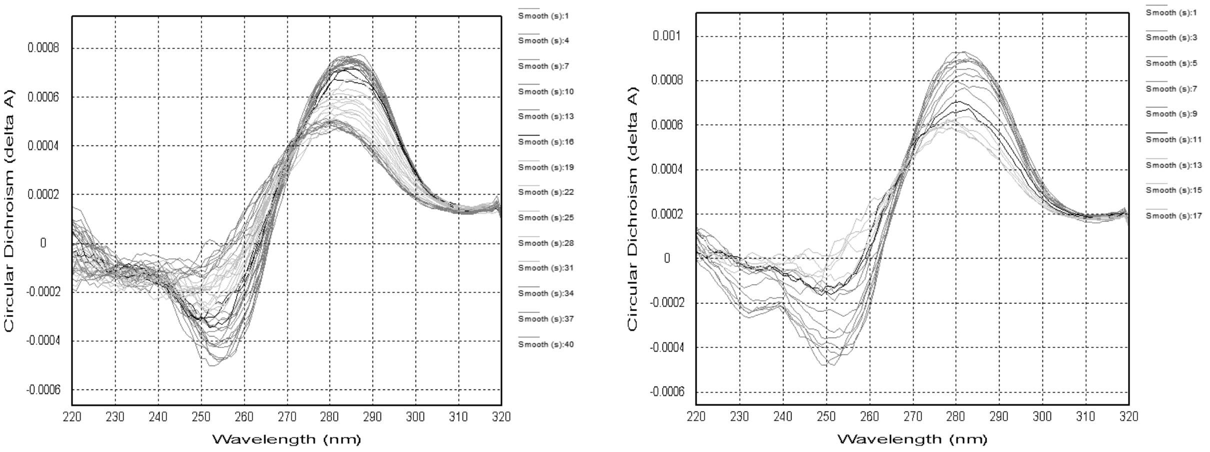

Figure S34. CD spectra of ON(2)•ON(C) (left) and ON(1)•ON(C) (right) (NMR samples).

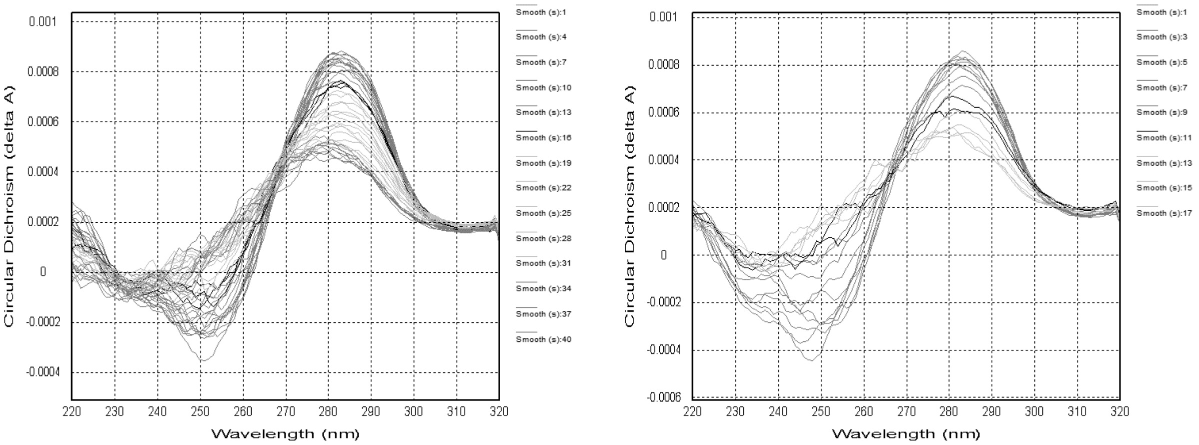

Figure S35. CD spectra of ON(2)•ON(G) (left) and ON(1)•ON(G) (right) (NMR samples).

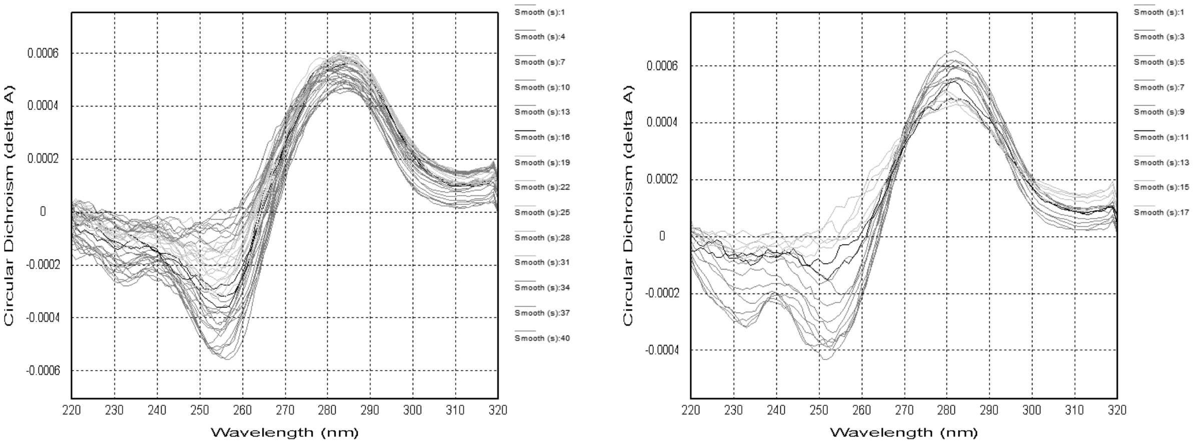

Figure S36. CD spectra of ON(2)•ON(T) (left) and ON(1)•ON(T) (right) (NMR samples).
